# Unveiling the genomic secrets of *Micrococcus luteus* through a comprehensive comparative genomics approach

**DOI:** 10.3389/fgene.2026.1778782

**Published:** 2026-05-07

**Authors:** Ashutosh Kabiraj, Dibyendu Khan, Rajdeep Shaw, Madhushree Ghorui, Rajib Bandopadhyay

**Affiliations:** Microbiology Section, Department of Botany, The University of Burdwan, Burdwan, West Bengal, India

**Keywords:** Actinobacteria, antibiotic resistance, genomics, *in silico*, IS3, KEGG

## Abstract

The central motivation of this study was to analyze the unknown genomic facts of the ubiquitously distributed bacterium *Micrococcus luteus* using *in silico* comparative genomic analysis. A total of 100 contamination-free genomes were retrieved from the National Center for Biotechnology Information (NCBI). Among them, 12 strains from six different isolation sources were selected based on their ANI (average nucleotide identity) values to explore their niche-specific adaptation. Prokka annotation revealed that their total genome size ranged from 2.4 to 2.8 Mb, with plant-growth-promoting and heavy-metal resistance genes prevalent across the strains. There were tryptophan (precursor of indole acetic acid) biosynthesizing genes and *aes* family proteins, indicating its plant-growth promoting traits. Rapid annotation subsystem technology-based functional annotation also detected arsenic resistance (*arsC* and *arsB*), mercury resistance (*merA*, *merB*, and *merR*), and copper homeostasis (*copZ* and *copD*) genes. In-depth accessory genomic analysis revealed attributes such as genomic islands, prophage genomes, metal resistance, hypothetical proteins, insertion sequences (insertion sequence elements), and transposase. IslandViewer 4 identified 21–37 GIs which occupied up to 17% of the total genome. The presence of multiple IS elements was an intrinsic factor of this genome. The number ranged from 28 to 76 under 13 different families, with IS256 the most frequent element. PHASTER analysis identified PHAGE_Paenib_Tripp_NC_028930 as the most dominant prophage among strains with similar GC adaptation. Functional annotation identified 15,444 KEGG orthologs and 6,022 Pfam domains. Comprehensive Antibiotic Resistance Database suggested the antibiotic sensitivity of the species. Finally, despite the prevalent occurrence of *M. luteus*, it is still not multi-drug-resistant and can be applied in heavy-metal-contaminated agricultural field for plant growth promotion.

## 1 Introduction

High-throughput instruments coupled with modern technologies have sequenced a high number of bacterial genomes, enabling us to investigate their genetic cloud. Research on bacterial ecological adaptation strategies have also been assisted by comparative genomics analysis. While the genetic composition of microorganisms is to some extent conserved, the influence of issues such as horizontal gene transfer, genome remodeling, and niche-specific adaptation could also act to change the architecture of their genomes (Li et al., 2021). For instance, *Escherichia coli* possesses more than 1,000 strain-specific genes (Earl et al., 2008), suggesting its genomic plasticity. Comparative genomics analysis is a powerful approach that highlights exact genomic alterations among species, even across strains. Such research is needed to reveal adaptive potential and evolutionary relationships across bacterial strains (Hanafy et al., 2016).

*Micrococcus luteus*, which has the smallest genome harboring high-GC-content Actinobacteria, has been touted as an opportunistic pathogen for human beings. However, this species has a cosmopolitan distribution amongst environments such as amber, the deep sea, deserts, the Mariana Trench, space stations, volcanic sediments, plants, polluted water, and air. It is a typical non-endospore-forming Gram-positive bacterium, but it may enter into a viable but non-culturable (VBNC) state for years to overcome hostile environments, making it one of the most extraordinary microorganisms (Li et al., 2021; Tang et al., 2025). Different strains of *M*. *luteus* have been characterized by researchers to unveil its biotechnological aptitudes, including xenobiotic degradation, plant growth regulation, and heavy metal bioremediation (Pathak et al., 2016; Choden et al., 2025; Partila et al., 2025). Sometimes, *M*. *luteus* performs concurrent functions, like heavy-metal (HM) bioremediation and plant-growth promotion (Choden et al., 2025). Such biotechnological properties are tied to their genomic configurations. In many cases, genes associated with genomic islands, prophage, and other factors (accessory genome) regulate these attributes (Kabiraj et al., 2023). However, there has been inadequate in-depth analysis of *M*. *luteus* accessory genomes and their feasibility in adapting to diverse environmental conditions. Young et al. (2010) was the first to analyze the historically significant Fleming strain (*M*. *luteus* NCTC2665). Several genomic analyses of this bacterium have since been performed, and the amount of genomic data submissions in public databases is constantly increasing. For instance, there were only 15 genomes of different strains of this species submitted in NCBI GenBank in 2016 (Hanafy et al., 2016); a few years later, Li et al. (2021) reported approximately 70 genomes available in NCBI, while recently more than 300 genome sequences were available. Although the repertoire of *M*. *luteus* is very rich, only a few comparative genomics studies have been performed to answer basic questions such as its genomic plasticity. For example, Hanafy et al. (2016) characterized *M. luteus* O'Kane and genomically compared O'Kane with 11 closely related strains of the same species to revealed that O'Kane had many unique genes related to antibiotics resistance. Subsequently, Li et al. (2021) focused on the genomic insights of *M*. *luteus* to demonstrate its ability to deal with diverse environmental conditions, finding several lineages of this bacterium. The pangenome of *M*. *luteus* is open, so the chances of genetic rearrangement through horizontal gene transfer (HGT), recombination, and so forth are very high. Such features are tricky to survive under environmental perturbances.

This current study will focus on an uninvestigated area of genomic research: the niche adaptation strategies of *M*. *luteus*. Since the accessory genome of any bacterium is crucial for its resilience under diverse habitats, special emphasis will be placed on its accessory genomes. Bacterial genomic islands, phage genome particles, and IS elements will be specially considered to gather knowledge on their roles. This manuscript will investigate the genome of *M*. *luteus* to determine the kinds of genetic changes that allow it to thrive in variable environmental niches. As mentioned above, this bacterium was considered an opportunistic pathogen, but maximum strains are susceptible to antibiotics (Kabiraj et al., 2024). We analyzed the average nucleotide identity (ANI) and performed detailed genomic comparisons by selecting 12 genomes of six different environmental niches, with special emphasis on their accessory genetic content, by applying several standard bioinformatics tools. This study intends to answer the following fundamental questions. (i) Is there any original disparity in the accessory genome that aids adaptation to such environments? If yes, (ii) what is the magnitude of difference?

## 2 Materials and methods

### 2.1 Genome retrieval

Genome sequences of all available *Micrococcus luteus* strains were retrieved from (NCBI) GenBank (https://www.ncbi.nlm.nih.gov/genome; June 2022). As a preliminary, genome completeness (≥90%) and contamination free genomes were investigated from the database, and 100 strains were chosen for further study to avoid unwanted ambiguity for genomic comparison.

### 2.2 ANI heatmap study

A pairwise genome comparison between all 100 strains was performed by calculating average nucleotide identity (ANI) values using JSpeciesWS (Richter et al., 2016) (https://jspecies.ribohost.com/jspeciesws/), and an ANI heatmap was generated by the “pheatmap” package of R studio (version 3.0.1). In addition to comparative ANI analysis, digital DNA–DNA hybridization was performed using GGDC (https://ggdc.dsmz.de/ggdc.php#).

### 2.3 Selection of twelve strains for further analysis

On the basis of isolation sources (e.g. air, saline water, soil, contaminated water, plant, and vertebrate), ANI heatmap values, and similarity percentages among the strains, 12 strains were considered for further analysis. After selecting these, genomic features such as GC content, total number of genes, and total base pair were documented. Rapid Annotation Subsystem Technology (RAST) (https://rast.nmpdr.org/) server was utilized for the functional annotation of the genomes (Aziz et al., 2008). Proksee (https://proksee.ca/) was applied to make a comparative account of their gene diversity within a circular view of the genomes.

### 2.4 Comparative core and accessory genome analyses

The whole genome is basically comprised of a core and an accessory genome. Core and accessory genomic contents were investigated using Spine and AGEnt (Ozer, 2018) (http://spineagent.fsm.northwestern.edu/index_age.html). Further core and accessory genomes were individually annotated by the RAST server to clarify what kind of subsystems are distributed within these two major genomes types.

### 2.5 Comparative genomic islands (GIs) and phage genomic parts associated with the genomes

IslandViewer 4 (https://www.pathogenomics.sfu.ca/islandviewer/) (Bertelli et al., 2017) was used to compare gene content within the GIs. The PHAge Search Tool Enhanced Release (PHASTER; https://phaster.ca/) (Arndt et al., 2016) tool was used to screen out the phage genome parts residing within the bacterial genomes as prophages.

### 2.6 Overall gene level comparison among the 12 retrieved genome sequences by considering selected attributes

Genes related to heavy-metal tolerance, plant growth promotion, hypothetical proteins, antibiotic resistance, and the identification of transposable elements were comprehensive investigated by annotating the genomes in different databases and servers, including NCBI, BV-BRC (Bacterial and Viral Bioinformatics Resource Center) (https://www.bv-brc.org/), and RAST (https://rast.nmpdr.org/). Antibiotic resistance genes in genomes were analyzed by the Comprehensive Antibiotic Resistance Database (CARD, https://card.mcmaster.ca/) (Alcock et al., 2023). The content and attributes of insertion sequence (IS) elements within the genomes were analyzed using the ISfinder tool (https://isfinder.biotoul.fr). This study was conducted to gain detailed knowledge about bacterial-niche-specified genetic modifications.

### 2.7 KEGG orthology mapping and pathway annotation

KEGG functional annotation was performed on protein sequences generated through Prokka annotation of 12 *M. luteus* genomes. KEGG ortholog (KO) assignments were obtained using KofamScan with default adaptive score thresholds. For each genome, KO identifiers were extracted and compiled into a binary presence–absence matrix to represent the distribution of KOs across all strains. Orthologs were classified into three categories: (i) “core KOs,” present in all twelve strains; (ii) “accessory KOs,” present in two to eleven strains; (iii) “unique KOs,” present in only a single strain.

### 2.8 Functional domain annotation and comparative proteomic analysis

Protein sequences (.faa) were scanned against the Pfam-A database using the HMMER v3 suite with the hmmscan algorithm. The Pfam database was used with default parameters to identify conserved protein domains based on the hidden Markov model (HMM). Pfam domain counts were compiled into a domain abundance matrix that represented the presence and copy number of domains across all strains. Functional variability among strains was used for comparative proteomic profiling.

## 3 Results

### 3.1 Genome retrieval and ANI heatmap study

**FIGURE 1:**
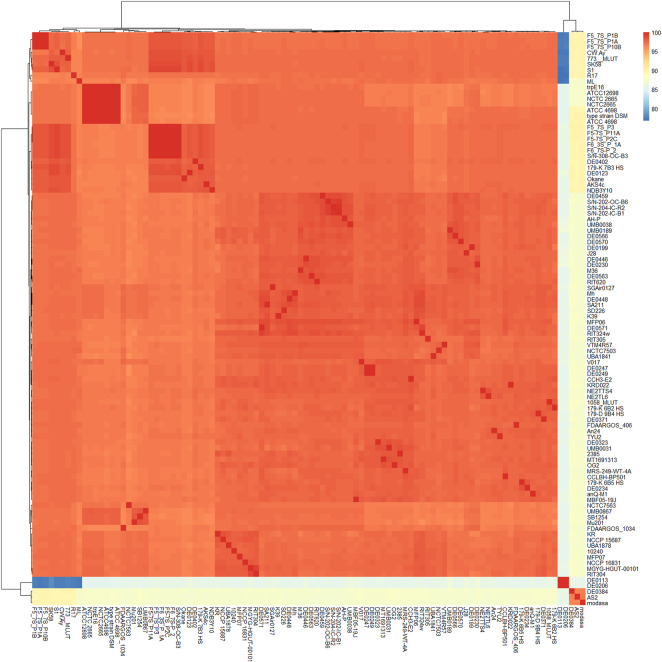
Heatmap showing pairwise similarity or distance among 100 genome sequences of *M. luteus*. Colors range from yellowish to blue, indicating lower values, to red, indicating higher values.

The ubiquitous distribution of this bacterial species is interesting. Members of *M*. *luteus* can be found across nearly every habitat. In the current study, all genomes were primarily retrieved from the NCBI database, but 100 were selected based on factors such as their substantial completeness, good genomic quality, negligible contamination (or contamination-free), and isolation source. Average nucleotide identity (ANI) revealed their inter-strain relatedness. ANI values usually assume relatedness among organisms by calculating the nucleotide similarity of respective core genomes. More than 95% ANI similarity among two strains confirms their designation as the same species (Chaudhry and Patil, 2016). Therefore, values below the threshold cut-off (95% similarity) could see the organisms treated as different species. Strains DE0113 and DE0206 showed approximately 85% similarity (with 15% deviations with the type strain NCTC2665) (Figure 1). Most strains were directly related to *M*. *luteus* (ANI >95%), but the taxonomic positions of strains like DE0113, DE0206, and modasa (shown in blue demarcation in Figure 1) were quite vulnerable. However, more in-depth studies are needed to clarify their taxonomic positions. A detailed account of the features of all genomes is given in Supplementary Material S1.

### 3.2 Selection of 12 strains for further analysis

**FIGURE 2:**
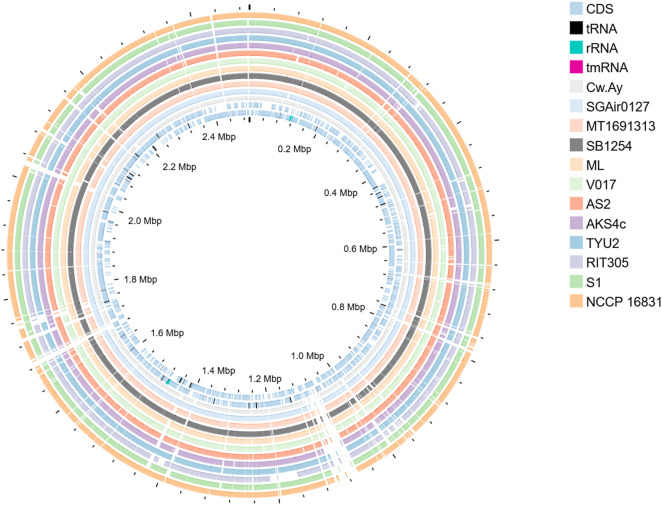
Circular genomic map visualization; each circle is representing one genome. By considering type strain *M. luteus* NCTC2665 as control this comparative map was generated.

On the basis of isolation sources, 12 strains were selected for further analysis. Six isolation sources were considered, and two strains from each source were taken into account. The isolation sources and their respective bacterial strains were as follows: air (Cw.Ay and SGAiR027); saline water (MT1691313 and SB1254); soil (ML and V017); contaminated water (AS2 and AKS4c); plant (RIT305 and TYU2); vertebrate (S1 and NCCP16831). Brief details of the selected strains, including their isolation sources and genomic features, are given in Supplementary Material S2 and Supplementary Table S1. The rationale for the selection of two strains from the same habitat was to investigate their genomic relatedness thriving in similar niche. The genome sizes of strains varied from 2.4 Mb to 2.8 Mb, with 2,249–2,700 protein coding genes (CDS). This bacterium had high GC content (72.50%–73%) (Supplementary Material S2; Supplementary Table S1). A circular view of the selected genomes is provided in Figure 2. Circular views of detailed CDS of the selected strains and a type strain (NCCP2665) are given in Supplementary Material S3 and Supplementary Figures S1–S3.

### 3.3 Comparative analysis of genomic islands

**FIGURE 3:**
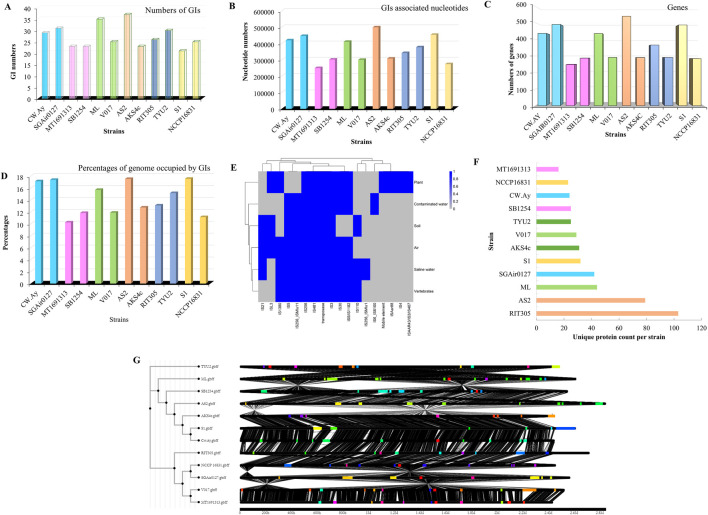
Comparative genomic islands analysis; **(A)** Comparing the numbers of genomic islands in the genomes, **(B)** Nucleotides associated with genomic islands per strain, **(C)** Total gene numbers, **(D)** Percentages of genome occupied by GIs, and **(E)** Presence of transposase across all strains, **(F)** Heatmap representing IS elements occurrence from different isolation sources, **(G**) Bar graph with unique protein count per strain in GIs, **(H)** Synteny comparison of GI regions with the phylogenetic tree mirrors structural divergence within the island.

Genomic islands (GIs) of 12 selected strains were analyzed by IslandViewer 4. Total numbers of GIs, gene diversity, total nucleotides, and percentages of the total genomes which have been occupied by GIs were investigated (Figure 3). The lowest number of GIs was found in strain S1 (GI-21, isolated from dog), followed by AKS4c, MT1691313, and SB1254. Interestingly, all these strains were isolated from water; the highest number of islands (37) was found in AS2, also isolated from water (Figure 3A). An overview of all GIs, their position, genes, and so forth are provided in Supplementary Material S2, Supplementary Table S2, Supplementary Material S3, and Supplementary Figures S5 and S6. Several hypothetical proteins were found in the genomes of all strains. These proteins play a crucial role in bacterial metabolism. A brief account of the GI-associated hypothetical proteins will be discussed below.

A total of 25 GIs was found in strain V017, a soil isolate. The number of GIs and isolation sources are not linearly related; indeed, environmental stimuli may play a major role in the acquisition of GIs. In addition to the number of GIs, their length is also a determining factor to adaptation in a changing environment. Larger GIs contain more genes, which may provide extra benefit to the organism. Large GIs often occupy a significant percentage of the total genome as the accessory genome. For instance, strain S1 had the least number of GIs but was still positioned second (after AS2) in cumulative nucleotide content due to its large GIs (Figure 3B). Moreover, GIs in S1 contained approximately 500 genes, occupying >17% of the genome (Figures 3C, D). The length of any GI is an intrinsic factor for determining the number of nucleotides. S1 had some longer GIs than other strains, covering a central portion of the genome. The largest genomic island was found in S1, approximately 0.15 Mb long. In contrast, the smallest GI was noted in AS2, at only 4000 bp long (harbors only four genes). The total of genes within GIs varied from strain to strain (Figure 3C). The highest number was found in the GIs of AS2 (isolated from contaminated water), while the lowest number was in MT1691313 (isolated from sea water). Another water isolate, AKS4c, showed a significant difference in its gene numbers.

GIs also harbored different IS elements, including IS21, ISL3, IS1380, IS5, IS256, IS481, IS30, ISAaR88, IS4, and IS407 (Figure 3E). Plant isolates had three unique IS elements: ISAar88, IS4, and ISAAR43/IS3/IS407. Soil-associated strains were devoid of IS30 and IS5/IS1182. The GIs of all strains were analyzed to determine the unique protein counts per strain; RIT305 had the maximum number of unique proteins, followed by AS2, ML, and SGAir0127 (Figure 3F).

Surprisingly, 10%–18% of the studied genomes of *M*. *luteus* were occupied by GIs, indicating the importance of these small genomic parts in overcoming stressful conditions (Figure 3D). Synteny analysis confirmed that although GI architecture was conserved across the strains, the presence of multiple strain-specific insertions, deletions, and gene rearrangements reflects ongoing mosaic evolution. The phylogenetic tree indicating strains with a similar GI structure cluster together—for example, AKS4c was closer to AS2 and S1 and V017 clustered with MT1691313 (Figure 3G).

The disparity in the distribution of genes was common across strains. For example, only Cw.Ay, AKS4c, AS2, and SB1254 harbored arsenate reductase (*arsC*); among them, AKS4c, AS2, and SB1254 were isolated from water. Moreover, *arsC*, harboring genomic islands, also bore other arsenic resistance genes (*arsR*, *arsB*, etc.) of the *ars* operon. This finding suggests that horizontal gene transfer (HGT) may accumulate a full cassette of genes, with operons instead of selection of desirable gene(s). The GI is one of the most crucial parts of the accessory genomes of bacteria; a bacterium itself could modulate or change its genomic content based on its surroundings. For instance, both strains AS2 and AKS4c were isolated from arsenic-contaminated water, and they had *ars* operons in their genomic islands, indicating their ability to frame genetic content.

### 3.4 Comparative genome analyses

**FIGURE 4:**
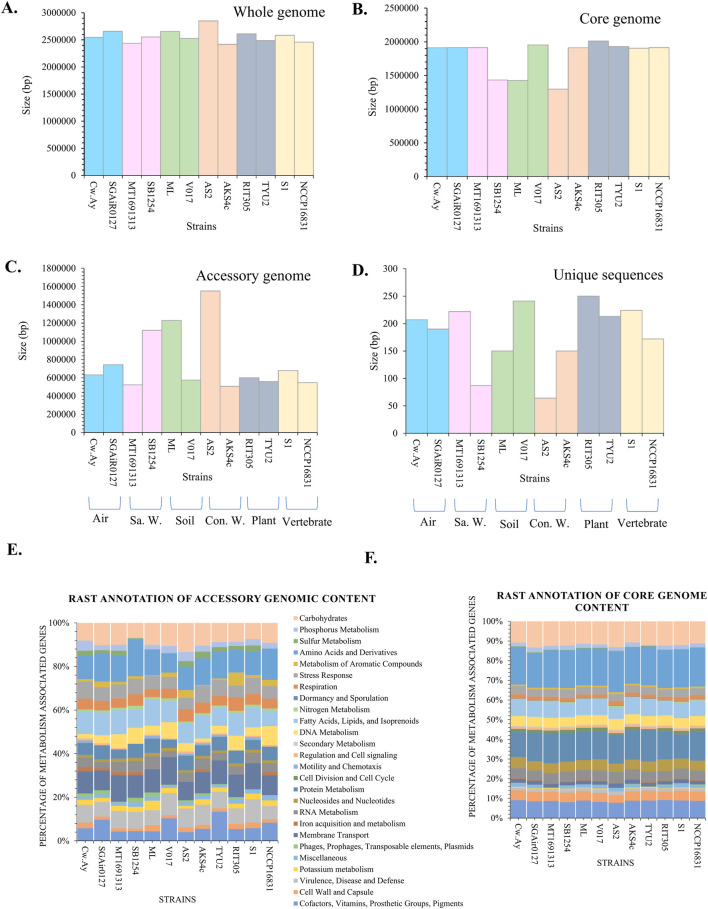
Comparative whole genome analyses of the selected strains of *M. luteus*; **(A)** Comparing whole genome sizes in base pairs across twelve strains; **(B)** Core genoe size; **(C)** Accessory genome size and **(D)** Unique sequence sizes for each strain, **(E,F)** RAST annotation of core and accessory genomic contents of the strains of *M. luteus*.

*M. luteus* has a smaller genome than many other bacterial species. Each genome consists of only 2.4–2.8 Mb DNA. The functional annotation of genomes by RAST identified a group of subsystems (Supplementary Material S3; Supplementary Figures S7–S12). An overview of total genomic content, core genome, accessory genome, and unique genes is graphically represented by Figure 4. The lengths of the whole genomes of the selected strains were approximately same (Figure 4A). However, strain AS2 had larger genome (2.8 Mb) than others. A whole genome is comprised of the core and accessory genomes. Core genomes are conserved genomes for a bacterial species, with a set of genes that are responsible for maintaining general metabolisms. Here, the length of nine core genomes of *M*. *luteus* were nearly the same (“conserved” at their species level), but lesser core genomic contents were documented in SB1254, AS2, and ML (Figure 4B). Genome length along with function and number of genes may vary across strains of the same species. Environmental conditions are the final selection pressure which requires bacteria to edit their genomes. The current study found variation in accessory genomic contents (Figure 4C). The accessory genome content is dependent on the GIs and phage-particle-assisted genomes. An account of GIs was given above, while the prophage genome will be discussed below. A glimpse of a “unique genome” (i.e., unique sequence for each strain) is also given in Figure 4D. Unique genomic content was very small for each strain of *M*. *luteus*.

Further core and accessory genomes were analyzed through the RAST server to identify genes related to different metabolic pathways (Figures 4E, F). Except for AS2, the functional attributes of the core genomic content of the strains were quite similar (Figure 4E) while appearing very dynamic in accessory genomes (Figure 4F). The accessory genome helps in overcoming environmental stress. Hence, the presence of desirable accessory genes within genomes of bacteria provides extra protection against stressors. Therefore, the availability of a higher percentage of accessory genomes in strains like AS2, SB1254, and ML may help them thrive successfully in their respective niches.

#### 3.4.1 Comparative analysis of heavy-metal resistance genes in selected strains

**FIGURE 5:**
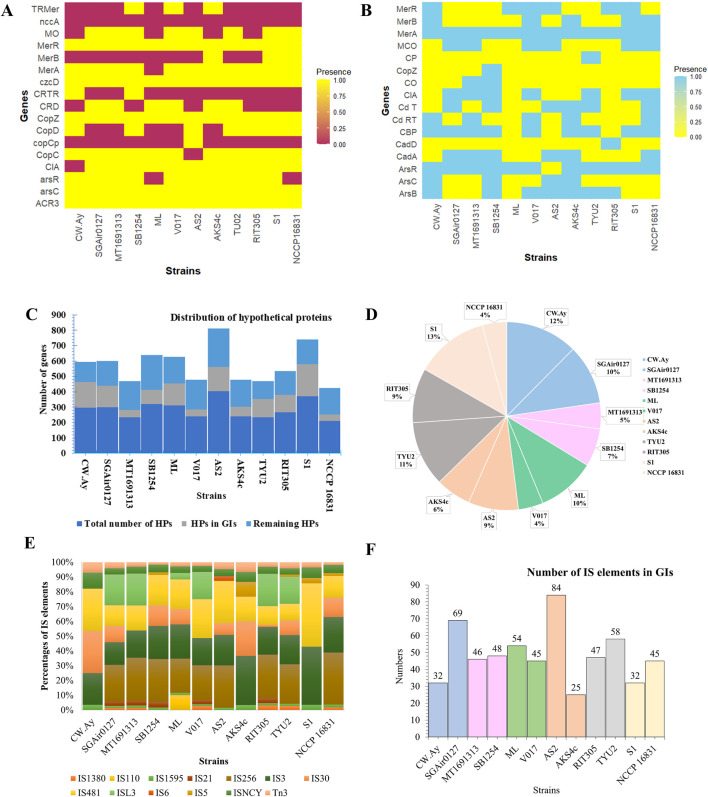
**(A)** Heatmap of the diversity of heavy metal resistance genes in core genomes. **(B)** Diversity of heavy-metal resistance genes in accessory genomes. **(C)** Distribution of hypothetical proteins. **(D)** Percentages of total genomic area occupied by hypothetical proteins. **(E)** Distribution of IS elements in the strains. **(F)** IS elements in the GIs of bacterial strains.

Heavy-metal (HM) resistance genes within the core genome of bacterial strains were analyzed to obtain information on the genetic variation across strains isolated from various sources (Figure 5A). Special emphasis was given to arsenic, copper, mercury, and cadmium resistance genes. Here, cytosolic iron-sulfur cluster assembly (CIA) machinery was the most common (3–8 genes) among the strains (Supplementary Material S3; Supplementary Table S1). Surprisingly, Cw.Ay (isolated from air) was devoid of CIA. Gene *nccA* was only present in strain AKS4c, which encodes a HM efflux pump that governs metal resistance. Nickel sometimes upregulates its (*nccA*) expression in bacterial cells. In addition to *nccA*, other HM (oid) resistance genes like *arsC*, *arsR*, *acr3*, *merR*, *merA*, and *merB* were also found within the genomes of *M*. *luteus* (Figures 5A,B). *merB* encodes organomercurial lyase (MerB) that reduces the toxicity of organic mercurial compounds by converting it to less toxic mercury cation (Hg^2+^), and later Hg^2+^ is transformed to volatile elemental Hg (0) by MerA. A transcriptional activator (MerR) regulates this process (Norambuena et al., 2020; Krout et al., 2022).

Surprisingly, the distribution of HM resistance genes varied among strains (Figures 5A, B). *merR* was common to many strains, while *merA* was absent in ML, and *merB* was found only in AKS4c, S1, and NCCP16831. Thus, only these three strains were capable of catalyzing the reduction of organic mercury to a volatile one. As ML has no *merA*, this strain might not be able to convert cationic (Hg^2+^) to elemental mercury Hg (0). Other strains could not encounter organic mercury because they lacked *merB*, the first enzyme for bacteria-mediated mercury biotransformation. In case of the *cop* operon, *copZ* was distributed in all genomes, *copC* was absent in AS2, and *copD* was absent in SGAir027, MT, ML, V017, and AKS4c. All these genes regulate copper homeostasis in the bacterial cell, so a tiny fluctuation in copper concentration could harm bacteria, as such genes were absent in the above mentioned strains. Arsenic regulatory protein encoding gene (*arsR*) was absent in ML and NCCP16831. However, other genes were more or less present in their genome. Efflux pump (*arsB*) was common, indicating that this group of bacteria might efflux metallic cations instead of transformation. Other efflux pumps were also noticed in these strains.

Among the strains, V017 (isolated from soil) harbored the most HM resistance genes in its core genome, and least was found in ML. ML (from soil) had also acquired a few (03) HM resistance genes in the environment from genomic islands. In this case, selection pressure could be the driving force that regulates horizontal gene transfer. Overall, there was no bias of HM resistance genes in respect to isolation sources.

Amongst the 12 strains of *M*. *luteus*, SB1254 had the most HM resistance genes (10) in their genomes, providing copper, cadmium, arsenic, and mercury resistance to their genomic islands (GIs). This strain was followed by V017, AKS4c, and NCCP16831, which harbored nine genes for resisting these four toxic elements while the fewest was found in ML (Figures 5A, B). Three mercury-resistant genes (*merA*, *merB*, *and merR*) were situated here in the GIs of Cw.Ay, V017, and S1. *merA* was present in all strains, but the presence of the other two genes varied greatly across strains. Although the core genome of ML lacked *merA*, it had *merA* in its GI along with a regulator-protein-coding gene (*merR*), suggesting a collaborative approach between the core and accessory genome to resist HMs. Like the core genome, the accessory genome of SB1254 had a significant number of copper homeostasis genes, indicating its attribute of high copper resistance and probable applicability in copper bioremediation. Several copper resistance genes were absent in the GIs of strains Cw.Ay, ML, and AS2. Only strain RIT305 had *cadD* for cadmium resistance. The *ars* operon with three gene sets (*arsR*, *arsB*, and *arsC*) was documented in the GIs of Cw.Ay, SB1254, and AKS4c (an arsenic-resistant strain).

#### 3.4.2 Distribution of hypothetical proteins in genomes

The number of hypothetical proteins (HPs) varied from 405 (AS2) to 212 (NCCP16831) (Figure 5C) across strains. Many genes in the GIs were HPs. S1 had the most HPs (209) in GIs, and the fewest were documented in NCCP16831 (40). This result was also followed by the percentage occupied by HPs: S1 (56.48% of total genes of GIs), with the lowest being NCCP16831 (18% of total genes of GIs). Presence of more than 56% of total proteins in GIs were HPs, indicating their importance in adaptation to environmentally stressful conditions. However, we are still far from identifying the actual function of these proteins. S1 was isolated from an animal (dog), but the isolation source of strain NCCP16831 was human. Interestingly, both the highest and lowest number of HP-containing strains (in GIs) were isolated from animals, indicating an absence of bias toward isolation sources. Unlike HMs, however, there were similar numbers of HPs within the whole genome between two strains thriving in the same niche (e.g. air, tree, etc.) (Figure 5C). For instance, air-inhabiting strains Cw.Ay and SGAir027 had 297 and 300 total HPs, respectively. This suggests that HPs in a continually changing accessory genome (here GIs) may vary, but in the context of the whole genome, it is more or less stable. Hypothetical proteins accounted for 13% of total genes of S1 (Figure 5D). More detailed structural and functional analyses of these proteins would reveal the proper scenario.

#### 3.4.3 Distribution of IS elements

Among 12 selected strains, the most IS elements was found in strain V017 (76), followed by SGAir0127 (72) and TYU2 (71), while the fewest were documented in Cw.Ay and S1 (28) (Supplementary Material S3; Supplementary Table S2). Therefore, V017 had three times more IS elements than S1, suggesting its genomic plasticity. We noticed 13 different IS elements within the genomes of *M*. *luteus* (Supplementary Material S3; Supplementary Table S3).

There were 163 number of IS256, 145 number of IS3, and 134 number of IS481 family IS elements within the genomes of selected strains (Figure 5E). Generally, members of the IS256 family were the most frequent elements in *M*. *luteus* genomes; however, strains like AKS4c, Cw.Ay, and S1 lacked IS256. Only two copies of IS6 were present in the genome of AS2. An investigation of the sources of IS elements within the genomes of selected strains confirmed that most of their elements originated from Actinobacteria. However, in a very few cases, members of Proteobacteria and Firmicutes were also found to be acting as an “origin” of several IS elements. Except for S1 and RIT305, the rest had at least one or two elements originating from phyla other than Actinobacteria. *M*. *luteus* had a clear bias to *Arthrobacter* for acquiring IS elements, so *Arthrobacter* acted as the “origin” of most IS elements of *M*. *luteus* (Supplementary Material S2; Supplementary Table S3). The threshold E-value was standardized to ≤ 10^–10^ to predict IS elements. Element names, origins, E-values, and other details of all strains are given in Supplementary Material S2 and Supplementary Table S4. Among all IS elements, IS256 was the most abundant.

Transposase-encoding genes and their families were identified. Strain AS2 harbored 84 transposases, followed by SGAir0127 (69) and TYU2 (58), while the fewest were found in strain AKS4c (25) (Figure 5F). The most abundant families of transposases in *M*. *luteus* were IS3, IS5, IS481, and IS256; these are usually abundant in prokaryotes (Tempel et al., 2022). Only RIT305 had the IS4 type of transposase, while other strains were devoid of it (Supplementary Material S3; Supplementary Table S3). The IS3 family transposase resembles retroviral integrase catalytic core and is significant in prokaryotic evolution (Siguier et al., 2014).

#### 3.4.4 Comparative analysis of plant growth-promoting (PGP) genes

Strains RIT305 and TYU2 were isolated from plant species. Both strains were endophytic in nature; the former was isolated from *Salix* sp. and the latter from *Taxus* sp. (Supplementary Material S1; Supplementary Table S1). An overview of PGP genes is given in Supplementary Material S3; Supplementary Table S4. This table was prepared by considering the known PGP attributes of this bacterium, such as indole acetic acid (IAA) biosynthesis, phosphate solubilization, and siderophore production. The *aes* family protein-encoding gene was present in almost all strains (excepting AS2 and V017). These proteins export IAA from bacterial cells to make it available to plants. Strains such as RIT305 and ML harbored an extra copy of *trpB* (encoding tryptophan synthase, the last enzyme for tryptophan biosynthesis). The gene *pstS* was absent in SB1254, AS2, and AKS4c—the latter two strains were isolated from metal contaminated water. This study also encountered another phosphate transporter gene (*phoH*) which seems to be an indirect regulator of phosphate concentration in bacterial cells, but its detailed function has not yet been uncovered. SB1254 (isolated from saline water) was devoid of this gene (other strains had two copies per genome). In contrast, a couple of other phosphate regulatory proteins (extracellular alkaline phosphatase, PhoX) were detected in endophyte RIT305 (Supplementary Material S1; Supplementary Table S5). Denitrification, nitrogen-fixing, and nodulation-forming genes were totally absent in the genomes of selected strains. Only the phosphate solubilizing gene, peptide chaperone (*pqqD*) was present in all strains. This gene is critical for synthesizing pyrroloquinoline quinone (PQQ), which eventually may take part in phosphate solubilization (Chen et al., 2024). The IucA/Iucc family siderophore biosynthesis protein was only found in strain AKS4c. In addition, several siderophore-producing and aromatic-compound-degradation related genes were documented in all strains of *M*. *luteus*.

#### 3.4.5 Determination of bacteriophage particles in strains

The PHASTER tool identified several phage genomic particles integrated within bacterial genomes (Supplementary Material S3; Supplementary Figures S13–S18). Among several intact, incomplete, and questionable phage genomic contents, most available and important bacteriophages are tabulated in Supplementary Material S3 and Supplementary Table S6. We intended to identify whether there was any kind of specificity of bacteriophage(s) in relation to their isolation sources. Interestingly, the same kind of bacteriophage genomes were detected in strains isolated from vertebrates (the most common was PHAGE_Paenib_Tripp_NC_028930). However, this phage virus particle was also detected from isolates of sea water, soil, and plants, indicating its affinity with this bacterium regardless of isolation sources. This bacteriophage usually infects *Paenibacillus* sp., and the current study identified it as the most abundant phage. Approximately 41 Kb of intact phage genome (with 50 protein coding genes) was found in SB1254. Another strain, AS2, bore 40.90 Kb of genome with 49 protein-coding genes. The lowest gene content was noticed in air (6), which contained only 7.5 Kb of incomplete genome (Supplementary Material S3; Supplementary Table S6). SB1254 (sea water isolate) and ML (from soil) had the highest cumulative number of phage particle genes. Surprisingly, the integrated phage genomes (62%–73% of GC) and their hosts (>70%) both have high GC content. It could thus be anticipated that the bacterium may permit infection with a compatible phage virus or vice versa, and the GC content of the phage genome is a distinguishing attribute. This result supports previous studies, such as on *Bacillus pacificus*, a low GC%-containing bacterium which selected bacteriophages with lower GC content (Kabiraj et al., 2023). SB1254, NCCP16831, TU02, and AS2 had intact phage genomes. SB1254 had 17 identical phage elements integrated within its genome. The isolates from sea and contaminated water had the most prophage genomes, while the least was present in airborne strains. Basically, the phage genomes had transposase and phage particle encoding genes. However, like GIs, hypothetical proteins were abundant in prophage particles.

#### 3.4.6 CARD analysis to decipher antibiotic resistance genes

**FIGURE 6:**
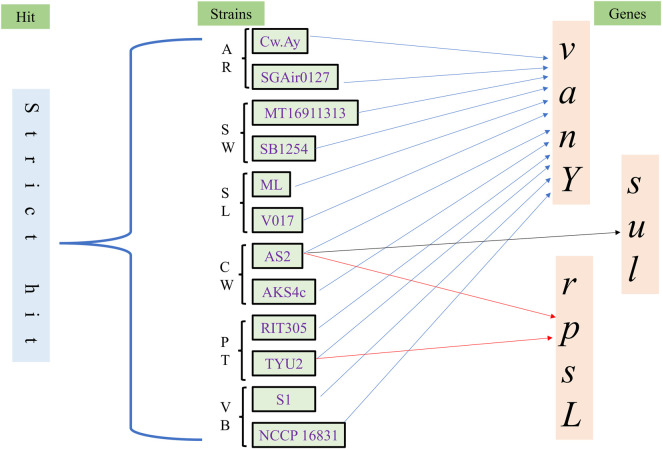
Diagram of CARD analysis. Only strict hits are considered. Gene *vanY* was the most common, present in all genomes. Strains AS2 and TYU2 had *rpsL*, and only AS2 bore *sul*. These genes drive antimicrobial resistance (isolation sources are represented by abbreviation: AR = air, SW = saline water, SL = soil, CW = contaminated water, PT = plant, VB = vertebrates).

The Comprehensive Antibiotic Resistance Database (CARD) was utilized to screen out probable antibiotic resistance genes (Figure 6). This database predicts resistance genes using Resistance Gene Identifier (RGI) criteria, and it divides the results into three basic categories: perfect, strict, and loose hits. A “perfect hit” confirms resistance genes, while “strict hit” is an indication of nearer but not confirmed matches with the predicted gene; “loose hit” is much less significant than these. In the current analysis, no perfect hit was found. Only one to three genes were predicted as strict hits by this tool (Supplementary Material S3; Supplementary Table S7; Supplementary Figures S19,S20). Therefore, *M*. *luteus* genomes had very rare antibiotic resistance genes. The identification of matching regions varied 30%–100%, but only three strict hits had more than an 80% identity of matching regions. According to the strict hit prediction, strains TYU2 and AS2 had antibiotic-resistance *rpsL* that is basically associated with streptomycin resistance. AS2 contains *sul1*, which is dedicated to resisting the sulfonamide class of antibiotics. Other bacterial strains had *vanY* resistance genes only. *vanY* provides resistance to the glycopeptide class of antibiotics by altering the target of interaction. Among the genes studied, only *sul1* had the capability of target-site replacement of antibiotics. Overall, this bacterium is devoid of this kind of antibiotic resistance genes, so it could be applied in medical science as a model organism.

#### 3.4.7 KEGG orthology mapping and pathway annotation

Comparative functional profiling of 12 *S. hominis* genomes identified a total of 15,444 KEGG orthologs across the dataset (Supplementary Material S4; Supplementary Table S1). Functional classification revealed a dominant conserved component, with 10,085 (65.3%) orthologs constituting the core functional repertoire shared by all strains (Supplementary Material S4; Supplementary Table S2). In contrast, 3,734 (24.2%) orthologs were classified as accessory (Supplementary Material S4; Supplementary Table S3), exhibiting variable distribution across genomes. Additionally, 1,625 (10.5%) orthologs were strain-specific and defined the unique functional fraction (Supplementary Material S4; Supplementary Table S4).

**FIGURE 7:**
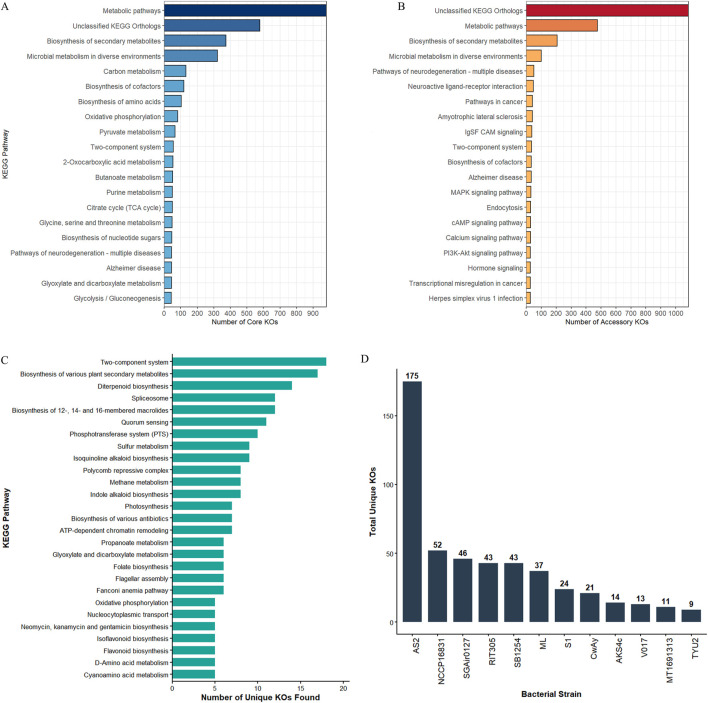
KEGG pathways mapping and functional annotation. **(A)** Top 20 core metabolic pathway shared by all 12 strains. The dominance of the central carbon metabolism underscores the metabolic efficiency of *M. luteus*. **(B)** Distribution of top 20 accessory pathways present in multiple, but not all, strains. **(C)** Bar chart representing top 25 KEGG pathways ranked by the number of unique KEGG Orthologs (KOs) identified across all strains. **(D)** Strain-wise distribution of unique KOs, showing how specific genomes have been enriched with unique KOs, likely via horizontal gene transfer, to provide a competitive edge in their specific ecological niches.

KEGG functional annotation results were divided into four separate categories: core pathways, accessory pathways, unique pathways, and a representation of total unique KEGG orthologs (KOs) (Figure 7). In both core and accessory pathways, a considerable proportion of KOs remain categorized as “unclassified KEGG orthologs”, indicating a large fraction of genes with no valid functional assignments. Core KOs were predominantly associated with fundamental metabolic pathways such as carbon metabolism, pyruvate metabolism, citrate cycle, and glycolysis/gluconeogenesis (Figure 7A). Additionally, the carotenoid and terpenoid biosynthesis pathway was also observed. The core pathway was also enriched with ABC transporters and peptidoglycan biosynthesis genes (Supplementary Material S4; Supplementary Table S2). In contrast, accessory KOs exhibited (Figure 7B) only 40% of pathways overlapping with core KOs. While core KOs are involved in crucial metabolic pathways, accessory KOs dealt with several cell signaling and interaction functions (like cAMP, calcium signaling, and two component system), xenobiotic degradation (chloroalkanes, naphthalene, benzoate, etc.), and alternative carbon utilization pathways (Supplementary Material S4; Supplementary Table S3). The most interesting result was noted in unique KOs, with several impactful pathways that were directly or indirectly related to niche adaptation (Figure 7C). A two-component system was the most frequent unique KO across strains, followed by the biosynthesis of plant secondary metabolites. Several unique KOs were associated with bioremediation (e.g., K14421, K18243, K16243, and. KO7420), adaptation to harsh environments (e.g., K28536, K28537, and K02048), competitive fitness (K13385, K14975, K12708, K12711, and K12723), and genetic plasticity (K26961, K26962, K26805, K26806) (Supplementary Material S4; Supplementary Table S4). This indicates that HGTs play a pivotal role in the functional diversification of these isolates. Further analysis of strain-specific unique KOs showed a wide range of difference, with AS2 having the highest unique pathways (175) and TYU2 containing only nine (Figure 7D), demonstrating significant heterogeneity in functional potential across strains.

#### 3.4.8 Functional domain annotation of proteins and comparative proteomic analysis

**FIGURE 8:**
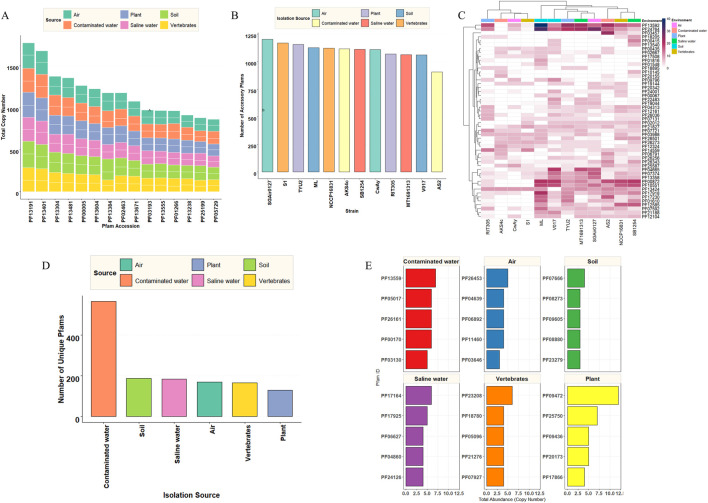
Distribution of Pfam domains across genomes. **(A)** Source-wise distribution of top 15 core Pfam domains found across the sources. Each color represents the isolation source with two strains. **(B)** Number of accessory Pfams found among all the strains. **(C)** Heatmap showing the distribution of the most variable accessory Pfam domains across genomes. Rows represent Pfam domains and columns represent individual genomes. Colored annotation bars indicate the ecological source of each genome. Clustering pattern highlights functional diversification among isolates. **(D)** Bar plot showing the total number of unique Pfams with different ecological sources. **(E)** Distribution of source-specific Pfam domains across the analyzed genomes. Bar chart displays the number of unique domains associated with each source.

Functional domain analysis identified 6022 Pfam domains across the dataset. Based on their occurrence across the genomes, 2504 Pfam domains were identified as core domains (Supplementary Material S4; Supplementary Table S5), 2116 as accessory (Supplementary Material S4; Supplementary Table S6), and 1402 Pfam domains were identified as unique (Supplementary Material S4; Supplementary Table S7). Analysis of the conserved protein domains revealed PF13191 as the most dominant, followed by PF13401, PF13304, and PF14481 (Figure 8A). It was found that the AAA+ protein superfamily was the most abundant and helps in the energy-dependent remodeling or translocation of macromolecules. Almost all of these top families (in copy number) belong to the P-loop NTPase clan, meaning that they use the energy from ATP hydrolysis to induce conformational changes in other molecules. Other core Pfams were related to transport, efflux, molecular chaperones, remodeling, genome integrity, and defense. An air isolate (strain SGAir0127) bore the highest number of accessory Pfams (Figure 8B). Surprisingly, AS2 had the fewest accessory Pfams. Earlier, we found that the greatest number of unique genes were present in AS2; thus, the diversity of Pfams was less in this strain. Accessory Pfam distribution revealed that PF13592 and PF24764 had the highest presence in soil isolates (strain ML). This DNA-binding helix-turn-helix domain is often found in transferases. Two soil isolates (ML and V017) were clustered together in terms of accessory Pfam content (Figure 8C). Isolates of contaminated water had three times more unique Pfams than others. The plant isolates had the least number of unique Pfams (Figure 8D). Although source-specific Pfams were also found, most Pfams were classified as domains of unknown function. However, the tetrahydromethanopterin S-methyltransferase domain (PF09472) was the most frequent Pfam domain in plant isolates and the *M. penetrans* paralog family 26 (PF07666) was most frequent in soil isolates (Figure 8E).

## 4 Discussion

*Micrococcus luteus* is often considered an opportunistic pathogen. However, the last common ancestor of this bacterium was obligately free living. Recently, a few lineages have opted for a mammal-associated lifestyle, and the process of its adaptation is underway (Li et al., 2021). Its ubiquitous distribution is a well-established fact (Li et al., 2021; Shi et al., 2023). Fortunately, a great number of genome sequences of *M. luteus* have been submitted to NCBI, presenting an opportunity to study their genomic diversity, adaptation capability, habitat-dependent selection pressure. To date (December 2025), 463 genomes (including draft, contaminated, and complete) have been submitted to this database, bringing a large set of genomes to the attention of this study. While the core genome was taken into account for ANI analysis, the whole genome (including core, accessory, and unique genes) was considered for dDDH-based taxonomic position evaluation. The threshold value for species identification by ANI is 95%, while dDDH is 70% (Chaudhry and Patil, 2016). The core genome part helps the investigation of interrelationships, while variable regions illustrate their origin, adaptation, and evolution. Figure 1 suggests that some strains had lower ANI similarities. The 12 selected strains had good ANI values that may appear in the same species rank.

CIA, a heavy metal resistance gene cluster, was found in the genomes of these strains. In eukaryotic systems, CIA generally helps to produce Fe-S clusters. However, homologs of eukaryotic CIA are also found in bacterial genomes (Aubert et al., 2023) and sometimes exhibit pathogenicity, as noticed in *Campylobacter jejuni* (Neal-McKinney and Konkel, 2012; Soren et al., 2025). Fe-S clusters have several functions in biological systems, including antibiotics and metal resistance. Therefore, bacteria with CIA biosynthesizing genes could withstand metal stress (indirectly) by expressing Fe-S cluster forming genes. The relationship between bacteria and heavy metals is very intricate because a trace concentration of some heavy metals (copper, zinc, cobalt, iron, etc.) is essential, but more than their permissible concentration could be lethal. Copper plays multiple metabolic functions in bacteria, while its concentration in cytoplasm is critically regulated by proteins of the *cop* operon. The periplasmic copper homeostasis protein (CopC) and transmembrane protein (CopD) collaboratively import copper in cytoplasm, while excessive copper is removed by CopA (ATP-driven efflux pump). Simultaneously, CopZ (a chaperone) assists CopA by bringing excess copper nearer to this pump (Smaldone and Helmann, 2007; Majhi et al., 2023). Another plasma-membrane-integrated protein, CzcD—a cation diffusion facilitator (CDF) protein family transporter—partially reduces the concentration of toxic metal ions such as cadmium (Cd^2+^) or effluxes excess essential heavy metals, including Zn and Co (Liu et al., 2021). Although trace amounts of Zn and Co are crucial for normal bacterial functions, cadmium has no positive role in the survival of any organism. As this pump could efflux several metals, it might have the selective capability of removing metals from cells by distinguishing their impacts on cellular processes.

High resistance to several metals by *M. luteus* has been reported by scientists throughout the world. Since the first report on the Fleming strain’s genome (Young et al., 2010), the genetic regulation of metal tolerance has been intensified by some groundbreaking studies. The smallest genome-bearing Actinobacteria with diversified metal resistance genes are interesting attributes of *M*. *luteus*. Previously, multi-metal resistance by many strains of this bacterium has been investigated (Sher et al., 2020; Arroyo-Herrera et al., 2023; Kabiraj et al., 2024; Vaishnav et al., 2024). In terms of metal resistance, the gene pool of this species is very enriched, and sometimes multiple sets of genes for single-metal resistance can be found. For instance, Sher et al. (2020) identified several arsenate reductases (*arsC*) scattered on the genome of strain AS2. Members of this genus were able to resist higher concentrations of heavy metals or metalloids such as arsenic, copper, cadmium, zinc, cobalt, and nickel (Sher et al., 2020; Arroyo-Herrera et al., 2023; Kabiraj et al., 2024). Genome mining has revealed arsenic-resistant genes are ubiquitously distributed across the strains of *M. luteus*, suggesting their reliance on metal resistance genes to overcome arsenic toxicity. In contrast, a chromate-resistant strain lacked a dedicated chromate resistance gene but it tolerated high concentration of chromate in the laboratory (Vijay et al., 2017). They also observed that antioxidant-producing genes were upregulated by the bacterium to deal with chromate stress (Vijay et al., 2017). Such metabolic versatility supports the genetic strength of this bacterial group to cope with metal stress, even in the absence of respective genetic elements. AKS4c had some antioxidant encoding genes in its genome (Kabiraj et al., 2024). Like chromate, the same observation was also reported in arsenic resistance. Da Costa et al. (2018) noted that the Antarctic bacterium *Exiguobacterium antarcticum* strain B7 was devoid of the well-known arsenic resistance genes but still tolerated high concentration of arsenite and arsenate salts. According to their observation, hypothetical proteins have some role in stress alleviation. So, we will now discuss the distribution and impact of hypothetical proteins.

Hypothetical proteins (HPs) are often associated with several metabolic functions, but unfortunately their roles are yet to be fully revealed. The pangenome of *E*. *coli* has approximately 10% of HPs (Vincent, 2024). Their number varies from species to species, sometimes across strains of the same species. An account of HPs in pathogenic bacterial genomes has been provided by Omeershffudin and Kumar (2019), who reported that approximately 650 hypothetical protein-coding genes were present among 1,693 CDS (>38%). Applying some bioinformatics approaches, this research team has uncovered the probable functions of HPs. In another study, functional analyses of HPs in *E. antarcticum* strain B7 notably showed three HPs to be responsible for arsenic resistance (da Costa et al., 2018). This study supports the interference of HPs in the multi-metal tolerance capability of *M*. *luteus* strains. Although comprehensive studies on HPs of *Staphylococcus aureus* have been conducted, the HPs of *M*. *luteus* have not been fully elucidated. The length of HPs varies remarkably. Previous studies have identified a significant percentage of HPs within the accessory genome of *M*. *luteus*, mostly in GIs. Genome mining has revealed that strain AKS4c harbored many uncharacterized hypothetical proteins (Kabiraj et al., 2024). Overall, the abundance of HPs in both GIs, phage genomes, and the core genome of strains is a relatively untouched area of research.

*M*. *luteus* has an open pangenome that offers rigorous mutations to its genome in terms such as duplication, deletion, and genetic rearrangement. Consequently, many IS elements have been detected. On the other hand, IS elements help alter genomic architecture. As per previous reports, Tn3 element was absent in the Fleming strain (Young et al., 2010). However, in this study, all strains bore IS elements of the Tn3 family. Similarly, members of the IS1380, IS1595, IS6, and ISNCY families were absent in the Fleming strain (Young et al., 2010), but these elements had identified here. The IS elements actively interfere with cellular metabolisms. For instance, ISMlu8 (a transposase) was deleted from the *crt* biosynthetic operon (encodes genes for producing yellow-colored pigment) of *M*. *luteus*. As a result, the yellow color of *M*. *luteus* was absent in the mutant strain (Barker, 2022).

Transposable elements are firmly associated with the development of virulence, antibiotic resistance, adaptation to specific niches, and so forth. However, most of the strains of this species are surprisingly susceptible to antibiotics (Zhu et al., 2021; Kabiraj et al., 2024) regardless of clinical strains (Zhu et al., 2021). The *icaADBC* operon is generally associated with human pathogenicity (Kozitskaya et al., 2004). Strain NCCP16831 was isolated by Lee et al. (2020) from the blood sample of hospital patients in South Korea and bore this operon. In contrast, another animal isolate (S1) lacked it. Elements such as IS3 and IS481 were common in the genome of selected strains. IS481 is frequently found in pathogenic strains of *Bordetella* (D’Halluin et al., 2025), but there is yet no detailed investigation on the function of IS481 in *M. luteus*. However, the genome of the Fleming strain bore this element (Young et al., 2010).

In addition to IS elements, transposase-encoding genes in the accessory genomes (in GIs) of the selected strains were also analyzed. Transposases could act as an integral part of some transposable elements which, in turn, can modify genomic architecture by gene duplication, enabling genetic recombination, disrupting functional genes, and possibly interfering with the metabolisms of organisms by influencing or silencing the expression of desired genes. In an assertive sense, the presence of transposase is an indication of an organism’s heightened stress resilience, but these enzymes may also be detrimental when they inhibit the function of essential genes. Although these elements are ubiquitously distributed across organisms, their abundance varies greatly, ranging from zero to multiple numbers (Vigil-Stenman et al., 2017).

From the perspective of ecological adaptation and evolution, the repertoire of transposable elements in bacteria is considered an asset. The epigenetic changes in the genome of any organism critically regulate gene expression, which in turn helps the organism to cope with environmental fluctuations. While epigenetic changes are reversible, alterations in the genomic architecture through transposable elements are irreversible and, to some extent, permanent. Permanent changes could be canalized to the next generation, so transposable elements have evolutionary significance (Pimpinelli and Piacentini, 2020).

Pioneer studies on the plant-growth-promoting properties of *M. luteus* (strains like AKAD 3-5, chp-37, MIS14, WI12, AKS4c, etc.) provide us with a novel insight into its future applicability in sustainable agriculture. Most of these studies have revealed that this species can produce indole acetic acid (IAA), gibberellic acid (GA), proline, chitinase, and HCN and that they can fix nitrogen and solubilize phosphate and potassium. The bacterium has enhanced the growth of rice, chickpea, soybean, maize, tomato, and *Arabidopsis* under treated conditions (Raza and Faisal, 2013; Dar et al., 2018; Dubey et al., 2021; Badawy et al., 2022; Chang et al., 2024; Kabiraj et al., 2024). Furthermore, some strains shielded hosts against attack from severely detrimental fungal pathogens such as *Fusarium*, suggesting its biocontrol capability (Patel et al., 2021). Some strains could alleviate drought stress in plants like *Arabidopsis*. They have a dual role in plants as they can reduce the toxicity of heavy metals and improve plant growth under metal (oid) stress (like arsenic, nickel, cadmium) (Badawy et al., 2022; Kabiraj et al., 2024).

The function of *aes* in plant–microbe interaction has been mentioned in the “Results” section. Exported IAA through the *aes* family protein could act as an interaction molecule for bacteria–plant signaling. Such proteins in endophytic bacterial strains like RIT305 could help interaction with the *Salix* plant. Strains such as AS2 and V017 do not bear this gene. We can assume, their thriving potential in particular environmental niche (AS2 was isolated from heavy metal contaminated industrial water and V017 from a cobalt isotope radiating environment) did not select this gene due to its reduced importance for their basic metabolisms. RIT305 had one extra copy of *trpB* for better and faster production of tryptophan, the precursor of IAA biosynthesis, while tryptophan has a key role in adapting to endophytic ecological niches (Khan et al., 2025). Chang et al. (2024) also demonstrated the impact of the IAA-producing strain of *M. luteus* in *Arabidopsis* growth promotion. In the context of phosphate metabolism, the concentration of phosphate within bacterial cells is critically regulated by enzymes such as transporter proteins. Generally, *pst* is expressed during phosphate starvation, ensuring selective uptake of phosphate from the environment (Kabiraj et al., 2022).

Bacterial phage genomes are one of the regulatory factors which are intricately related to bacterial evolution, stress adaptation, or often antibiotics resistance. Phage-susceptible bacterial strains are more prone to rearrange their genomic contents or acquire or delete genes, eventually leading them to thrive under a wide range of environments. It has been demonstrated that the availability of antibiotic-resistant genes in bacteria may hinder the infection of bacteriophages, leading to a lesser chance of them evolving. Both temperate and lytic phage viruses are basically involved in such interactions (Salmond and Fineran, 2015; Fernández et al., 2018). Phage-genome-containing additional genes could also alter their lifestyle and thus establish new relationships with host organisms, as observed in *S. aureus* (Xia and Wolz, 2014). Sometimes, the genome-associated ABI (phage “abortive infection”) system confers post-infection resistance that leads to the death of the bacterium after infection with bacteriophage (Li et al., 2021). To block phage-genome multiplication, several bacterial species have developed this strategy, as has been established for *M*. *luteus*. Although extensive studies have illustrated the impact of *S. aureus* upon phage infection, few have dealt with significance of pro-phages in the evolution and resilience of *M*. *luteus* to thrive under fluctuating environmental conditions.

CARD has found *vanY*, *rpsL*, and *sul* through strict heat, indicating less abundance of antibiotic resistance genes in genomes of this bacterium. Recently, Adhikary et al. (2025) found a “strict hit” putative carboxypeptidase-encoding gene (*yodJ*) from a clinical isolate. The presence of this gene helps bacteria resist the glycopeptide group of antibiotics. An opportunistic pathogenic isolate of *M*. *luteus* had *blaZ*, *ermC*, and *tetR* genes, which are responsible for resisting the beta-lactam, macrolide, and tetracycline groups of antibiotics, respectively (Shi et al., 2023). However, through comparative genomics analyses, Li et al. (2021) identified 22 antibiotic-resistant genes in this bacterial group. In these strains, *mtrA*, *murA*, *rbpA*, *strA*, etc., were preferably found. A major percentage of antibiotic-resistant genes was acquired by this bacterium through horizontal gene transfer (Li et al., 2021). Based on the critical literature survey, it can be concluded that *M*. *luteus* might be an opportunistic pathogen. Still, it is highly susceptible to antibiotics, and patients could be totally cured through antibiotic medication.

Functional annotation unveiled a core KEGG pathway and protein domain present in *M. luteus* irrespective of different ecological sources. Core KOs were highly conserved with a metabolic backbone, although many unclassified KEGG orthologs were found (Figures 7A,B). The core presence of carotenoid biosynthesis reinforces its ability to produce yellow pigment, which has significance in stress tolerance (Kandasamy and Kathirvel, 2024). In addition, the dominance of the “biosynthesis of secondary metabolites” orthologs support its cosmopolitan distribution as a secondary metabolite help to withstand several environmental stresses. In contrast, accessory and unique fractions drive niche-specific adaptation. The significant enrichment of unique KOs in xenobiotic degradation pathways and secondary metabolite biosynthesis suggests that *M. luteus* utilizes horizontal gene transfer to survive in specific environments; it also depicts its bioremediation properties (Kabiraj et al., 2025). The presence of unique KOs could be the most important feature regarding niche adaptation. Unique KOs of AS2 were 3.5 times more frequent than the very next strain (NCCP16831). The functions of unique KOs ranged from basic signaling to essential metabolisms. Here, photosynthetic orthologs were found (Figure 7D), but RAST analysis could not detect any photosynthetic genes in their core genome. Therefore, this bacterium may adopt these genes through horizontal gene transfer. A substantial proportion of core Pfam domains belongs to metabolic processes, transcriptional regulation, and structural maintenance. On the other hand, numbers of accessory Pfams were more or less the same for the strains (except AS2) and were typically associated with membrane-associated and stress-responsive proteins (Supplementary Material S4; Supplementary Table S8), suggesting their potential role in niche specialization. The accessory genome and unique Pfam content of AS2 was highest among the strains (Figure 4C). Thus, this bacterium may acquire many unique genes through HGTs, depending on its niche. However, the functions of the major percentage of domains (DUFs) have not yet been identified. It is interesting that PF09472 (most frequent in plants) has a relationship with the methane metabolism; methane is an important compound for plant metabolism. Unique KOs also identified methane-metabolizing genes in the genome of *M. luteus*. Together, this functional variability provides insights into the evolutionary strategy that enables species to persist in multiple ecological niches while maintaining essential physiological processes.

Previous research has provided insight into the adaptation strategies of this bacterium. This analysis has partially filled our knowledge gap on the impact of accessory genomes for niche adaptation. Nevertheless, there are many hypothetical proteins, several unclassified KEGG orthologs, DUF domains, and so forth. Focusing on these areas would reveal their genomic adaptation. Therefore, further *in silico* and wet-lab experiments are needed for a clearer picture. Advanced bioinformatics tools may anticipate the functions of these proteins or domains, but later validation through experimentation is necessary.

## 5 Conclusion

Using comparative genomics analyses of several strains of *M*. *luteus*, we conclude that niche-specific selection pressure is not exerted equally for each strain. In very few cases, like the presence of heavy-metal resistance genes in isolates from metal contaminated water, or extra copies of plant-growth-promoting genes in strains isolated from plant habitats, there was niche-specific gene preference. Overall, the industrial effluent isolate AS2 had the greatest number of genes in genetic islands (GIs) and occupied most of the proportion of genomes by GIs. AS2 had the largest accessory genome, and a remarkable number of metal resistance genes was also detected. The lowest attributes were found in strains like AKS4c and MT1691313. Fewer unique Pfam domains were found in strains which were isolated from plants, indicating the conserved nature of their genome.

## Data Availability

The original contributions presented in the study are included in the article/Supplementary Material; further inquiries can be directed to the corresponding author.

## References

[B1] Adedayo A. A. Babalola O. O. (2023). Genomic mechanisms of plant growth-promoting bacteria in the production of leguminous crops. Front. Genet. 14, 1276003. 10.3389/fgene.2023.1276003 38028595 PMC10654986

[B2] Adhikary R. Sarkar I. Patel D. Gang S. Nath U. K. Hazra S. (2025). Deciphering antibiotic resistance, quorum sensing, and biofilm forming genes of *Micrococcus luteus* from hemodialysis tunneled cuffed catheter tips of renal failure patients. Archives Microbiol. 207, 1–13. 10.1007/s00203-025-04310-6 40186781

[B3] Alcock B. P. Huynh W. Chalil R. Smith K. W. Raphenya A. R. Wlodarski M. A. (2023). CARD 2023: expanded curation, support for machine learning, and resistome prediction at the comprehensive antibiotic resistance Database. Nucleic Acids Research 51, D690–D699. 10.1093/nar/gkac920 36263822 PMC9825576

[B4] Arndt D. Grant J. R. Marcu A. Sajed T. Pon A. Liang Y. (2016). PHASTER: a better, faster version of the PHAST phage search tool. Nucleic Acids Research 44, W16–W21. 10.1093/nar/gkw387 27141966 PMC4987931

[B5] Arroyo-Herrera I. Román-Ponce B. Bustamante-Brito R. Guevara-Luna J. Tapia-García E. Y. Larios-Serrato V. (2023). Arsenic and chromium resistance mechanisms in the *Micrococcus luteus* group. Pedosphere 33, 600–611. 10.1016/j.pedsph.2022.07.013

[B6] Aubert C. Mandin P. Py B. (2023). Mrp and SufT, two bacterial homologs of eukaryotic CIA factors involved in Fe-S clusters biogenesis. Inorganics 11, 431. 10.3390/inorganics11110431

[B7] Aziz R. K. Bartels D. Best A. A. DeJongh M. Disz T. Edwards R. A. (2008). The RAST Server: rapid annotations using subsystems technology. BMC Genomics 9, 75. 10.1186/1471-2164-9-75 18261238 PMC2265698

[B8] Badawy I. H. Hmed A. A. Sofy M. R. Al-Mokadem A. Z. (2022). Alleviation of cadmium and nickel toxicity and phyto-stimulation of tomato plant l. by endophytic *Micrococcus luteus* and *Enterobacter cloacae* . Plants 11, 2018. 10.3390/plants11152018 35956496 PMC9370581

[B9] Barker D. F. (2022). A synergistic arrangement of two unrelated IS elements facilitates adjacent deletion in *Micrococcus luteus* ATCC49732. FEMS Microbiol. Lett. 369, fnac062. 10.1093/femsle/fnac062 35852378

[B67] Bertelli C. Laird M. R. Williams K. P. Simon Fraser University Research Computing Group, Lau B. Y. Hoad G. (2017). IslandViewer 4: expanded prediction of genomic islands for larger-scale datasets. Nucleic Acids Res. 45 (W1), W30–W35. 10.1093/nar/gkx343 28472413 PMC5570257

[B11] Chang Y.-C. Lee P.-H. Hsu C.-L. Wang W.-D. Chang Y.-L. Chuang H. (2024). Decoding the impact of a bacterial strain of *Micrococcus luteus* on arabidopsis growth and stress tolerance. Microorganisms 12, 2283. 10.3390/microorganisms12112283 39597672 PMC11596720

[B12] Chaudhry V. Patil P. B. (2016). Genomic investigation reveals evolution and lifestyle adaptation of endophytic *Staphylococcus epidermidis* . Sci. Reports 6, 19263. 10.1038/srep19263 26758912 PMC4713051

[B13] Chen X. Zhao Y. Huang S. Peñuelas J. Sardans J. Wang L. (2024). Genome-based identification of phosphate-solubilizing capacities of soil bacterial isolates. Amb. Express 14, 85. 10.1186/s13568-024-01745-w 39078439 PMC11289785

[B14] Choden P. Poolpak T. Pokethitiyook P. Yang K. M. Kruatrachue M. (2025). *In situ* bioaugmented phytoremediation of cadmium and crude oil co-contaminated soil by *Ocimum gratissimum* in association with PGPR *Micrococcus luteus* WN01. Int. J. Phytoremediation 27, 298–306. 10.1080/15226514.2024.2415535 39503056

[B15] da Costa W. L. O. Araújo C. L. de A. Dias L. M. Pereira L. C. de S. Alves J. T. C. Araújo F. A. (2018). Functional annotation of hypothetical proteins from the *Exiguobacterium antarcticum* strain B7 reveals proteins involved in adaptation to extreme environments, including high arsenic resistance. PloS One 13, e0198965. 10.1371/journal.pone.0198965 29940001 PMC6016940

[B16] Dar G. H. H. Sofi S. Padder S. A. Kabli A. (2018). Molecular characterization of rhizobacteria isolated from walnut (Juglans regia) rhizosphere in Western Himalayas and assessment of their plant growth promoting activities. Biodiversitas J. Biol. Divers. 19, 662–669. 10.13057/biodiv/d190245

[B17] Dubey A. Kumar A. Khan M. L. Payasi D. K. (2021). Plant growth-promoting and bio-control activity of *Micrococcus luteus* strain AKAD 3-5 isolated from the soybean (Glycine max (L.) Merr.) rhizosphere. Open Microbiol. J. 15, 188–197. 10.2174/1874285802115010188

[B18] D’Halluin A. Petráčková D. Čurnová I. Držmíšek J. Čapek J. Bouquet P. (2025). An IS element-driven antisense RNA attenuates the expression of serotype 2 fimbriae and the cytotoxicity of *Bordetella pertussis* . Emerg. Microbes & Infect. 14, 2451718. 10.1080/22221751.2025.2451718 39781897 PMC11774165

[B19] Earl A. M. Losick R. Kolter R. (2008). Ecology and genomics of *Bacillus subtilis* . Trends Microbiology 16, 269–275. 10.1016/j.tim.2008.03.004 18467096 PMC2819312

[B21] Fernández L. Rodríguez A. García P. (2018). Phage or foe: an insight into the impact of viral predation on microbial communities. ISME Journal 12, 1171–1179. 10.1038/s41396-018-0049-5 29371652 PMC5932045

[B22] Goswami D. Patel K. Parmar S. Vaghela H. Muley N. Dhandhukia P. (2015). Elucidating multifaceted urease producing marine Pseudomonas aeruginosa BG as a cogent PGPR and bio-control agent. Plant Growth Regulation 75, 253–263. 10.1007/s10725-014-9949-1

[B23] Hanafy R. A. Couger M. B. Baker K. Murphy C. O’Kane S. D. Budd C. (2016). Draft genome sequence of *Micrococcus luteus* strain O’Kane implicates metabolic versatility and the potential to degrade polyhydroxybutyrates. Genomics Data 9, 148–153. 10.1016/j.gdata.2016.08.006 27583205 PMC4993860

[B24] Huang X. Zeng Z. Chen Z. Tong X. Jiang J. He C. (2022). Deciphering the potential of a plant growth promoting endophyte Rhizobium sp. WYJ-E13, and functional annotation of the genes involved in the metabolic pathway. Front. Microbiol. 13, 1035167. 10.3389/fmicb.2022.1035167 36406393 PMC9671153

[B25] Kabiraj A. Biswas R. Halder U. Bandopadhyay R. (2022). Bacterial arsenic metabolism and its role in arsenic bioremediation. Curr. Microbiol. 79, 131. 10.1007/s00284-022-02810-y 35290506

[B26] Kabiraj A. Halder U. Panja A. S. Chitikineni A. Varshney R. K. Bandopadhyay R. (2023). Detailed genomic and biochemical characterization and plant growth promoting properties of an arsenic-tolerant isolate of *Bacillus pacificus* from contaminated groundwater of West Bengal, India. Biocatal. Agric. Biotechnol. 52, 102825. 10.1016/j.bcab.2023.102825

[B27] Kabiraj A. Halder U. Chitikineni A. Varshney R. K. Bandopadhyay R. (2024). Insight into the genome of an arsenic loving and plant growth-promoting strain of *Micrococcus luteus* isolated from arsenic contaminated groundwater. Environ. Sci. Pollut. Res. 31, 39063–39076. 10.1007/s11356-023-30361-7 37864703

[B28] Kabiraj A. Datta S. Bandopadhyay R. (2025). Arsenic removal from water by using bacterial dry biomasses. Natl. Acad. Sci. Lett. 48, 393–397. 10.1007/s40009-024-01467-4

[B29] Kandasamy G. D. Kathirvel P. (2024). Production, characterization and *in vitro* biological activities of crude pigment from endophytic Micrococcus luteus associated with Avicennia marina. Archives Microbiol. 206, 26. 10.1007/s00203-023-03751-1 38108901

[B30] Khan D. Shaw R. Kabiraj A. Paul A. Bandopadhyay R. (2025). Microbial inheritance through seed: a clouded area needs to be enlightened. Archives Microbiol. 207, 23. 10.1007/s00203-024-04225-8 39754662

[B31] Kozitskaya S. Cho S.-H. Dietrich K. Marre R. Naber K. Ziebuhr W. (2004). The bacterial insertion sequence element IS 256 occurs preferentially in nosocomial *Staphylococcus epidermidis* isolates: association with biofilm formation and resistance to aminoglycosides. Infect. Immunity 72, 1210–1215. 10.1128/iai.72.2.1210-1215.2004 14742578 PMC321601

[B32] Krout I. N. Scrimale T. Vorojeikina D. Boyd E. S. Rand M. D. (2022). Organomercurial lyase (MerB)-mediated demethylation decreases bacterial methylmercury resistance in the absence of mercuric reductase (MerA). Appl. Environ. Microbiol. 88, e00010-22. 10.1128/aem.00010-22 35138926 PMC8939331

[B33] Lee S. An Y.-W. Choi C.-H. Yun M.-R. Kim S. Cheong H. (2020). Complete genome sequences of *Micrococcus luteus* strains NCCP 15687 and NCCP 16831, isolated in South Korea. Microbiol. Resource Announcements 9, 10–1128. 10.1128/MRA.01558-19 32107302 PMC7046823

[B34] Li Y. Sun Z.-Z. Rong J.-C. Xie B.-B. (2021). Comparative genomics reveals broad genetic diversity, extensive recombination and nascent ecological adaptation in *Micrococcus luteus* . BMC Genomics 22, 124. 10.1186/s12864-021-07432-5 33602135 PMC7890812

[B35] Liu H. Zhang Y. Wang Y. Xie X. Shi Q. (2021). The connection between Czc and Cad systems involved in cadmium resistance in *Pseudomonas putida* . Int. Journal Molecular Sciences 22, 9697. 10.3390/ijms22189697 34575861 PMC8469834

[B36] Majhi K. Let M. Halder U. Chitikineni A. Varshney R. K. Bandopadhyay R. (2023). Copper removal capability and genomic insight into the lifestyle of copper mine inhabiting *Micrococcus yunnanensis* GKSM13. Environ. Research 223, 115431. 10.1016/j.envres.2023.115431 36754109

[B37] Neal-McKinney J. M. Konkel M. E. (2012). The Campylobacter jejuni CiaC virulence protein is secreted from the flagellum and delivered to the cytosol of host cells. Front. Cellular Infection Microbiology 2, 31. 10.3389/fcimb.2012.00031 22919623 PMC3417660

[B38] Norambuena J. Miller M. Boyd J. M. Barkay T. (2020). Expression and regulation of the mer operon in *Thermus thermophilus* . Environ. Microbiology 22, 1619–1634. 10.1111/1462-2920.14953 32090420

[B39] Omeershffudin U. N. M. Kumar S. (2019). *In silico* approach for mining of potential drug targets from hypothetical proteins of bacterial proteome. Int. J. Mol. Biol. Open Access 4, 145–152. 10.15406/ijmboa.2019.04.00111

[B40] Ozer E. A. (2018). ClustAGE: a tool for clustering and distribution analysis of bacterial accessory genomic elements. BMC Bioinformatics 19, 150. 10.1186/s12859-018-2154-x 29678129 PMC5910555

[B41] Partila A. M. El-Bialy H. A. A. Gomaa O. M. (2025). Mineral recovery by bioprecipitation from desalination brine using irradiated *Micrococcus luteus* . Int. J. Environ. Sci. Technol. 22, 1–14. 10.1007/s13762-025-06439-9

[B42] Patel P. Patel K. Dhandhukia P. Thakker J. N. (2021). Plant growth promoting traits of marine *Micrococcus* sp. with bio-control ability against Fusarium in chickpea plant. Vegetos 34, 94–101. 10.1007/s42535-021-00191-4

[B43] Pathak A. Chauhan A. Ewida A. Y. I. Stothard P. (2016). Whole genome sequence analysis of an Alachlor and Endosulfan degrading *Micrococcus* sp. strain 2385 isolated from Ochlockonee River, Florida. J. Genomics 4, 42–47. 10.7150/jgen.16156 27672405 PMC5033731

[B44] Pimpinelli S. Piacentini L. (2020). Environmental change and the evolution of genomes: transposable elements as translators of phenotypic plasticity into genotypic variability. Funct. Ecol. 34, 428–441. 10.1111/1365-2435.13497

[B45] Raza F. A. Faisal M. (2013). Growth promotion of maize by desiccation tolerant’*Micrococcus luteus’*-cp37-chp37 isolated from Cholistan desert, Pakistan. Aust. J. Crop Sci. 7, 1693–1698.

[B46] Richter M. Rosselló-Móra R. Oliver Glöckner F. Peplies J. (2016). JSpeciesWS: a web server for prokaryotic species circumscription based on pairwise genome comparison. Bioinformatics 32, 929–931. 10.1093/bioinformatics/btv681 26576653 PMC5939971

[B48] Salmond G. P. C. Fineran P. C. (2015). A century of the phage: past, present and future. Nat. Rev. Microbiol. 13, 777–786. 10.1038/nrmicro3564 26548913

[B49] Sher S. Hussain S. Z. Rehman A. (2020). Phenotypic and genomic analysis of multiple heavy metal–resistant *Micrococcus luteus* strain AS2 isolated from industrial waste water and its potential use in arsenic bioremediation. Appl. Microbiology Biotechnology 104, 2243–2254. 10.1007/s00253-020-10351-2 31927763

[B50] Shi X. Qiu S. Ji L. Lu H. Wu S. Chen Q. (2023). Pathogenetic characterization of a *Micrococcus luteus* strain isolated from an infant. Front. Pediatr. 11, 1303040. 10.3389/fped.2023.1303040 38188910 PMC10770869

[B51] Siguier P. Gourbeyre E. Chandler M. (2014). Bacterial insertion sequences: their genomic impact and diversity. FEMS Microbiology Reviews 38, 865–891. 10.1111/1574-6976.12067 24499397 PMC7190074

[B53] Smaldone G. T. Helmann J. D. (2007). CsoR regulates the copper efflux operon copZA in *Bacillus subtilis* . Microbiology 153, 4123–4128. 10.1099/mic.0.2007/011742-0 18048925 PMC3019219

[B54] Soren K. Khan D. Kabiraj A. Halder U. Let M. Chitikineni A. (2025). Physiological and genomic insights into *Bacillus* sp. BRTN from Baratang mud volcano with emphasis on SUF system proteins. Archives Microbiology 207, 1–22. 10.1007/s00203-025-04480-3 40996543

[B57] Tang T. Li R. Li H. Feng H. (2025). Adaptive laboratory evolution of *Micrococcus luteus* and identification of genes associated with radioresistance through genome-wide association study. Sci. Rep. 15, 5614. 10.1038/s41598-025-90434-0 39955430 PMC11830106

[B58] Tempel S. Bedo J. Talla E. (2022). From a large-scale genomic analysis of insertion sequences to insights into their regulatory roles in prokaryotes. BMC Genomics 23, 451. 10.1186/s12864-022-08678-3 35725380 PMC9208149

[B60] Vaishnav V. K. Chatterjee T. Agrawal M. (2024). Identification and determination of mic value of heavy metal tolerance bacteria from coal mine of chhattisgarh and their molecular characterization. J. Nonlinear Anal. Optim. 15 (2), 167–174.

[B61] Vigil-Stenman T. Ininbergs K. Bergman B. Ekman M. (2017). High abundance and expression of transposases in bacteria from the Baltic Sea. ISME Journal 11, 2611–2623. 10.1038/ismej.2017.114 28731472 PMC5649170

[B62] Vijay V. Vandana K. B. Mathan Kumar R. Prabagaran S. R. (2017). Genetic analysis of arsenic metabolism in *Micrococcus luteus* BPB1, isolated from the Bengal basin. Ann. Microbiol. 67, 79–89. 10.1007/s13213-016-1239-x

[B63] Vincent A. T. (2024). Bacterial hypothetical proteins may be of functional interest. Front. Bacteriol. 3, 1334712. 10.3389/fbrio.2024.1334712

[B64] Xia G. Wolz C. (2014). Phages of *Staphylococcus aureus* and their impact on host evolution. Infect. Genet. Evol. 21, 593–601. 10.1016/j.meegid.2013.04.022 23660485

[B65] Young M. Artsatbanov V. Beller H. R. Chandra G. Chater K. F. Dover L. G. (2010). Genome sequence of the Fleming strain of *Micrococcus luteus*, a simple free-living actinobacterium. J. Bacteriology 192, 841–860. 10.1128/JB.01254-09 19948807 PMC2812450

[B66] Zhu M. Zhu Q. Yang Z. Liang Z. (2021). Clinical characteristics of patients with *Micrococcus luteus* bloodstream infection in a Chinese tertiary-care hospital. Pol. Journal Microbiology 70, 321–326. 10.33073/pjm-2021-030 34584526 PMC8459002

