# Supplementary material for: Unveiling the genomic secrets of Micrococcus luteus through a comprehensive comparative genomics approach
Source: Front Genet. 2026 May 7;17:1778782. doi: 10.3389/fgene.2026.1778782 (PMC13189705; doi:10.3389/fgene.2026.1778782)
Supplement: Supplementary file 2 [file Supplementaryfile3.docx]

***Supplementary Material***

**Unveiling the genomic secrets of *Micrococcus luteus* through comprehensive comparative genomics approach**


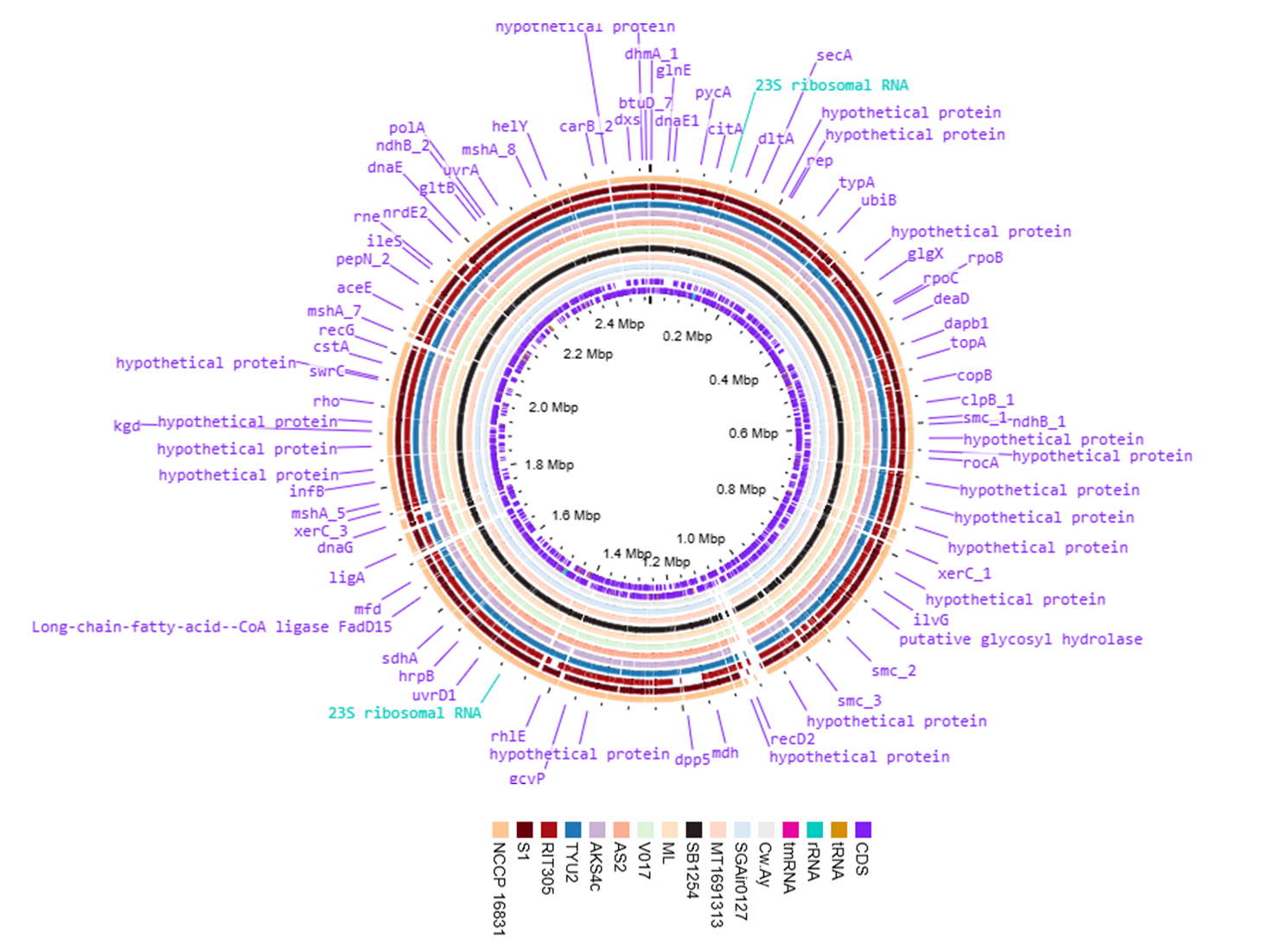


**Figure S1.** Comparative circular view of the genomes. Each circle is indicating one genome sequence as depicted by their respective coloration. Type strain NCCP 2665 was considered as control genome. Genes (represented by violet color outside the circles) were annotated from the type strain. The empty parts of the circles are indicating deleted parts of genomes.


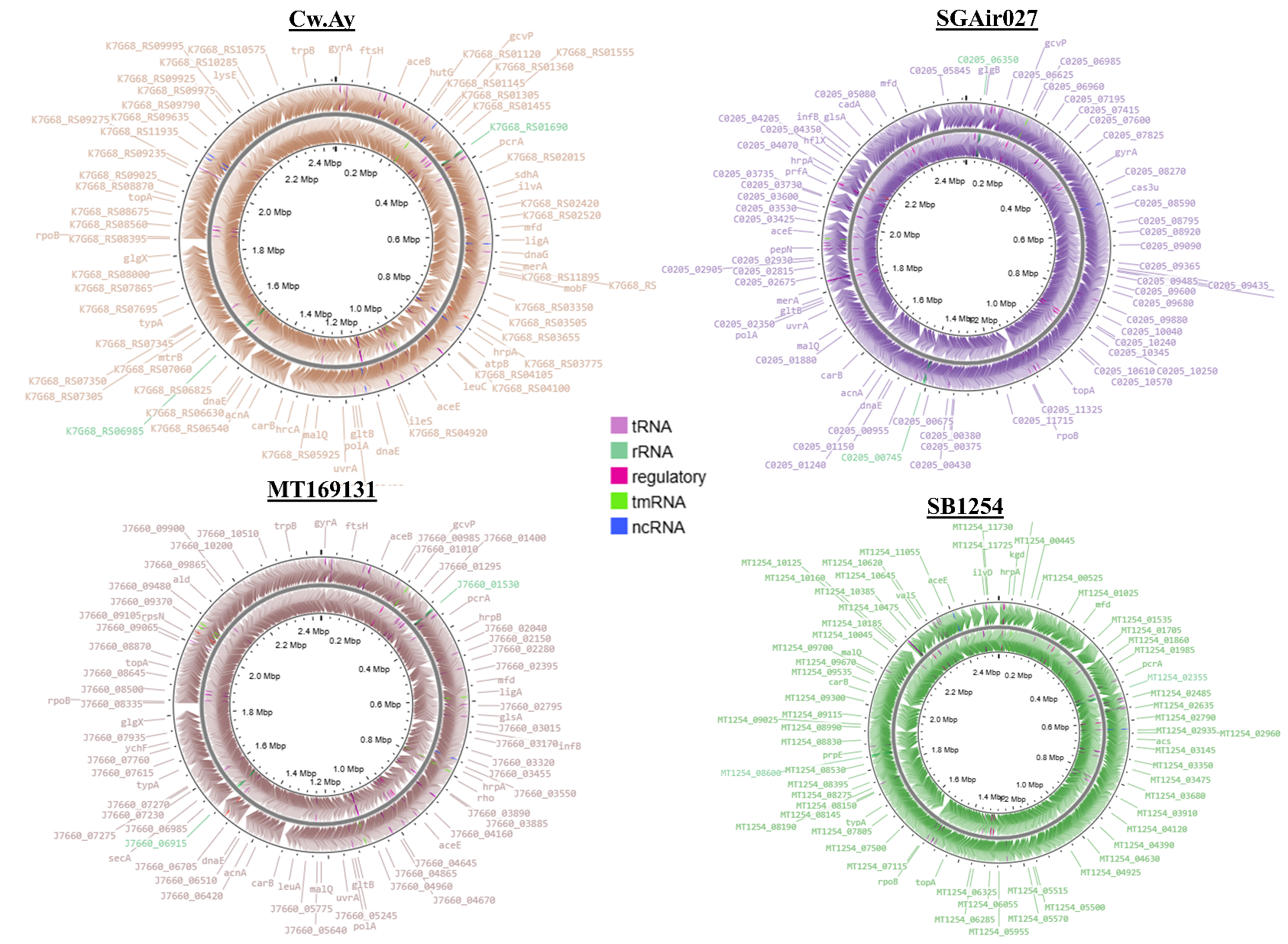


D

C

B

A

**Figure S2.** Circular view of the genomes with annotated genes. In addition to the protein coding sequences, tRNA, rRNA, ncRNA, etc. were also annotated. These FIGUREs demonstrated partially about genetic differences across strains. (A) and (B). were isolated from air, while (C) and (D) were isolated from saline water.

**
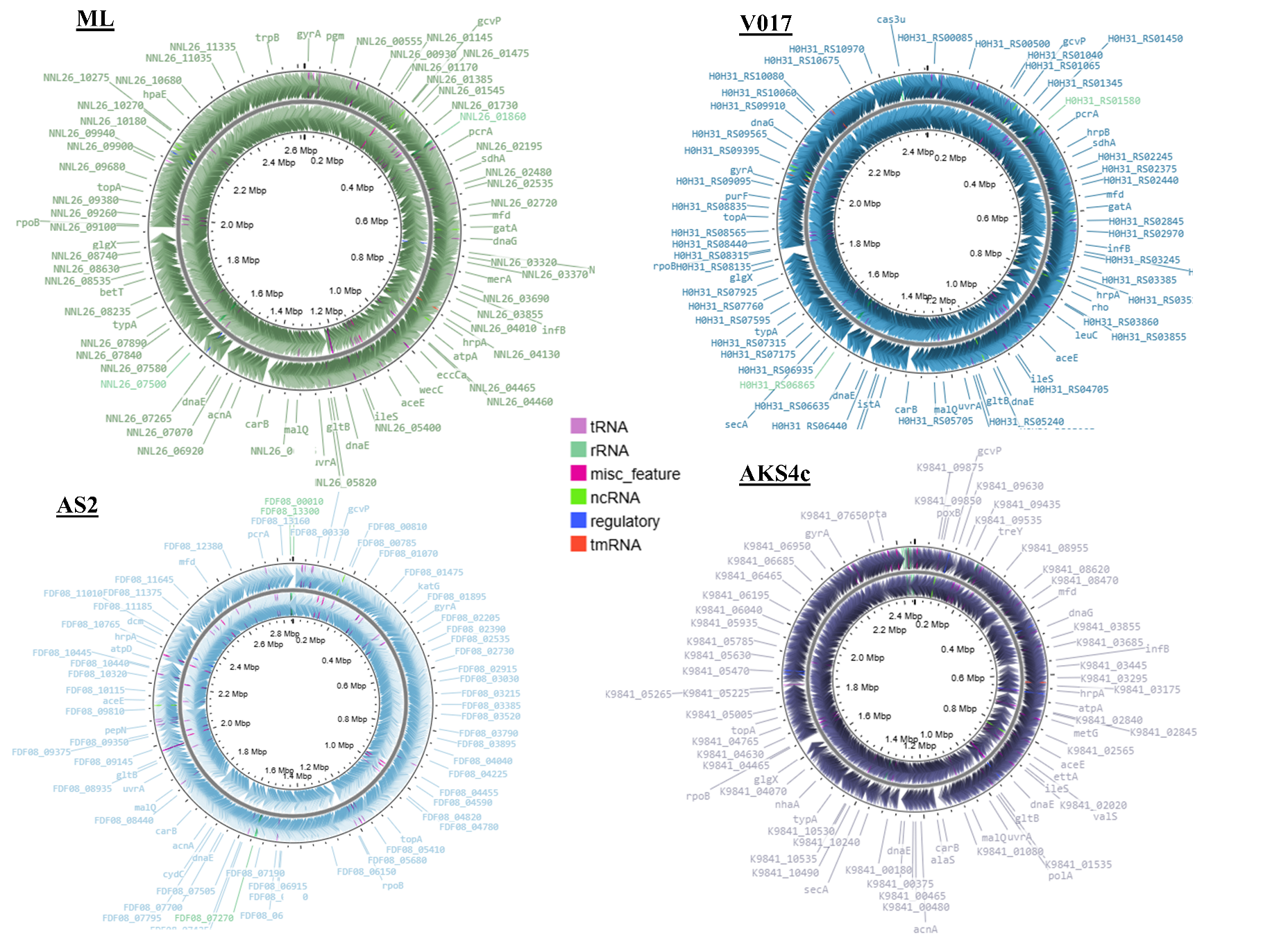
**

C

D

A

B

**Figure S3.** Circular view of the genomes with annotated genes. In addition to the protein coding sequences, tRNA, rRNA, ncRNA, etc. were also annotated. These FIGUREs demonstrated partially about genetic differences across strains. (A) and (B) were isolated from soil, while (C) and (D) were isolated from metal contaminated water.

**
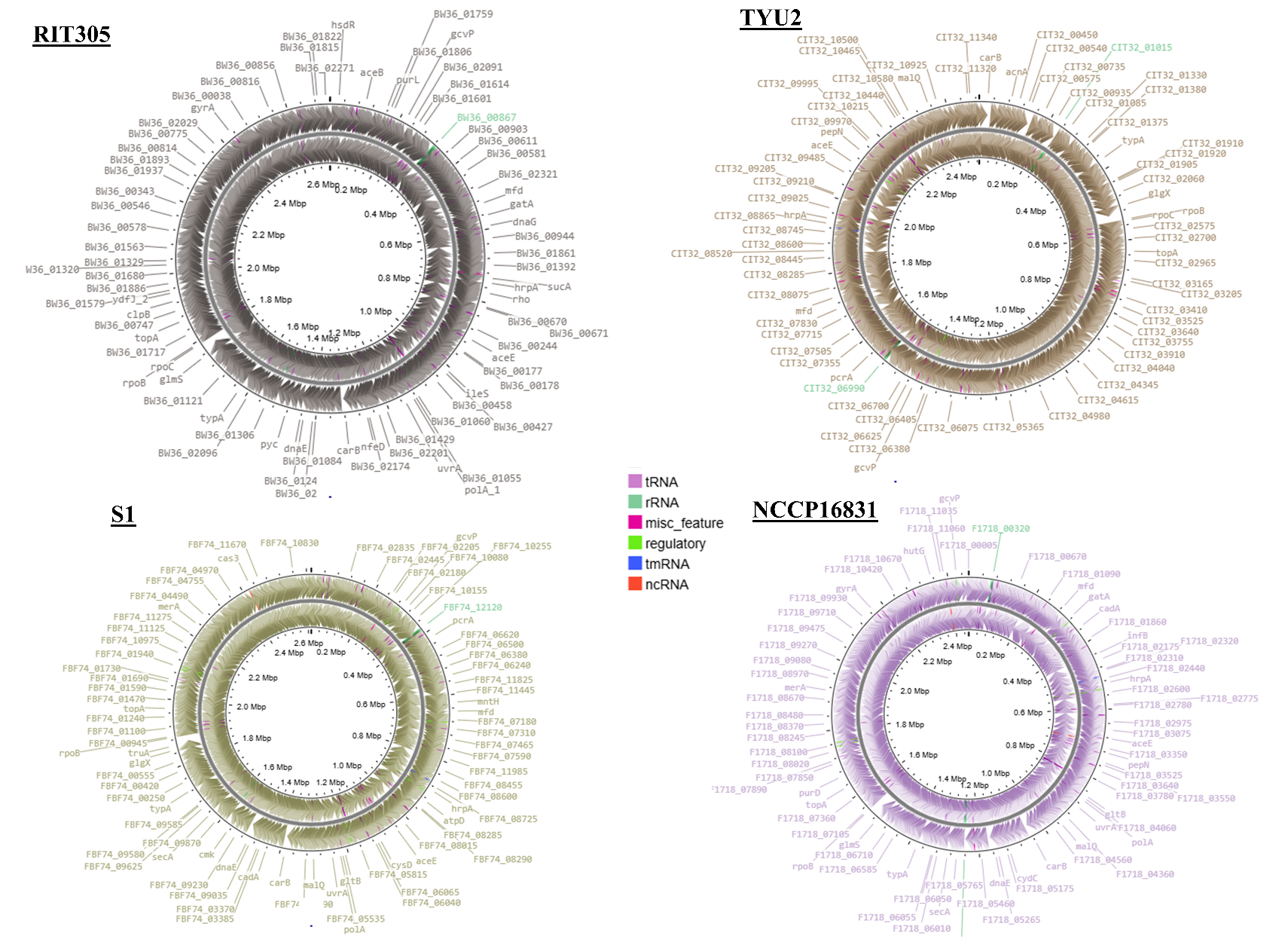
**

A

B

D

C

**Figure S4.** Circular view of the genomes with annotated genes. In addition to the protein coding sequences, tRNA, rRNA, ncRNA, etc. were also annotated. These FIGUREs demonstrated partially about genetic differences across strains. (A) and (B)were isolated from plant, while (C) and (D) were isolated from vertebrates.


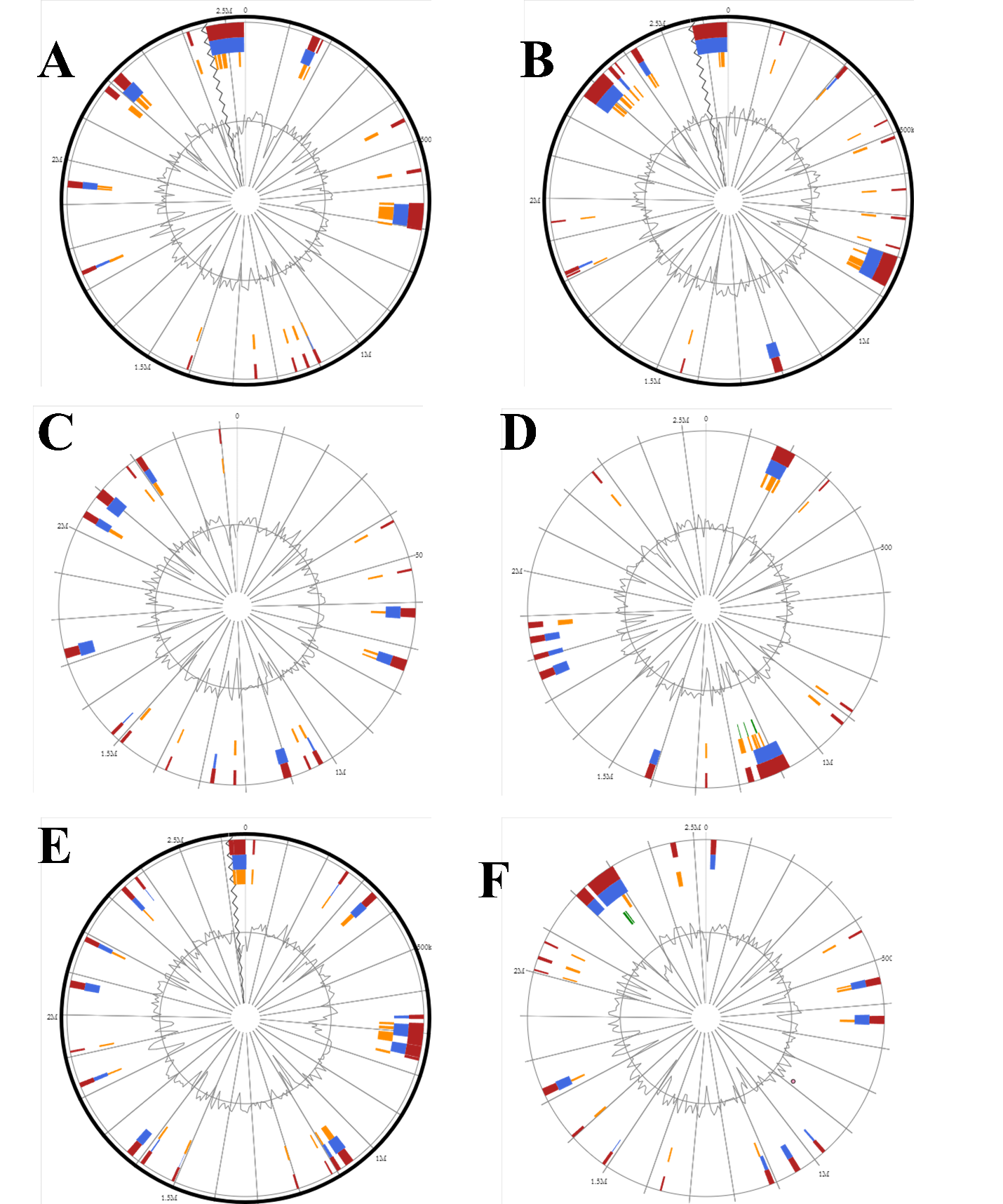


**Figure S5.** Genomic islands of selected bacterial strains, predicted by IslandViewer 4. The figures are representing circular genome of strains and probable position of genomic islands within them. Alphabets are indicating strains names like (A) CW.Ay, (B) SGAir0127, (C) MT1691313, (D) SB1254, (E) ML, and (F) V017.


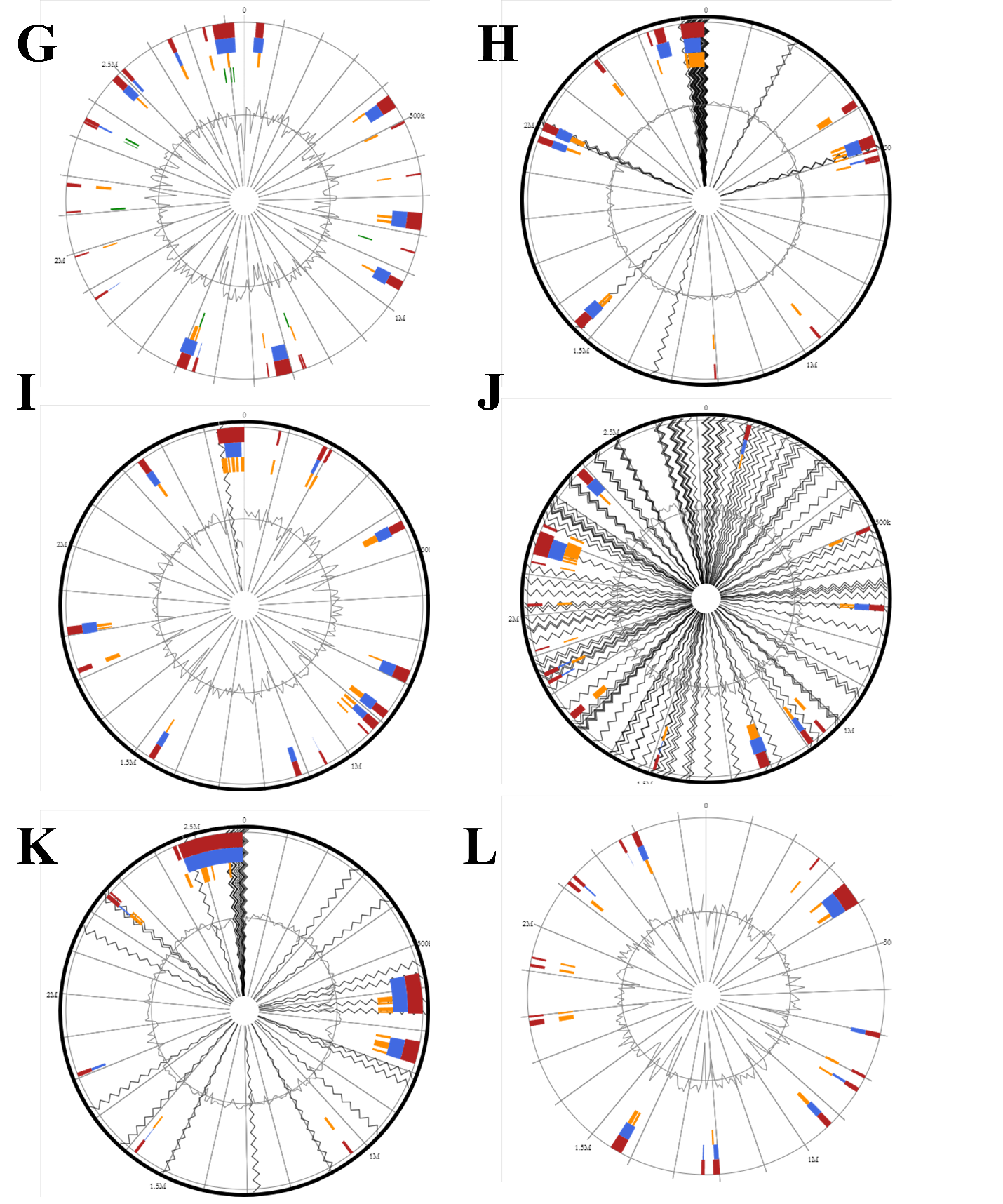


**Figure S6.** Genomic islands of selected bacterial strains, predicted by IslandViewer 4. The figures are representing circular genome of strains and probable position of genomic islands within them. Alphabets are indicating strains names like (G) AS2, (H) AKS4c, (I) TYU2, (J) RIT305, (K) S1, (L) NCCP16831

A


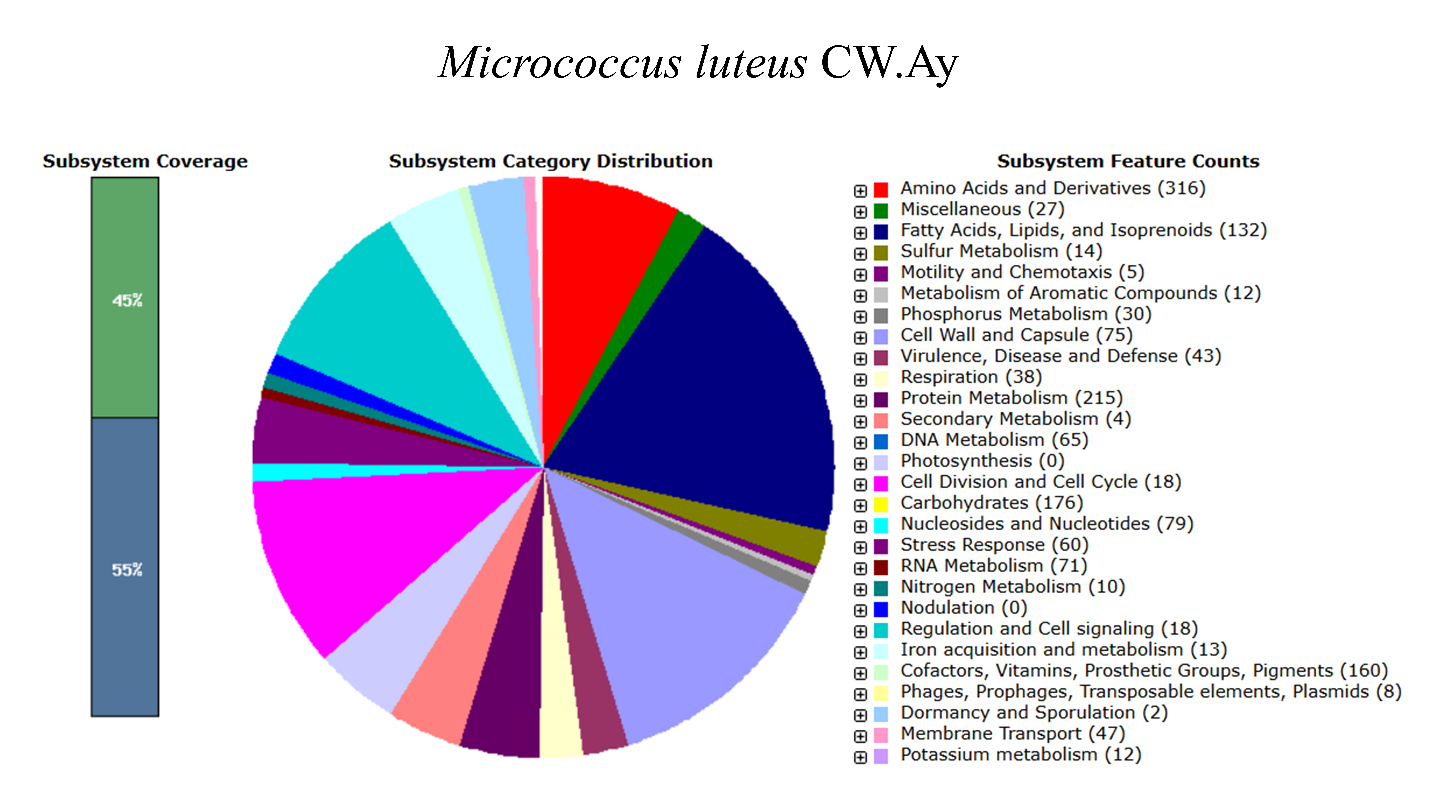


B


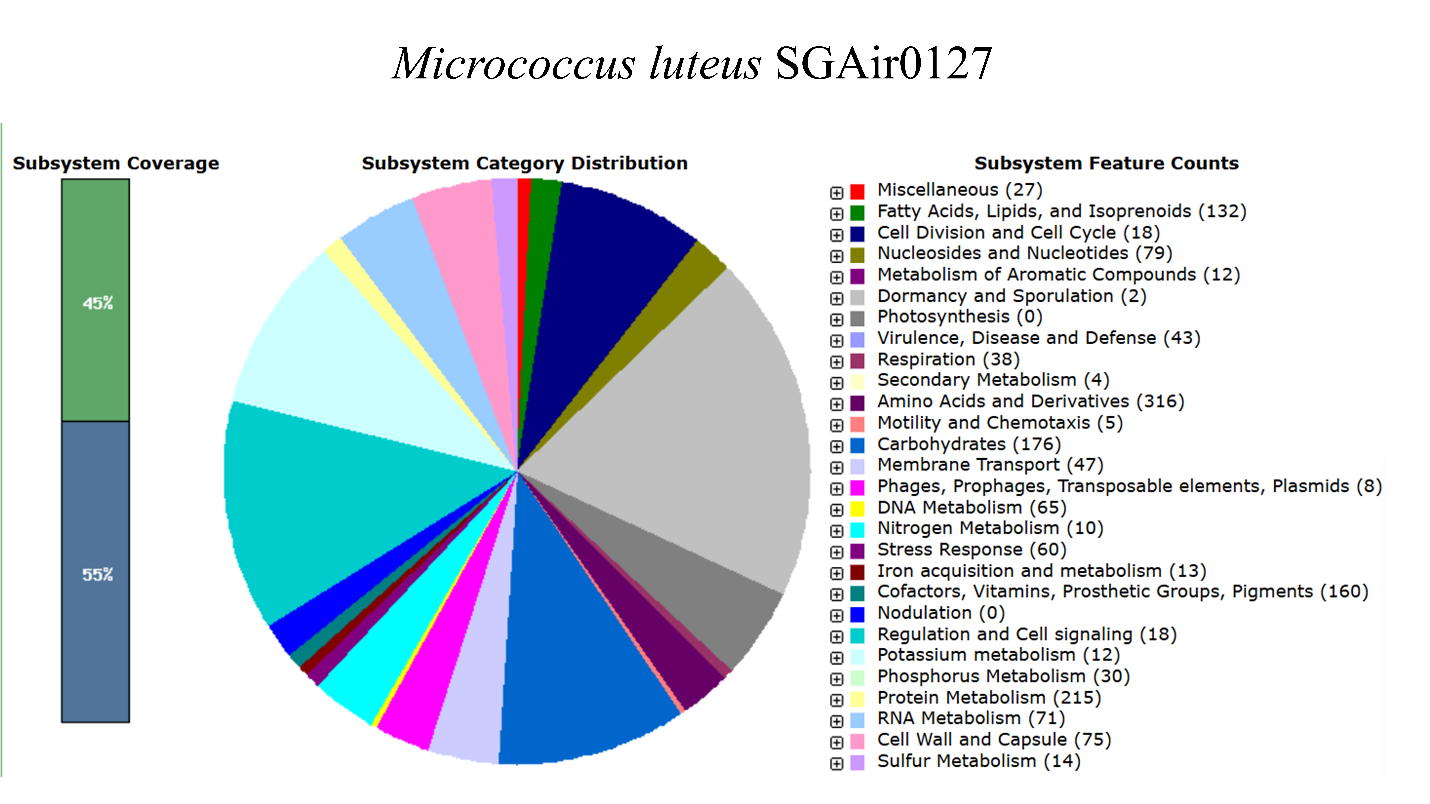
 **Figure S7.** RAST analyses of two strains isolated from air (A and B). The figures are demonstrating subsystem coverage, their distribution categories, and subsystem feature counts. Number of genes are provided within brackets.

A

B
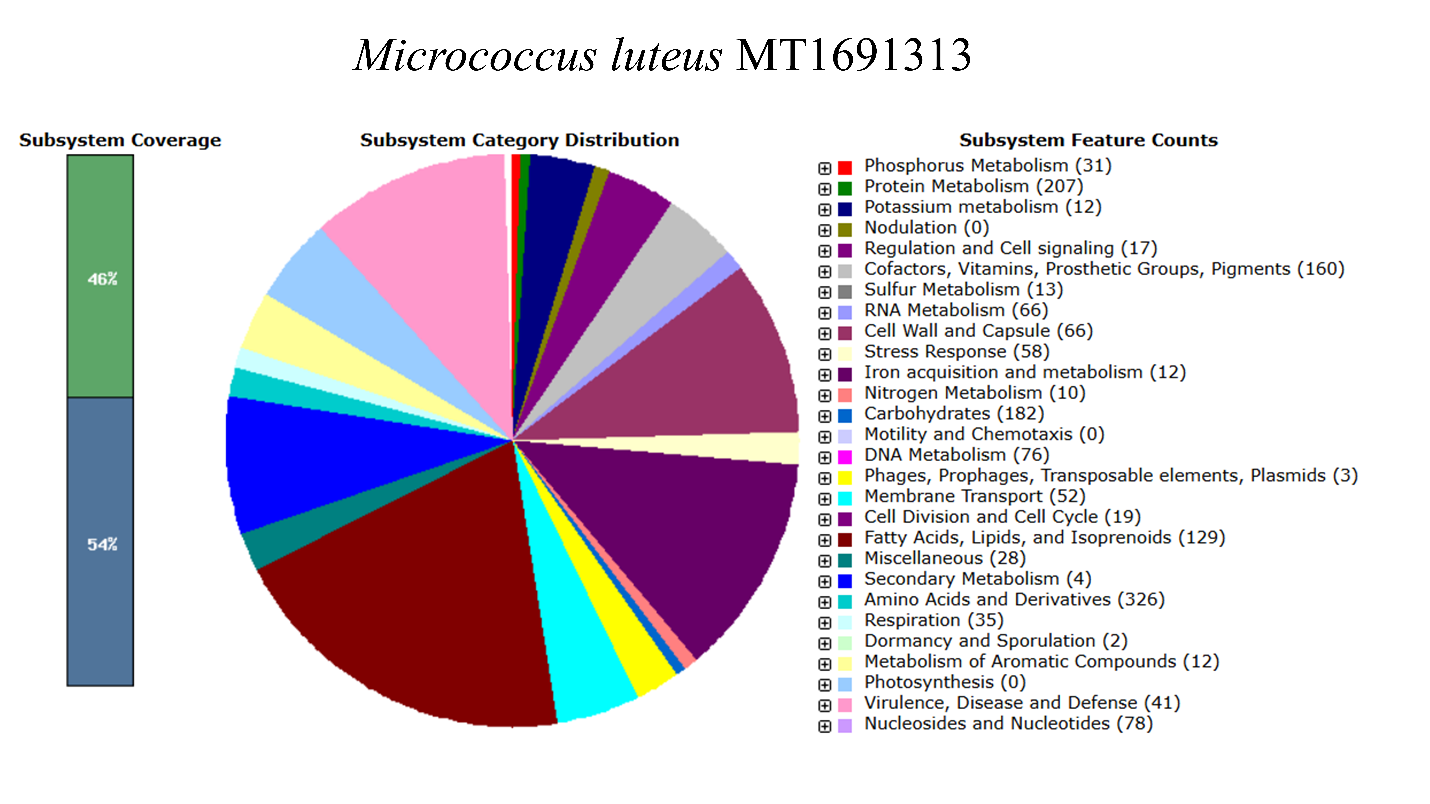


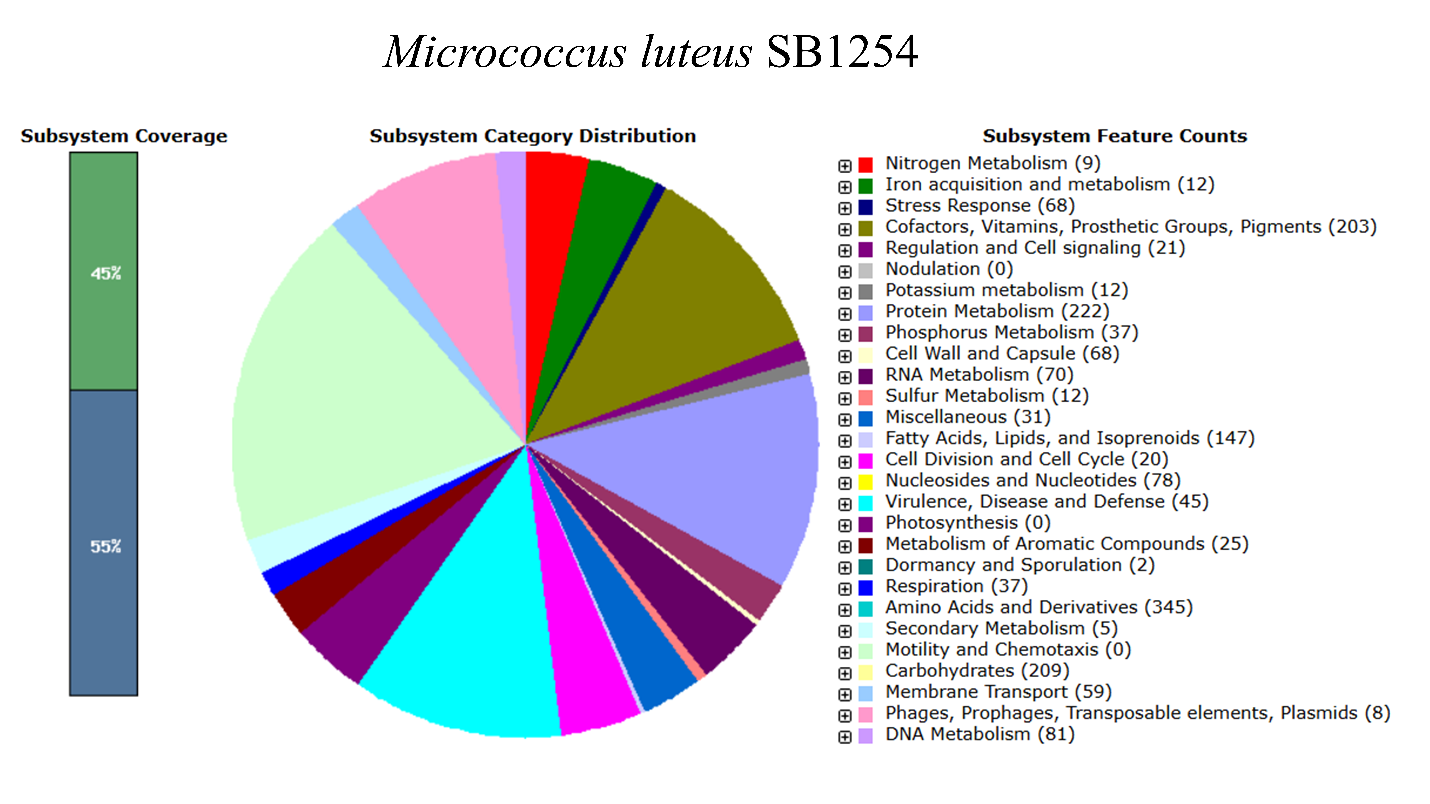


**Figure S8.** RAST analyses of two strains isolated from saline water (A and B). The figures are demonstrating subsystem coverage, their distribution categories, and subsystem feature counts. Number of genes are provided within brackets.

A


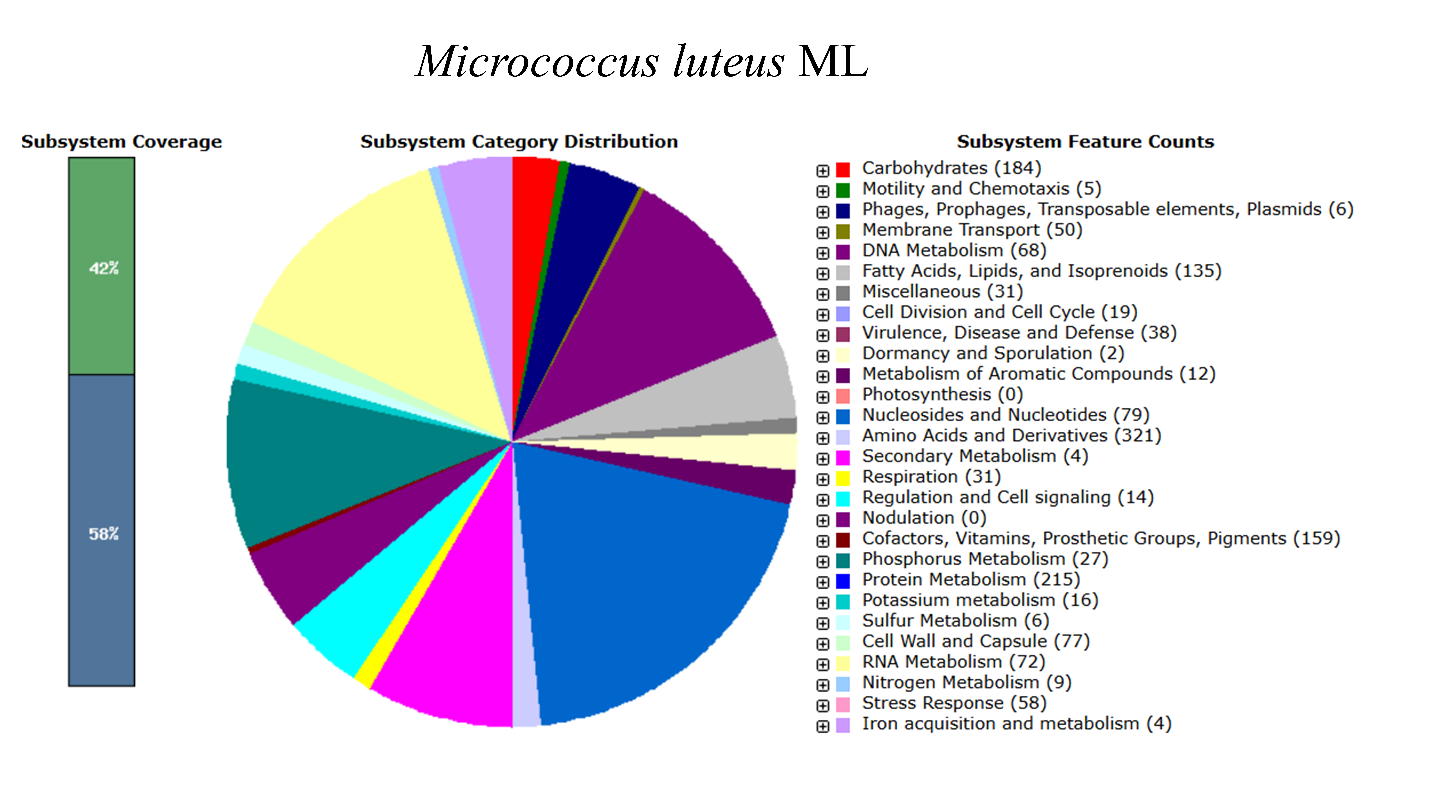


B


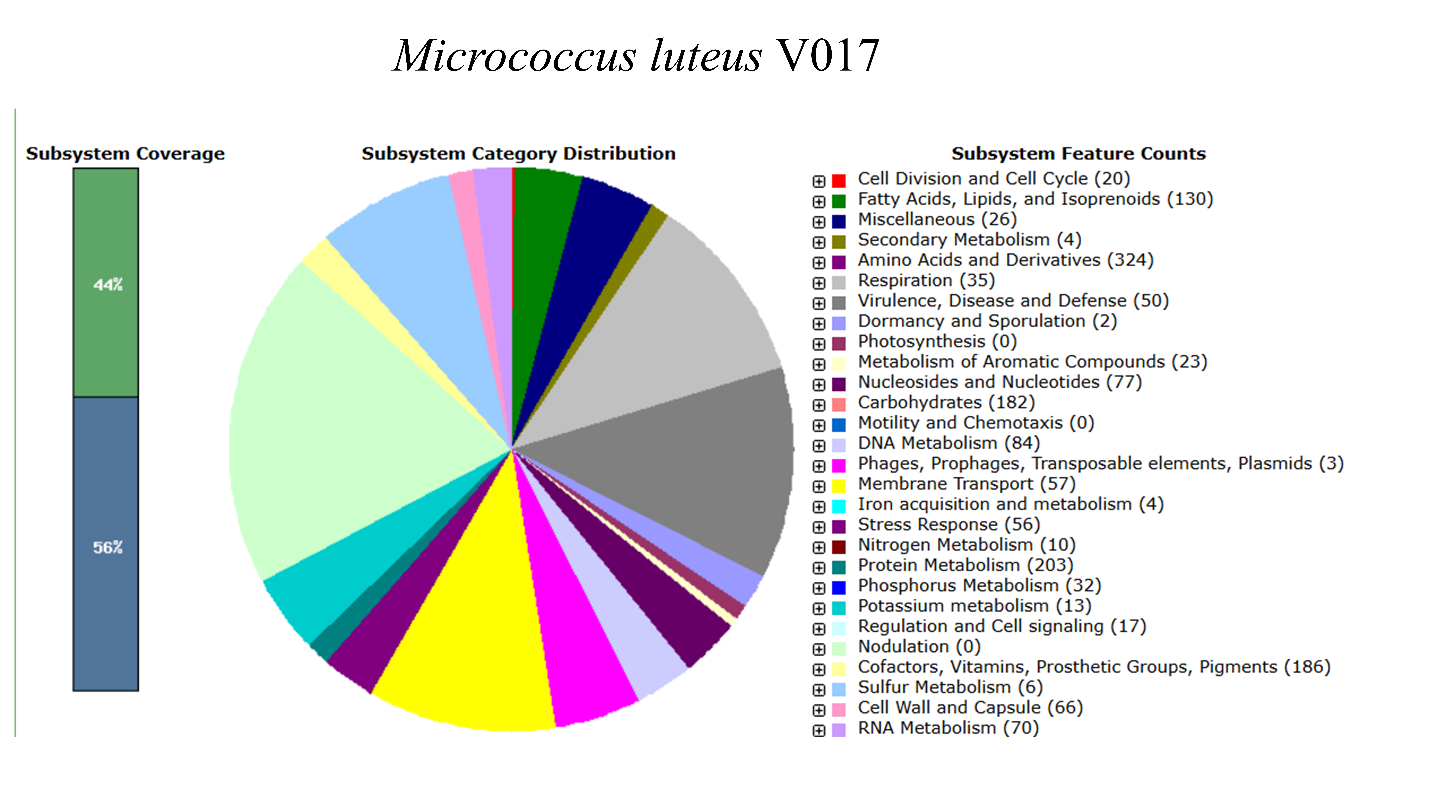


**Figure S9.** RAST analyses of two strains isolated from soil (A and B). The figures are demonstrating subsystem coverage, their distribution categories, and subsystem feature counts. Number of genes are provided within brackets.

A


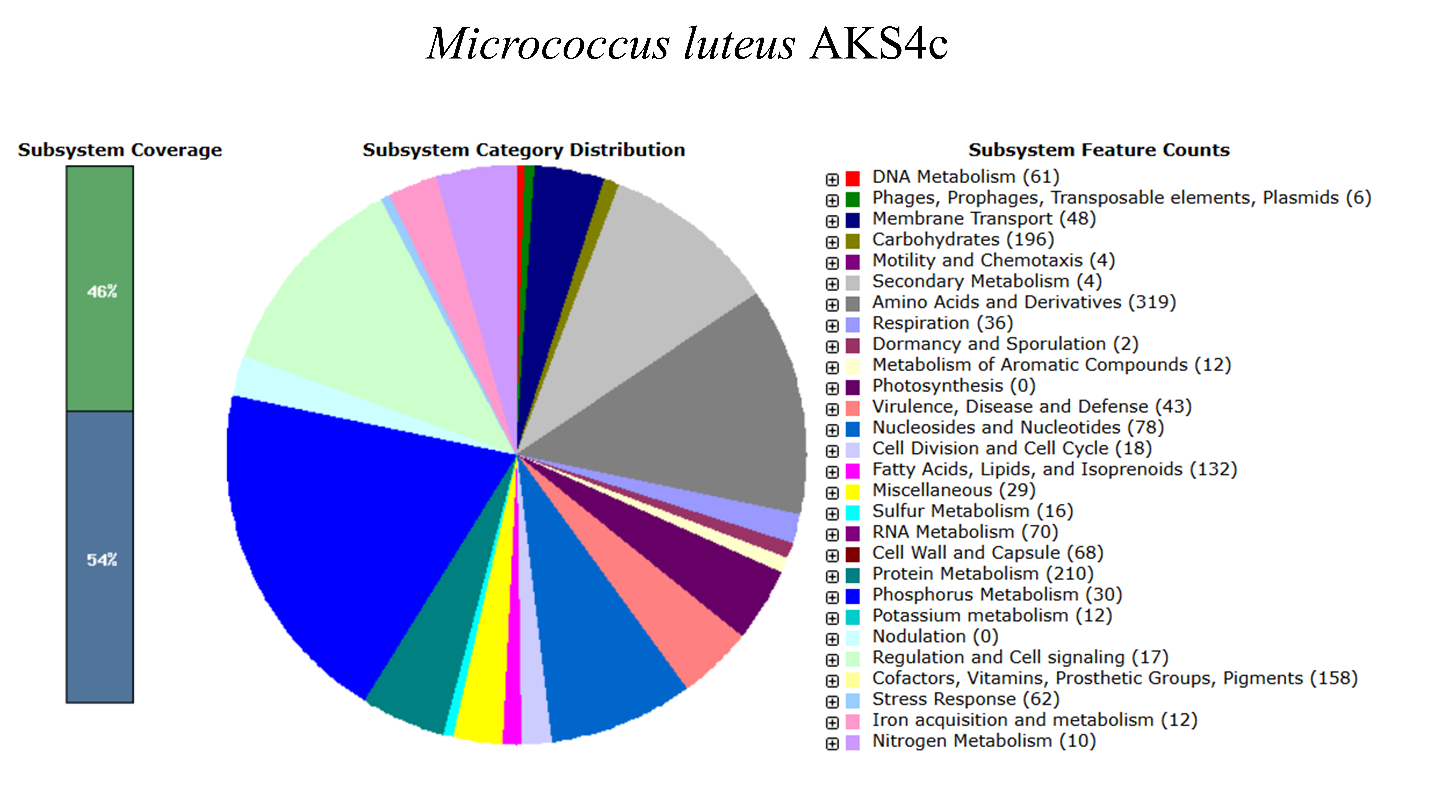

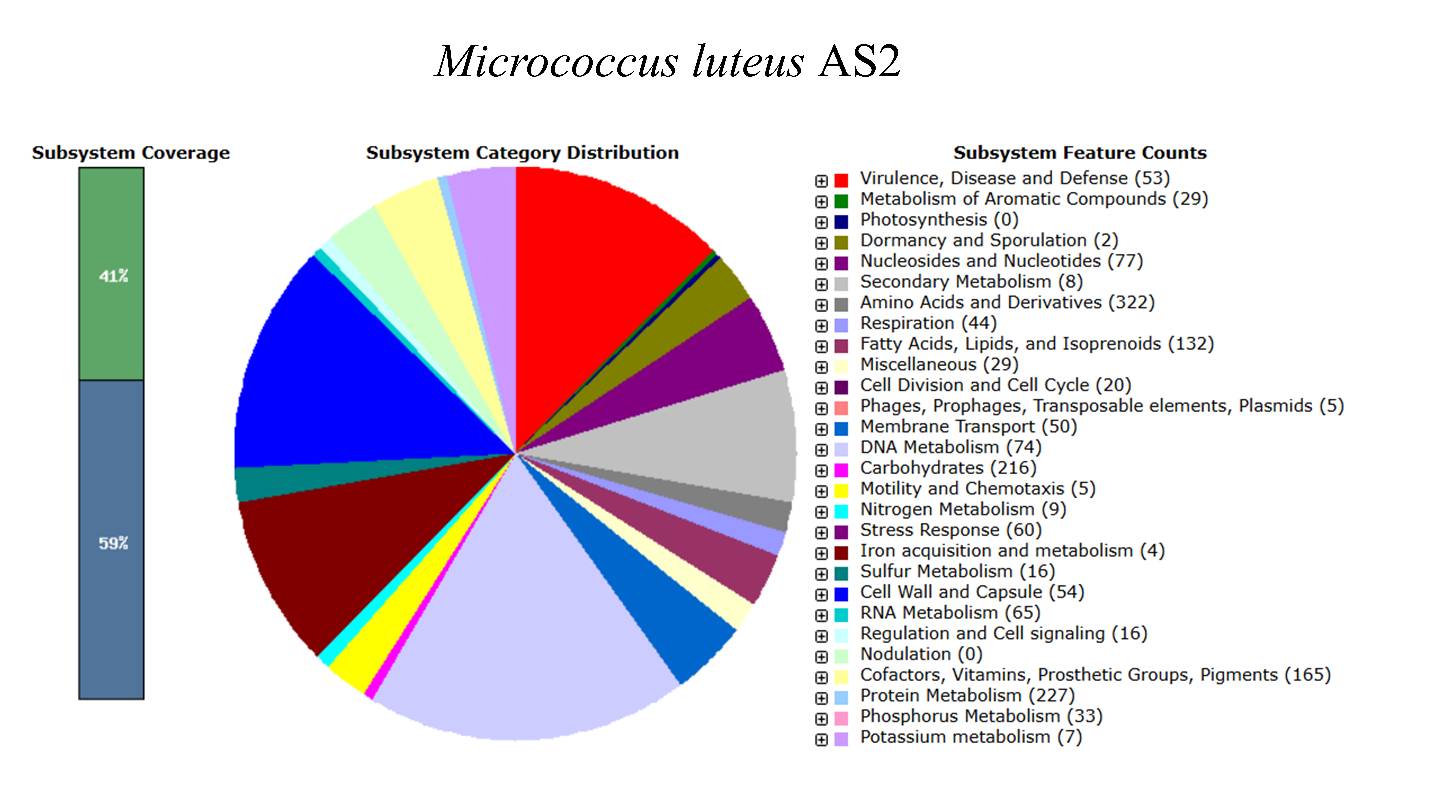


B

**Figure S10.** RAST analyses of two strains isolated from contaminated water. (A and B) The figures are demonstrating subsystem coverage, their distribution categories, and subsystem feature counts. Number of genes are provided within brackets.

A
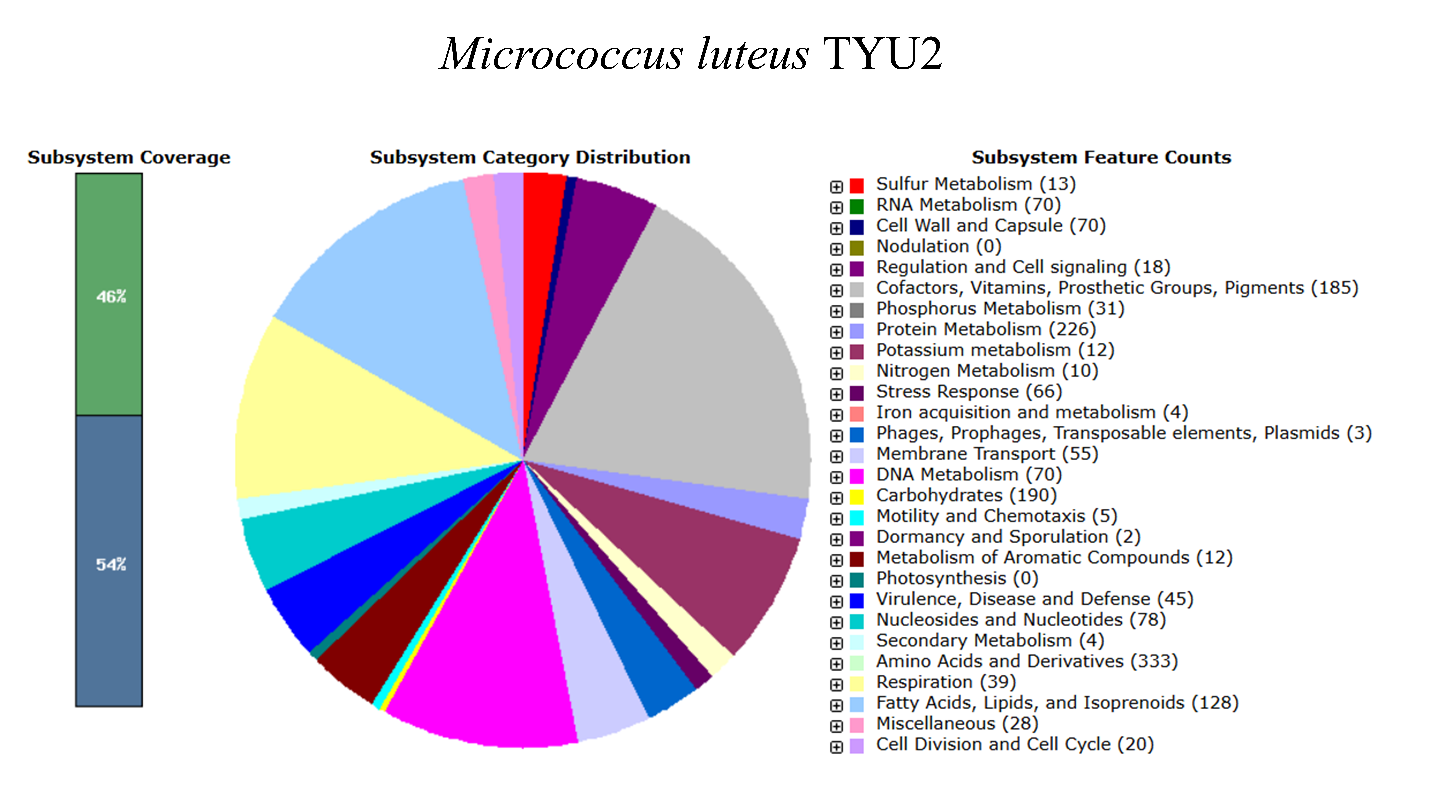


B


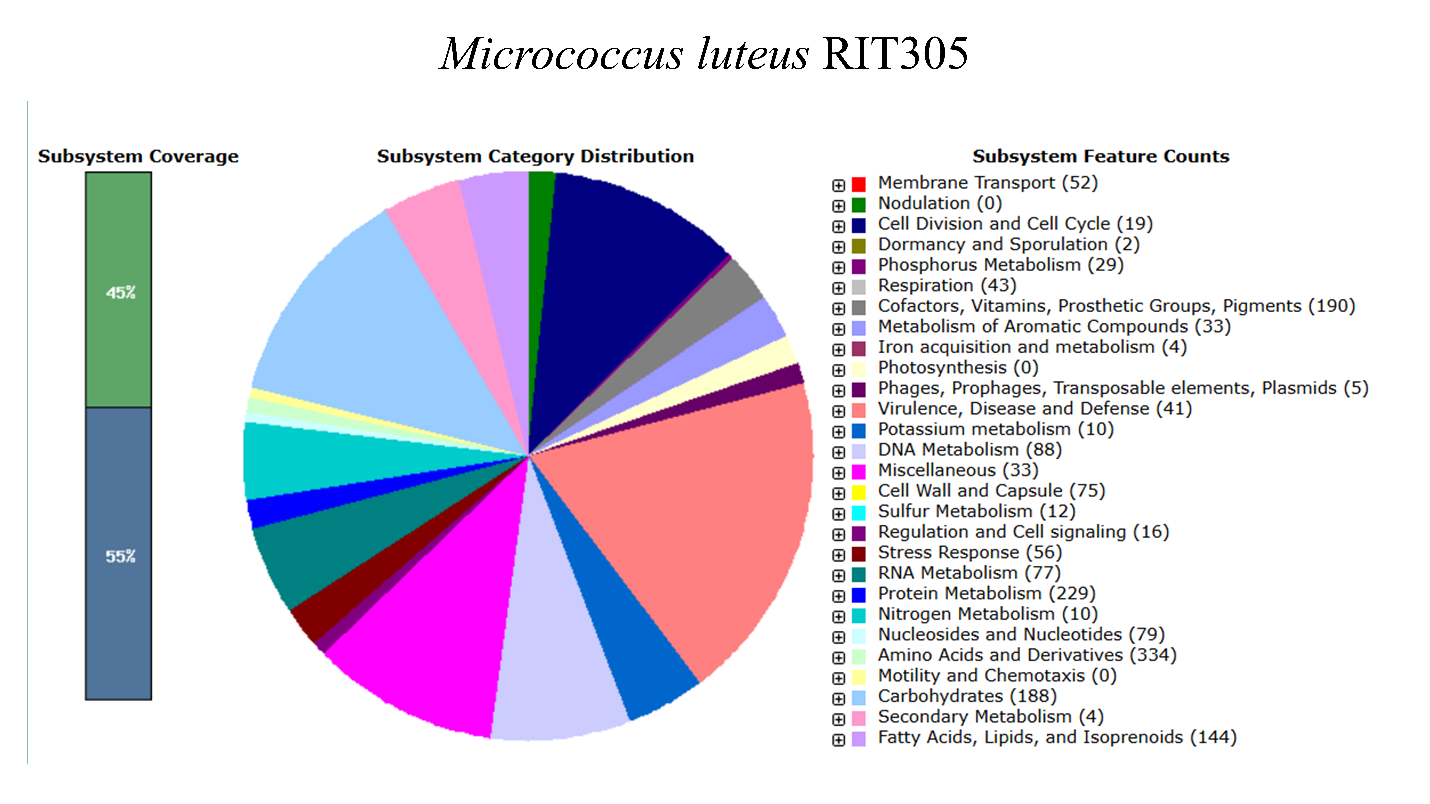


**Figure S11.** RAST analyses of two strains isolated from contaminated water. (A and B) The figures are demonstrating subsystem coverage, their distribution categories, and subsystem feature counts. Number of genes are provided within brackets.


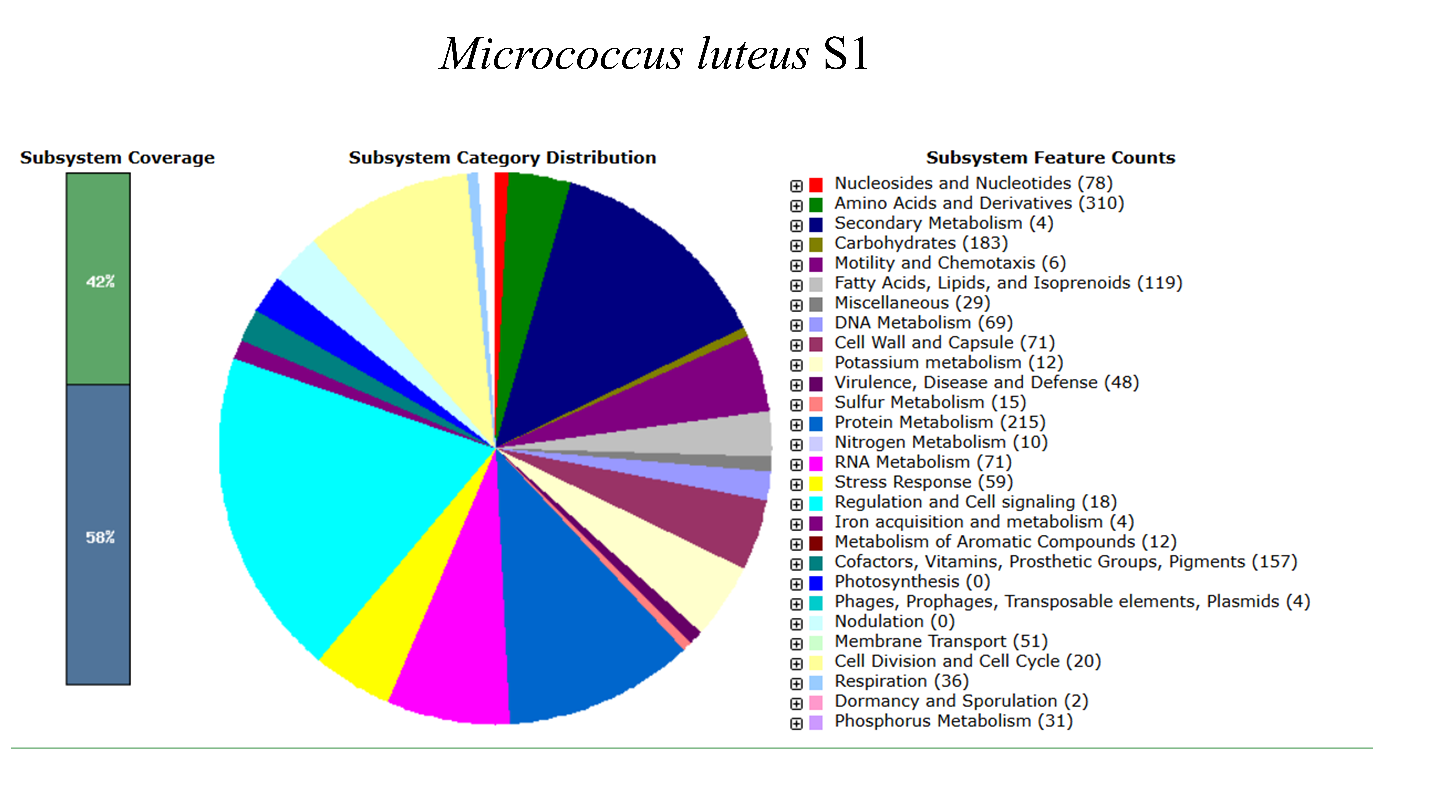
B


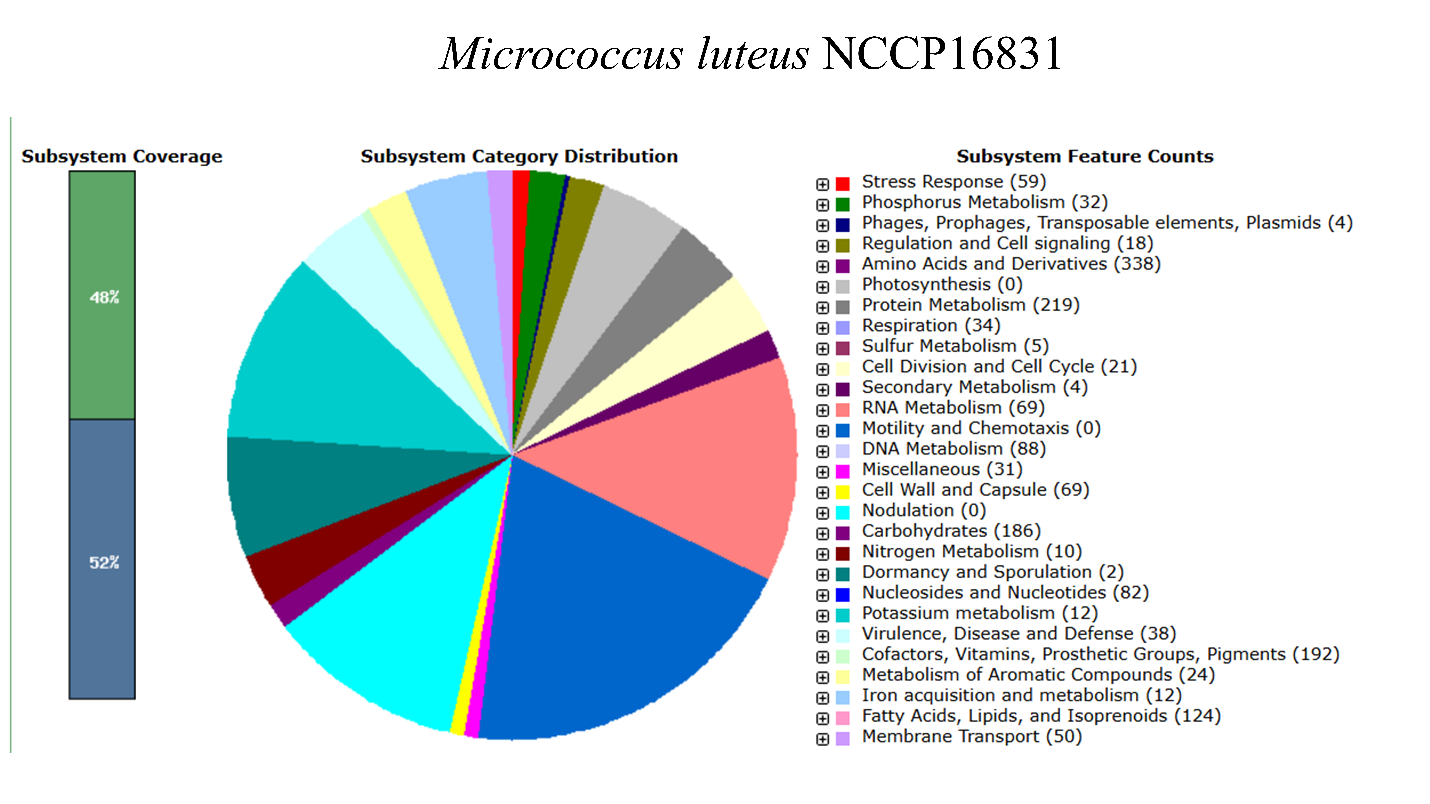


**Figure S12.** RAST analyses of two strains isolated from vertebrates (A and B). The figures are demonstrating subsystem coverage, their distribution categories, and subsystem feature counts. Number of genes are provided within brackets.


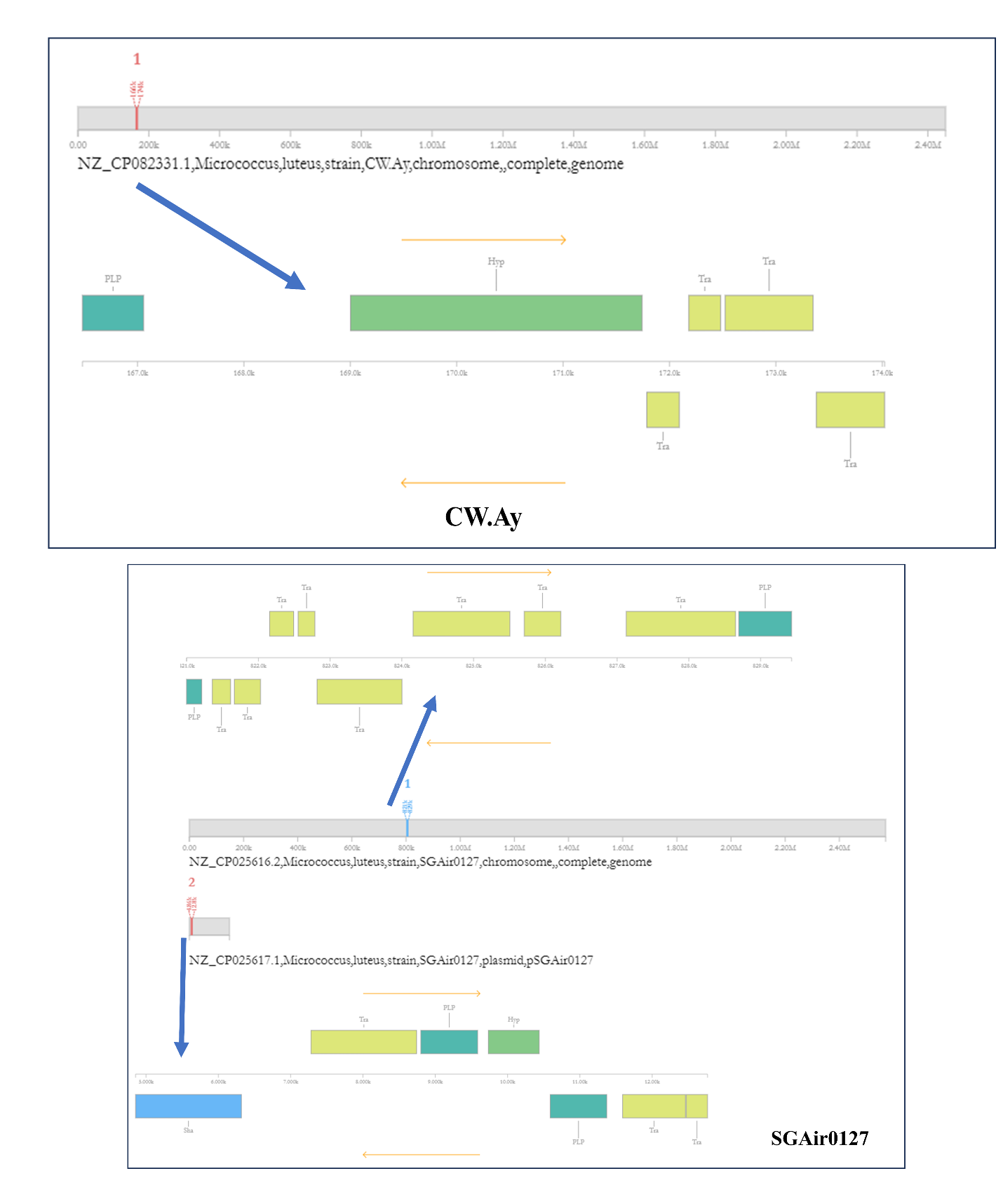


**Figure S13.** Prophages in the bacterial genomes: phage genomic content of the strains was studied using PHASTER tool. Bacteriophage harboring genes along with their predicted positions within the bacterial genome are represented here. Arrows are indicating the predicted genes within phage genomes. These strains were isolated from **air.**


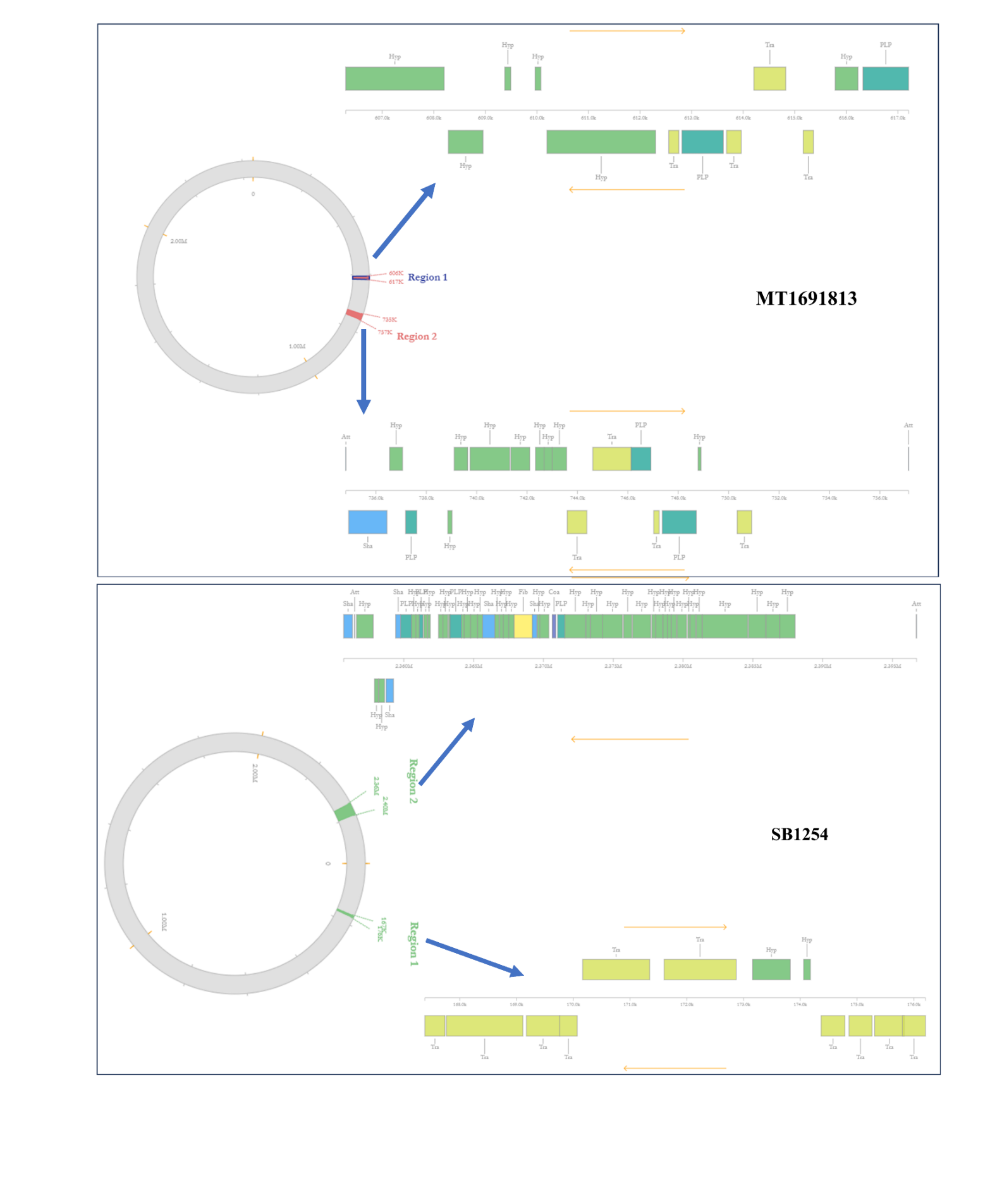


**Figure S14.** Prophages in the bacterial genomes: phage genomic content of the strains was studied using PHASTER tool. Bacteriophage harboring genes along with their predicted positions within the bacterial genome are represented here. Arrows are indicating the predicted genes within phage genomes. These strains were isolated from **saline water**.


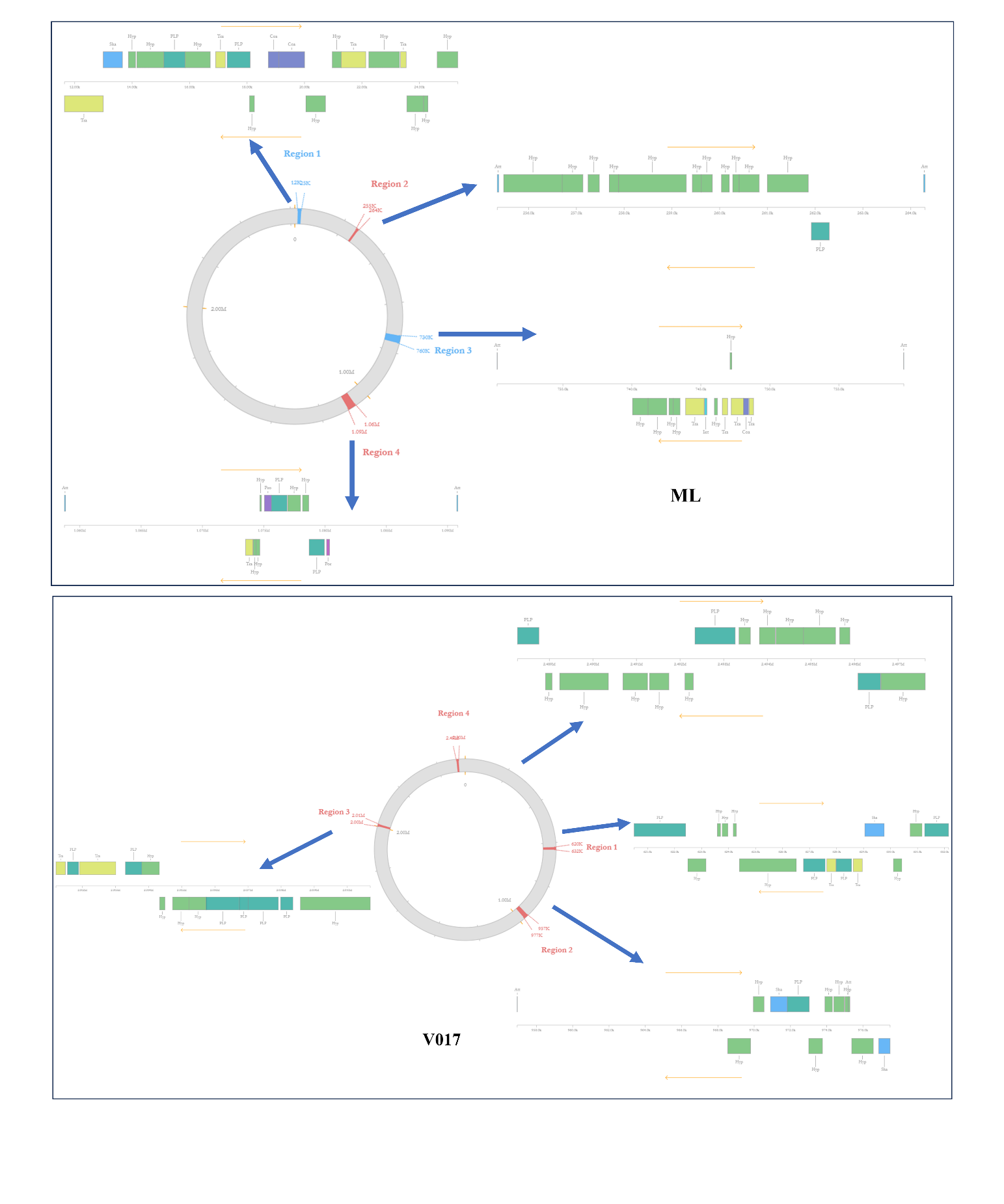


**Figure S15.** Prophages in the bacterial genomes: phage genomic content of the strains was studied using PHASTER tool. Bacteriophage harboring genes along with their predicted positions within the bacterial genome are represented here. Arrows are indicating the predicted genes within phage genomes. These strains were isolated from **soil**.


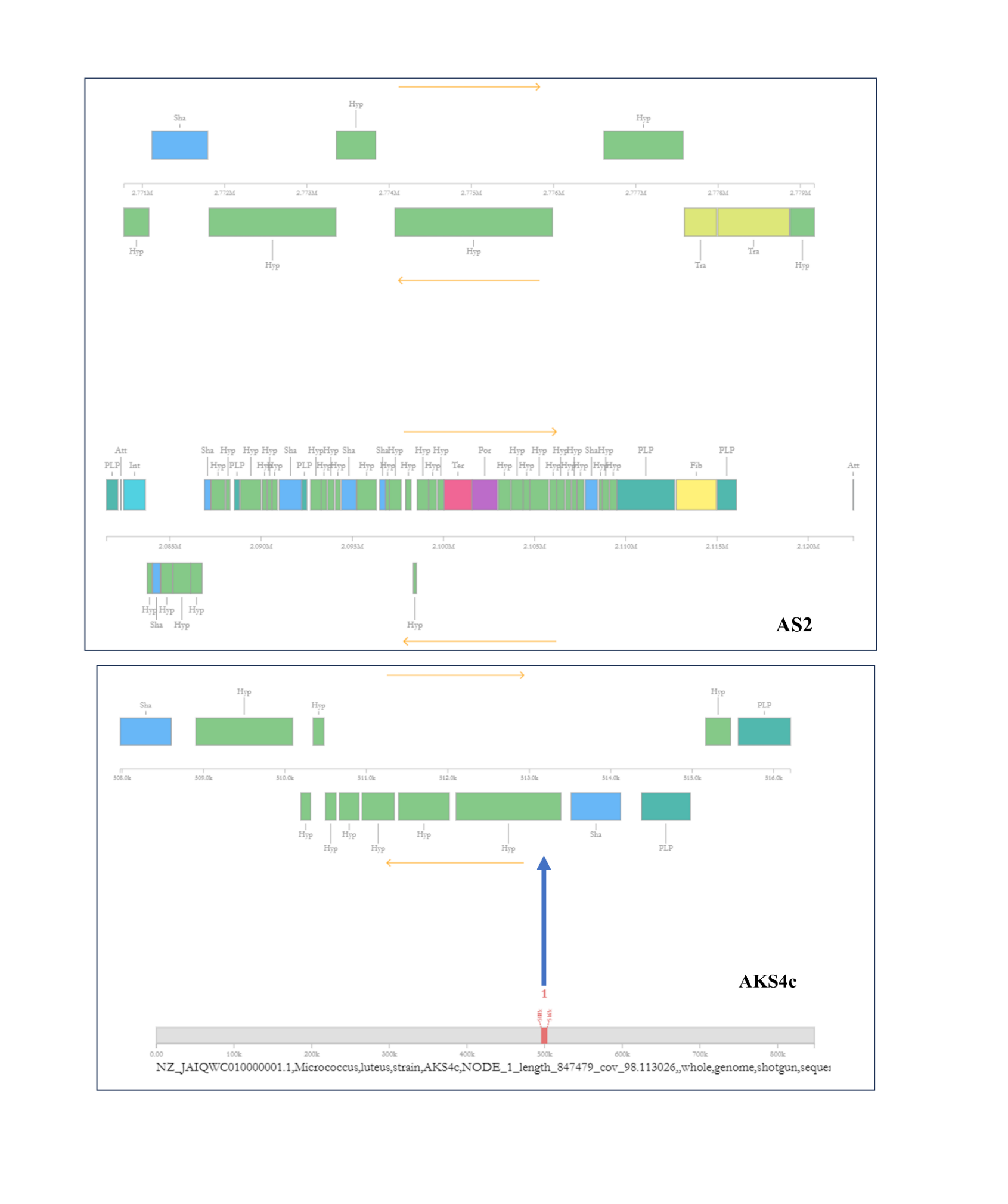


**Figure S16.** Prophages in the bacterial genomes: phage genomic content of the strains was studied using PHASTER tool. Bacteriophage harboring genes along with their predicted positions within the bacterial genome are represented here. Arrows are indicating the predicted genes within phage genomes. These strains were isolated from **contaminated water**.


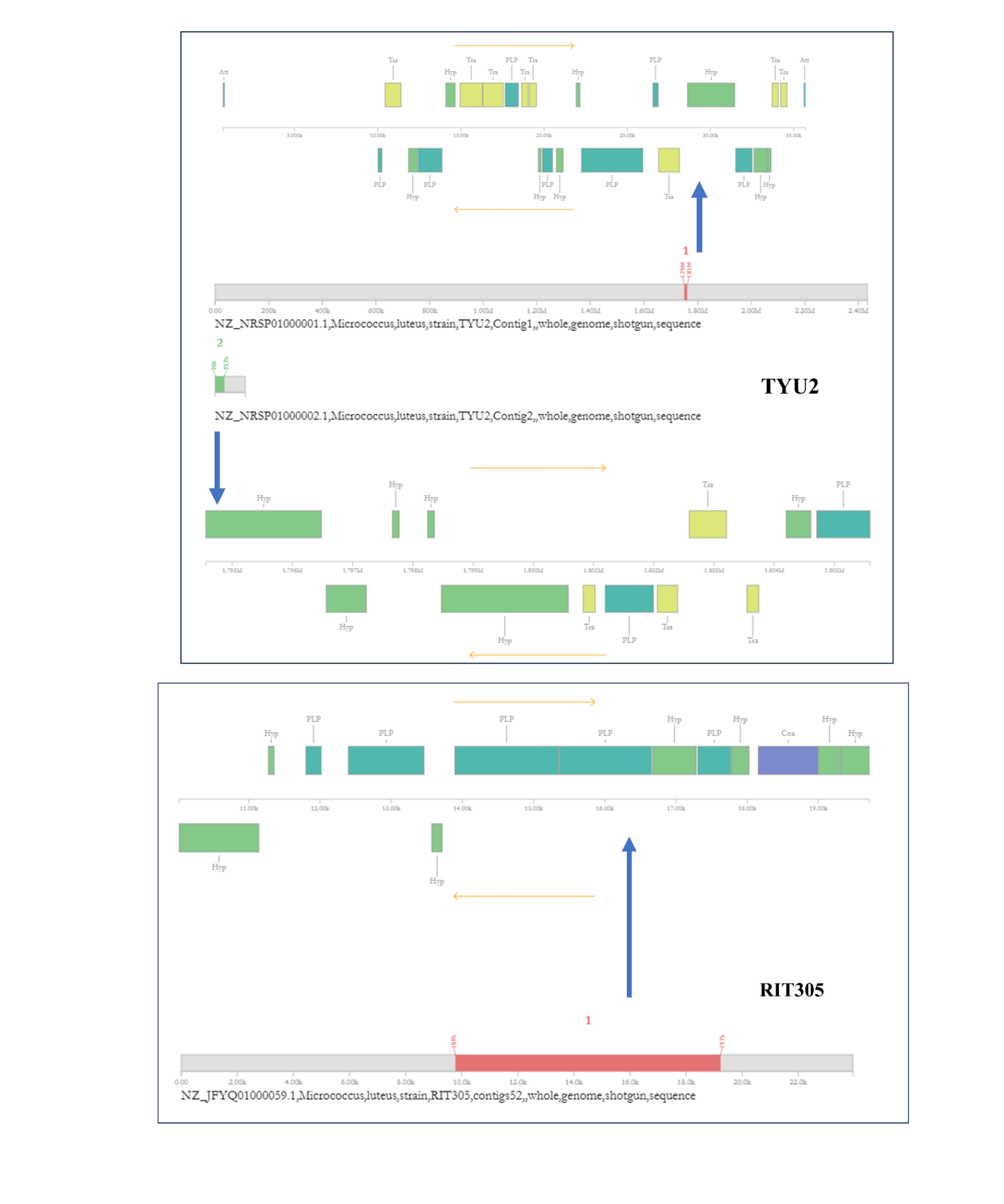


**Figure S17.** Prophages in the bacterial genomes: phage genomic content of the strains was studied using PHASTER tool. Bacteriophage harboring genes along with their predicted positions within the bacterial genome are represented here. Arrows are indicating the predicted genes within phage genomes. These strains were isolated from **plant**.


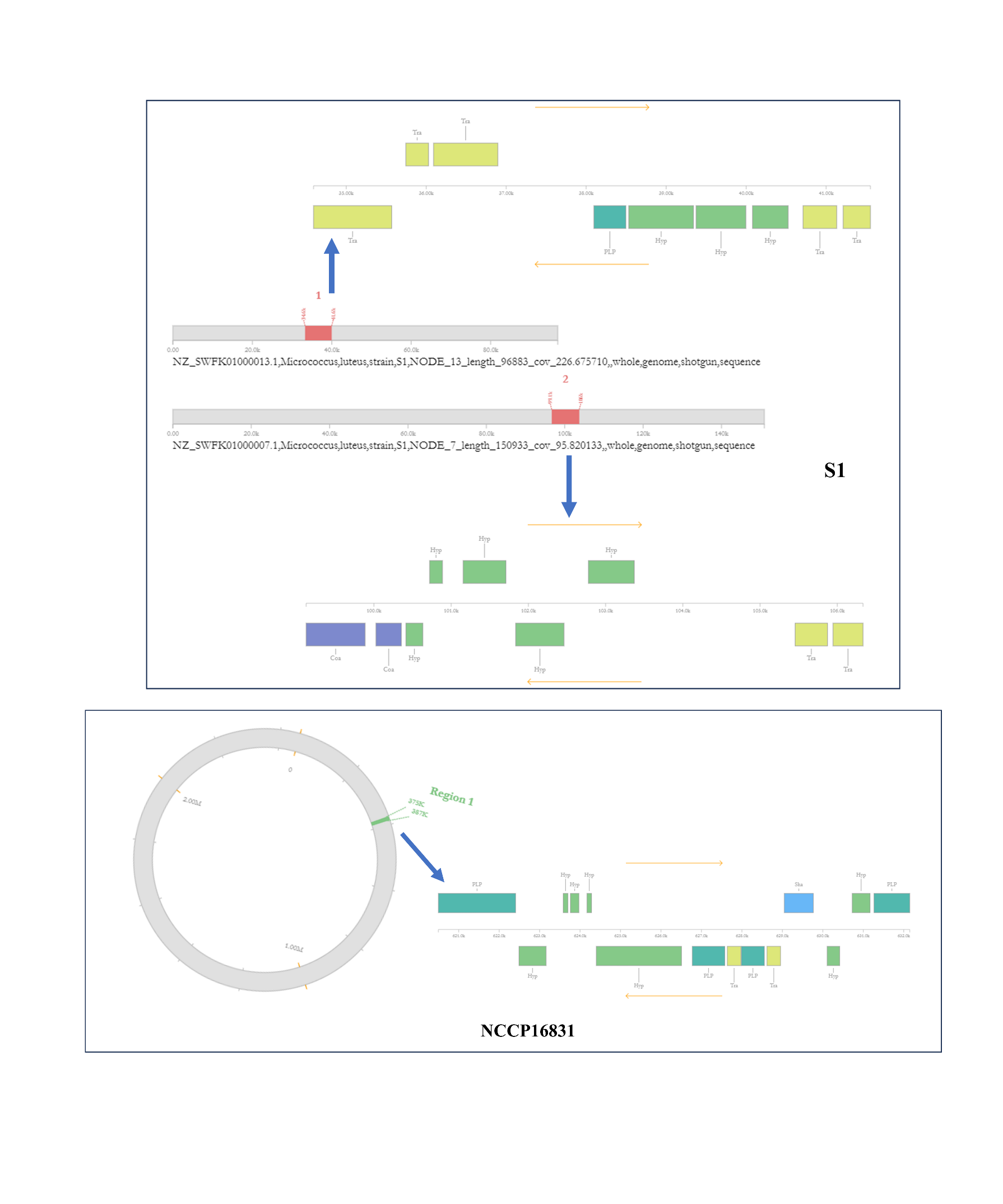


**Figure S18.** Prophages in the bacterial genomes: phage genomic content of the strains was studied using PHASTER tool. Bacteriophage harboring genes along with their predicted positions within the bacterial genome are represented here. Arrows are indicating the predicted genes within phage genomes. These strains were isolated from vertebrates.


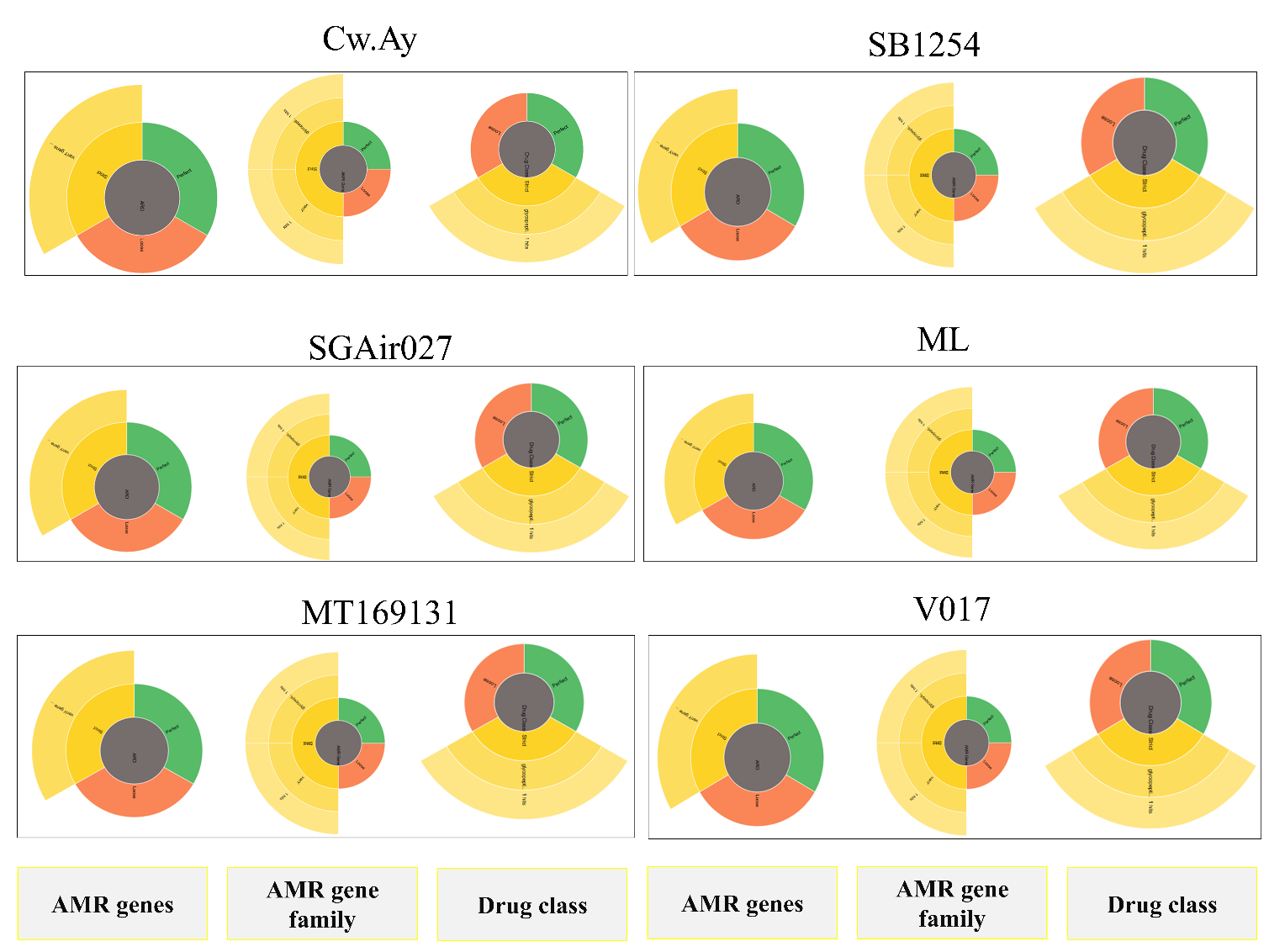


**Figure S19.** CARD analysis of the genomes (first 6 strains) for predicting antimicrobial resistance genes. Among three hits, here only strict hits were mentioned.


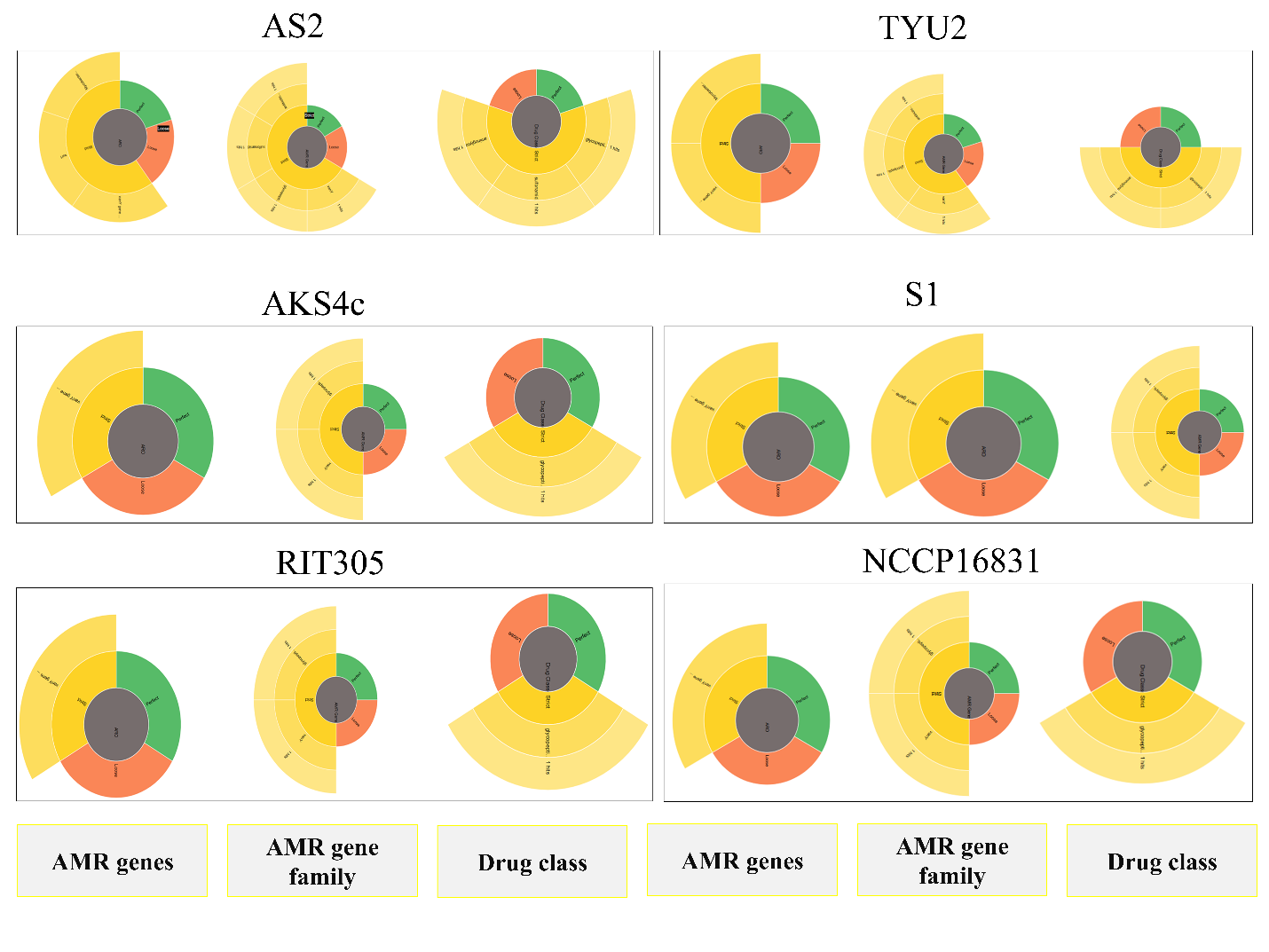


**Figure S20.** CARD analysis of the genomes (last 6 strains) for predicting antimicrobial resistance genes. Among three hits, here only strict hits were mentioned.

**Table S1** Comparative gene analysis among strains in context to heavy metal resistance; copy numbers are represented within brackets

| **Serial no.** | **Strains** | **Mercury resistance** | | **Copper resistance** | | **Cadmium resistance** | | **Arsenic resistance** | |
| --- | --- | --- | --- | --- | --- | --- | --- | --- | --- |
|  |  | **GIs** | **Core genome** | **GIs** | **Core genome** | **GIs** | **Core genome** | **GIs** | **Core genome** |
| 1 | Cw.Ay | *merA, merB, merR* | *MerR* (02)  *MerT*  *MerP*  *MerC*  *MerA* (02)  *MerD*  *MerE*  *MerB* | four-helix bundle copper-binding protein | *CRTR*  *CIA* (04)  *CopZ*  *MO*  *CopC*  *CopD*  *CopG*  *copS*  *copR*  *copCp* | cadmium resistance transporter | *czcD* (03)  *CzcA*  *CzcB*  *nccA*  *CRA* | *ArsC, ArsR, ArsB* | *arsR*  *arsD*  *arsA*  *arsB*  *arsC* (06)  *ACR3* (02)  *arsH*  *Perm*  *ArrA*  *ArrB*  *ArrS*  *ArsR2* |
| 2 | SGAir | *MerA* | *MerR* (02)  *MerT*  *MerP*  *MerC*  *MerA* (02)  *MerD*  *MerE*  *MerB* | multicopper oxidase family protein  four-helix bundle copper-binding protein  copper-translocating P-type ATPase | *CRTR*  *CIA* (07)  *CopZ* (03)  *CIfA*  *MO* (02)  *BCO*  *CT*  *CBPP*  *CRD*  *CopC*  *CopD*  *CopG*  *CRB*  *CSA*  *TR*  *copS*  *copR*  *copCp* | *cadA*,  cadmium transporter | *CZCR*  *czcD* (02)  *FP*  *nccB*  *TRCd*  *nccA* | *ArsR* | *arsR* (02)  *arsD*  *arsA*  *arsB*  *arsC* (03)  *ACR3*  *arsH*  *Perm*  *ArrA*  *ArrB*  *ArrS*  *ArsR2* |
| 3 | MT1691313 | *merA* | *MerR*  *MerT*  *MerP*  *MerC*  *MerA*  *MerD*  *MerE*  *MerB* | heme-copper oxidase subunit III  four-helix bundle copper-binding protein  copper-translocating P-type ATPase  multicopper oxidase family protein | *CRTR*  *CIA* (08)  *CopZ* (03)  *CIfA*  *MO*  *BCO*  *CT*  *CBPP*  *CRD*  *CopC*  *CopD*  *CopG*  *CRB*  *CSA*  *TR*  *copS*  *copR*  *copCp* | *cadA*,  cadmium resistance transporter | *CZCR*  *czcD* (02)  *CzcA*  *FP*  *CzcB*  *nccB*  *TRCd*  *nccA* | *ArsR* | *arsR*  *arsD*  *arsA*  *arsB*  *arsC* (03)  *ACR3*  *arsH*  *Perm*  *ArrA*  *ArrB*  *ArrS*  *ArsR2* |
| 4 | SB1254 | *merR, merA* | *MerT*  *MerP*  *MerC*  *MerA*  *MerD*  *MerE*  *MerB*  *MerR* | four-helix bundle copper-binding protein  copper-translocating P-type ATPase (CIA)  copper chaperone (CopZ)  copper oxidase | *CRTR*  *CIA* (04)  *CopZ* (03)  *CIfA*  *MO*  *BCO*  *CT*  *CBPP*  *CRD*  *CopC*  *CopD*  *CopG*  *CRB*  *CSA*  *TR*  *copS*  *copR*  *copCp* | *cadA*,  cadmium transporter | *czcD*  *CzcB*  *CzcC*  *nccA*  *TRMer* (03) | arsenic resistance protein,  *ArsC, ArsR, ArsB* | *arsR*  *arsD*  *arsA*  *arsB*  *arsC* (04)  *ACR3* (02)  *arsH*  *Perm*  *ArrA*  *ArrB*  *ArrS*  *ArsR2* |
| 5 | ML | *merA, merR* | *MerT*  *MerC*  *MerE*  *MerR* (04) | BlaI/MecI/CopY family transcriptional regulator | *CRTR*  *CIA* (03)  *CopZ* (02)  *ClfA*  *MO*  *BCO*  *CT*  *CBPP*  *CRD*  *CopC*  *CopD*  *CopG*  *CcmH*  *CcmF*  *CRB*  *CSA*  *TR*  *copS*  *copR*  *copCp* | NA | *CZCR*  *czcD* (03)  *FP*  *CzcB*  *nccB*  *nccA* | *ArsR* | *arsR*  *arsD*  *arsA*  *arsB*  *arsC* (03)  *ACR3*  *arsH*  *Perm*  *ArrA*  *ArrB*  *ArrS*  *ArsR2* |
| 6 | V017 | *merA, merR* | *MerR*  *MerT*  *MerP*  *MerC*  *MerA* (02)  *MerD*  *MerE*  *MerB* | four-helix bundle copper-binding protein  copper-translocating P-type ATPase  multicopper oxidase family protein (MO)  multicopper oxidase family protein | *CRTR*  *CIA* (08)  *CopZ* (04)  *CIfA*  *MO*  *BCO*  *CT*  *CBPP*  *CRD*  *CopC*  *CopD*  *CopG*  *CRB*  *CSA*  *TR*  *copS*  *copR*  *copCp* | cadmium resistance transporter | *CZCR*  *czcD* (03)  *CzcA*  *FP*  *nccB*  *TRCd*  *nccA*  *TRmer* | *ArsB, ArsR* | *arsR*  *ACR3* (02)  *arsD*  *arsA*  *arsB*  *arsC* (04)  *arsH*  *Perm*  *ArrA*  *ArrB*  *ArrS*  *ArsR2* |
| 7 | AS2 | *merA, merR* | *MerR* (02)  *MerT*  *MerP*  *MerC*  *MerA* (03)  *MerD*  *MerE*  *MerB* | multicopper oxidase family protein  four-helix bundle copper-binding protein  copper-translocating P-type ATPase | *CRTR*  *CIA* (03)  *CopZ* (02)  *MO*  *BCO*  *CT*  *CBPP*  *CopG*  *CRB*  *TR*  *copS*  *copR*  *copCp*  *ClfA* | *cadA*,  cadmium transporter  zinc-binding dehydrogenase | - | *ArsC, ArsB* | *-* |
| 8 | AKS4c | *merA, merB* | *MerR*  *MerA* (02)  *MerB*  *MerC*  *MerD*  *MerE*  *MerT*  *MerP* | four-helix bundle copper-binding protein | *CRTR*  *CIA* (05)  *CopZ*  *CIfA*  *MO*  *BCO*  *CT*  *CBPP*  *CRD*  *CopC*  *CopD*  *CopG*  *CRB*  *CSA*  *TR*  *copS*  *copR*  *copCp* | *cadA*,  cadmium transporter  cadmium resistance transporter | *CZCR*  *czcD (03)*  *CzcA*  *FP*  *CzcB*  *nccB*  *nccA* | *ArsC, ArsR, ArsB* | *arsR*  *arsD*  *arsA*  *arsB*  *arsC* (05)  *ACR3*  *arsH*  *Perm*  *ArrA*  *ArrB*  *ArrS*  *ArsR2* |
| 9 | TU02 | *merA* | *MerR* (02)  *MerT*  *MerP*  *MerC*  *MerA* (02*)*  *MerD*  *MerE*  *MerB* | four-helix bundle copper-binding protein  copper-translocating P-type ATPase  copper chaperone  copper oxidase | *CRTR*  *CIA* (08)  *CopZ* (03)  *CIfA*  *MO*  *BCO*  *CT*  *CBPP*  *CRD*  *CopC*  *CopD*  *CopG*  *CRB*  *CSA*  *TR*  *copS*  *copR*  *copCp* | cadmium transporter | *CZCR*  *czcD (4)*  *CzcA*  *FP*  *CzcB*  *TRCd*  *nccA* | *ArsB, ArsR* | *arsR*  *ACR3*  *arsD*  *arsA*  *arsB*  *arsC* (03)  *arsH*  *Perm*  *ArrA*  *ArrB*  *ArrS*  *ArsR2* |
| 10 | RIT305 | *merA, merR* | *MerR*  *MerT*  *MerP*  *MerC*  *MerA*  *MerD*  *MerE*  *MerB* | NA | *CRTR*  *CIA* (07)  *CopZ* (02)  *CIfA*  *MO*  *BCO*  *CT*  *CBPP*  *CRD*  *CopC*  *CopD*  *CopG*  *CRB*  *CSA*  *TR*  *copS*  *copR*  *copCp* | putative permease, CadD superfamily  putative CadD  putative cadmium-trans-porting ATPase  putative cadmium resistance transporter | *CZCR*  *czcD* (02)  *FP*  *CzcB*  *nccB*  *nccA* | *ArsR, ArsB* | *arsR*  *arsD*  *arsA*  *arsB*  *arsC* (03)  *ACR3*  *arsH*  *Perm*  *ArrA*  *ArrB*  *ArrS*  *ArsR2* |
| 11 | S1 | *merA, merB, merR* | *MerR* (03)  *MerT*  *MerP*  *MerC*  *MerA* (03)  *MerD*  *MerE*  *MerB* | multicopper oxidase family protein  four-helix bundle copper-binding protein  copper-translocating P-type ATPase | *CRTR*  *CIA* (04)  *CopZ* (03)  *CIfA*  *MO*  *BCO*  *CT*  *CBPP*  *CRD*  *CopC*  *CopD*  *CopG*  *CRB*  *CSA*  *TR*  *copS*  *copR*  *copCp* | cadmium transporter | *CZCR*  *czcD* (02)  *CzcA*  *CzcB*  *nccB*  *nccA* | arsenic resistance protein,  *ArsR* | *arsR*  *arsD*  *arsA*  *arsB*  *arsC* (05)  *ACR3* (02)  *arsH*  *Perm*  *ArrA*  *ArrB*  *ArrS*  *ArsR2* |
| 12 | NCCP16831 | *merA, merB* | *MerR*  *MerT*  *MerP*  *MerC*  *MerA*  *MerD*  *MerE*  *MerB* | four-helix bundle copper-binding protein  copper-translocating P-type ATPase  multicopper oxidase family protein | *CRTR*  *CIA* (04)  *CopZ* (02)  *CIfA*  *MO*  *BCO*  *CT*  *CBPP*  *CRD*  *CopC*  *CopD*  *CopG*  *CRB*  *CSA*  *TR*  *copS*  *copR*  *copCp* | *cadA*,  cadmium transporter | *CZCR*  *czcD* (02)  *CzcA*  *CzcB*  *TRCd* | *ArsR* | *arsR*  *arsD*  *arsA*  *arsB*  *arsC*  *ACR3*  *arsH*  *Perm*  *ArrA*  *ArrB*  *ArrS*  *ArsR2* |

| **Strains** | **Number of transposable elements** |
| --- | --- |
| Cw.Ay | 28 |
| SGAiR0127 | 72 |
| MT1691313 | 65 |
| SB1254 | 58 |
| ML | 69 |
| V017 | 76 |
| AS2 | 62 |
| AKS4c | 30 |
| RIT305 | 65 |
| TYU2 | 71 |
| S1 | 28 |
| NCCP16831 | 54 |

**Table S2** Number of transposons in the genomes of strains detected by ISfinder

**Table S3** Number of IS elements from different families across the strains

|  | Bacterial strains | | | | | | | | | | | |  |
| --- | --- | --- | --- | --- | --- | --- | --- | --- | --- | --- | --- | --- | --- |
| IS family | **AKS4c** | **AS2** | **Cw.Ay** | **ML** | **MT1691313** | **NCCP 16831** | **RIT305** | **S1** | **SB1254** | **SGAir0127** | **TYU2** | **V017** | **Total** |
| IS1380 | 0 | 0 | 0 | 0 | 1 | 1 | 2 | 0 | 0 | 1 | 2 | 2 | 9 |
| IS110 | 0 | 0 | 0 | 7 | 0 | 0 | 0 | 0 | 0 | 0 | 0 | 0 | 7 |
| IS1595 | 1 | 1 | 1 | 1 | 1 | 1 | 1 | 1 | 1 | 1 | 1 | 1 | 12 |
| IS21 | 0 | 0 | 0 | 0 | 2 | 0 | 2 | 0 | 2 | 2 | 0 | 2 | 10 |
| IS256 | 0 | 18 | 0 | 16 | 19 | 19 | 19 | 0 | 17 | 18 | 19 | 18 | 163 |
| IS3 | 10 | 13 | 6 | 16 | 12 | 13 | 12 | 11 | 13 | 11 | 14 | 14 | 145 |
| IS30 | 7 | 5 | 8 | 7 | 0 | 7 | 1 | 0 | 8 | 8 | 7 | 0 | 58 |
| IS481 | 5 | 18 | 8 | 14 | 11 | 8 | 8 | 12 | 12 | 10 | 8 | 20 | 134 |
| ISL3 | 0 | 0 | 0 | 3 | 14 | 0 | 14 | 0 | 0 | 15 | 13 | 14 | 73 |
| IS6 | 0 | 2 | 0 | 0 | 0 | 0 | 0 | 0 | 0 | 0 | 0 | 0 | 2 |
| IS5 | 3 | 0 | 0 | 0 | 0 | 1 | 0 | 1 | 1 | 0 | 1 | 0 | 7 |
| ISNCY | 2 | 3 | 3 | 3 | 3 | 3 | 3 | 2 | 3 | 3 | 3 | 3 | 34 |
| Tn3 | 2 | 3 | 2 | 2 | 2 | 1 | 2 | 1 | 1 | 3 | 3 | 2 | 24 |

**Table S4** List of plant growth promoting genes present within genomes and their names

| **Genes** | **Encoding proteins** | **References** |
| --- | --- | --- |
| Auxin biosynthesis | | |
| *trpD* | Anthranilate phosphoribosyltransferase | Kabiraj et al., 2023 |
| *trpB* | Tryptophan synthase subunit beta |  |
| *trpA* | Tryptophan synthase subunit alpha |  |
| *trpS* | Tryptophan-tRNA ligase |  |
| *trpC* | Tryptophan biosynthesis protein; provide necessary tryptophan for auxin biosynthesis |  |
| *trpD2* | Anthranilate phosphoribosyltransferase 2 |  |
| *trpE* | anthranilate synthase (transformation of chorismate into anthranilate) |  |
| *trpG* | anthranilate synthase component II |  |
| *Aec* | AEC family transporter |  |
| Phosphorous metabolism | | |
| *phoH* | *phoH* family protein | Kabiraj et al., 2023 |
| *phoR* | Sensory box histidine kinase *phoR* |  |
| *phoP* | Part of PhoP/PhoQ two-component system, regulate phosphate metabolism |  |
| *phoB* | Response regulator (detect environmental changes of phosphate level) |  |
| *phoD* | Alkaline phosphatase (Mineralization of organic phosphorous and make it available for plant utilization) |  |
| *pstA* | Phosphate ABC transporter permease |  |
| *pstB* | Phosphate ABC transporter ATP binding subunit |  |
| *pstC* | Phosphate ABC transporter permease subunit |  |
| *phoX* | Phosphate ABC transporter substrate-binding protein |  |
| *pstB* | Phosphate ABC transporter ATP-binding protein |  |
| *pstB1* |  |  |
| *pstS* | phosphate ABC transporter substrate-binding protein |  |
| *phoU* | Phosphate signaling complex protein |  |
| *phoU2* | Functions as *phoU* |  |
| Nitrogen metabolism | | |
| *nirB* | Nitrite reductase large subunit | Kabiraj et al., 2023 |
| *nirD* | Nitrite reductase small subunit |  |
| *narI* | Respiratory nitrate reductase subunit gamma |  |
| *narJ* | Nitrate reductase molybdenum cofactor assembly chaperone |  |
| *narH* | Nitrate reductase subunit beta |  |
| *narK* | Nitrate transporter |  |
| *nifD* | encode FeMo protein of nitrogenase enzyme |  |
| *nifK* | encode FeMo protein of nitrogenase enzyme | Adedayo and Babalola, 2023  Souza et al., 2015 |
| *nifH* | encode Fe- protein of nitrogenase enzyme | Souza et al., 2015 |
| *nodA* | Nodulation N-acetyltransferase |  |
| *nodB* | Chitooligosaccharide deacetylase (enzymes involved in synthesis of Nod factor) |  |
| *nodC* | Chitooligosaccharide synthase/N-acetylglucosaminyltransferase |  |
| *nodD* | Nodulation protein D |  |
|  | | |
| *iaah* | indole-3-acetamide hydrolase | Souza et al., 2015 |
| *ipt* | isopentenyl transferase |  |
| Phosphate solubilization | |  |
| *pqqB* | Genes are associated with pyrroloquinoline synthesis | Bruto et al., 2014 |
| *pqqC* |  |  |
| *pqqD* |  |  |
| *pqqE* |  |  |
| *pqqF* |  |  |
| *pqqG* |  |  |
| Auxin synthesis | |  |
| *ipdC* | indole-3-pyruvate decarboxylase |  |
| *ppdC* |  |  |
| *ipdC*/*ppdC* | Production of IAA |  |
| *KynA* | tryptophan 2,3-dioxygenase (conversion of other AA to trp) |  |
| *KynB* | kynurenine formamidase (conversion of other AA to trp) |  |
| *KynU* | Kynureninase (conversion of other AA to trp) |  |
| Nitrate transporter | |  |
| *narT* | Associated with encoding proteins which are responsible for transporting nitrate | Bruto et al., 2014 |
| *narY* |  |  |
| *narG* |  |  |
| *nirC* |  |  |
| Siderophore biosynthesis | | Singh et al., 2022 |
| *entB* | enterobactin biosynthesis |  |
| *iroB* | salmochelin biosynthesis |  |
| *entS* | enterobactin synthesis |  |
| *fep*A, *fep*B, *fep*C,  *fep*D, *fep*E, *fep*G, *fes*, and *ent*S | enterobactin uptake and utilization |  |
| *irp*1 and *irp*2 | Siderophore synthesis |  |
| *acdS* | ACC-deaminase; upregulate stress resistance by increasing ethylene concentration in plants |  |
| *fepD* | Ferric enterobactin ABC transporter membrane subunit |  |
| Urea metabolism | | |
| *ureC* | urease subunit alpha | Goswami et al., 2015 |
| *ureE* | urease accessory protein UreE |  |
| *ureG* | urease accessory protein UreG |  |
| Sulfur assimilation genes | | |
| cysD | Sulfate adenylyltransferase subunit CysD | Huang et al., 2022 |
| cysK | cysteine synthase A | Takumi et al., 2016 |
| Aromatic degradation pathway-related genes | | |
| *paaA* | 1,2-phenylacetyl-CoA epoxidase subunit PaaA | Fernández et al., 2006 |
| *paaB* | 1,2-phenylacetyl-CoA epoxidase subunit PaaB |  |
| *paaC* | 1,2-phenylacetyl-CoA epoxidase subunit PaaC |  |
| *padD* | 1,2-phenylacetyl-CoA epoxidase subunit PaaD |  |
| *paaE* | 1,2-phenylacetyl-CoA epoxidase subunit PaaE |  |
| *paaZ* | phenylacetic acid degradation bifunctional protein PaaZ |  |
| *hpaE* | 5-carboxymethyl-2-hydroxymuconate semialdehyde dehydrogenase | Rico-Jiménez et al., 2023 |
| *hpaD* | 3,4-dihydroxyphenylacetate 2,3-dioxygenase |  |

**Table S5** Comparative gene analysis across strains related to plant growth promoting traits

| Gene | CW.Ay | SGAir0127 | MT1691313 | SB1254 | ML | V017 | AS2 | AKS4c | RIT305 | TYU2 | S1 | NCCP16831 |
| --- | --- | --- | --- | --- | --- | --- | --- | --- | --- | --- | --- | --- |
| Auxin biosynthesis | | | | | | | | | | | | |
| *trpD* | 1 | 1 | 1 | 1 | 1 | 1 | 1 | 1 | 1 | 1 | 1 | 1 |
| *trpB* | 2 | 2 | 2 | 2 | 3 | 2 | 1 | 2 | 3 | 2 | 2 | 2 |
| *trpA* | 1 | 1 | 1 | 1 | 1 | 1 | 1 | 1 | 1 | 1 | 1 | 1 |
| *trpS* | 1 | 1 | 1 | 1 | 1 | 1 | 1 | 1 | 1 | 1 | 1 | 1 |
| *trpC* | 1 | 1 | 1 | 1 | 1 | 1 | 1 | 1 | 1 | 1 | 1 | 1 |
| *trpD2* | 0 | 0 | 0 | 0 | 0 | 0 | 0 | 0 | 0 | 0 | 0 | 0 |
| *trpE* | 0 | 0 | 0 | 0 | 0 | 0 | 0 | 0 | 0 | 0 | 0 | 0 |
| *trpG* | 0 | 0 | 0 | 0 | 0 | 0 | 0 | 0 | 0 | 0 | 0 | 0 |
| *Aec* | 1 | 1 | 1 | 1 | 1 | 0 | 0 | 1 | 1 | 1 | 1 | 1 |
| Phosphorous metabolism | | | | | | | | | | | | |
| *phoH* | 2 | 2 | 2 | 0 | 2 | 2 | 2 | 2 | 2 | 2 | 2 | 2 |
| *phoR* | 0 | 0 | 0 | 0 | 0 | 0 | 0 | 0 | 0 | 0 | 0 | 0 |
| *phoP* | 0 | 0 | 0 | 0 | 0 | 0 | 0 | 0 | 0 | 0 | 0 | 0 |
| *phoB* | 0 | 0 | 0 | 0 | 0 | 0 | 0 | 0 | 0 | 0 | 0 | 0 |
| *phoD* | 0 | 0 | 0 | 0 | 0 | 0 | 0 | 0 | 0 | 0 | 0 | 0 |
| *pstA* | 1 | 1 | 1 | 1 | 1 | 1 | 1 | 1 | 1 | 1 | 1 | 1 |
| *pstB* | 1 | 1 | 1 | 1 | 1 | 1 | 1 | 1 | 1 | 1 | 1 | 1 |
| *pstC* | 1 | 1 | 1 | 1 | 1 | 1 | 1 | 1 | 1 | 1 | 1 | 1 |
| *phoX* | 1 | 1 | 1 | 1 | 1 | 1 | 1 | 1 | 2 | 1 | 1 | 1 |
| *pstS* | 1 | 1 | 1 | 0 | 1 | 1 | 0 | 0 | 1 | 1 | 1 | 1 |
| *phoU* | 1 | 1 | 1 | 1 | 1 | 1 | 1 | 1 | 1 | 1 | 1 | 1 |
| Nitrogen metabolism | | | | | | | | | | | | |
| *nirB* | 0 | 0 | 0 | 0 | 0 | 0 | 0 | 0 | 0 | 0 | 0 | 0 |
| *nirD* | 0 | 0 | 0 | 0 | 0 | 0 | 0 | 0 | 0 | 0 | 0 | 0 |
| *narI* | 0 | 0 | 0 | 0 | 0 | 0 | 0 | 0 | 0 | 0 | 0 | 0 |
| *narJ* | 0 | 0 | 0 | 0 | 0 | 0 | 0 | 0 | 0 | 0 | 0 | 0 |
| *narH* | 0 | 0 | 0 | 0 | 0 | 0 | 0 | 0 | 0 | 0 | 0 | 0 |
| *narK* | 0 | 0 | 0 | 0 | 0 | 0 | 0 | 0 | 0 | 0 | 0 | 0 |
| *nifD* | 0 | 0 | 0 | 0 | 0 | 0 | 0 | 0 | 0 | 0 | 0 | 0 |
| *nifK* | 0 | 0 | 0 | 0 | 0 | 0 | 0 | 0 | 0 | 0 | 0 | 0 |
| *nifH* | 0 | 0 | 0 | 0 | 0 | 0 | 0 | 0 | 0 | 0 | 0 | 0 |
| *nodA* | 0 | 0 | 0 | 0 | 0 | 0 | 0 | 0 | 0 | 0 | 0 | 0 |
| *nodB* | 0 | 0 | 0 | 0 | 0 | 0 | 0 | 0 | 0 | 0 | 0 | 0 |
| *nodC* | 0 | 0 | 0 | 0 | 0 | 0 | 0 | 0 | 0 | 0 | 0 | 0 |
| *nodD* | 0 | 0 | 0 | 0 | 0 | 0 | 0 | 0 | 0 | 0 | 0 | 0 |
|  |  |  |  |  |  |  |  |  |  |  |  |  |
| *iaah* | 0 | 0 | 0 | 0 | 0 | 0 | 0 | 0 | 0 | 0 | 0 | 0 |
| *ipt* | 0 | 0 | 0 | 0 | 0 | 0 | 0 | 0 | 0 | 0 | 0 | 0 |
| Phosphate solubilization | | | | | | | | | | | | |
| *pqqB* | 0 | 0 | 0 | 0 | 0 | 0 | 0 | 0 | 0 | 0 | 0 | 0 |
| *pqqC* | 0 | 0 | 0 | 0 | 0 | 0 | 0 | 0 | 0 | 0 | 0 | 0 |
| *pqqD* | 2 | 2 | 2 | 2 | 2 | 2 | 2 | 2 | 2 | 2 | 2 | 2 |
| *pqqE* | 0 | 0 | 0 | 0 | 0 | 0 | 0 | 0 | 0 | 0 | 0 | 0 |
| *pqqF* | 0 | 0 | 0 | 0 | 0 | 0 | 0 | 0 | 0 | 0 | 0 | 0 |
| *pqqG* | 0 | 0 | 0 | 0 | 0 | 0 | 0 | 0 | 0 | 0 | 0 | 0 |
| Auxin synthesis | | | | | | | | | | | | |
| *ipdC* | 0 | 0 | 0 | 0 | 0 | 0 | 0 | 0 | 0 | 0 | 0 | 0 |
| *ppdC* | 0 | 0 | 0 | 0 | 0 | 0 | 0 | 0 | 0 | 0 | 0 | 0 |
| *ipdC*/*ppdC* | 0 | 0 | 0 | 0 | 0 | 0 | 0 | 0 | 0 | 0 | 0 | 0 |
| *KynA* | 0 | 0 | 0 | 0 | 0 | 0 | 0 | 0 | 0 | 0 | 0 | 0 |
| *KynB* | 0 | 0 | 0 | 0 | 0 | 0 | 0 | 0 | 0 | 0 | 0 | 0 |
| *KynU* | 0 | 0 | 0 | 0 | 0 | 0 | 0 | 0 | 0 | 0 | 0 | 0 |
| Nitrate transporter | | | | | | | | | | | | |
| *narT* | 0 | 0 | 0 | 0 | 0 | 0 | 0 | 0 | 0 | 0 | 0 | 0 |
| *narY* | 0 | 0 | 0 | 0 | 0 | 0 | 0 | 0 | 0 | 0 | 0 | 0 |
| *narG* | 0 | 0 | 0 | 0 | 0 | 0 | 0 | 0 | 0 | 0 | 0 | 0 |
| *nirC* | 0 | 0 | 0 | 0 | 0 | 0 | 0 | 0 | 0 | 0 | 0 | 0 |
| Siderophore biosynthesis | | | | | | | | | | | | |
| *entB* | 0 | 0 | 0 | 0 | 0 | 0 | 0 | 0 | 0 | 0 | 0 | 0 |
| *iroB* | 0 | 0 | 0 | 0 | 0 | 0 | 0 | 0 | 0 | 0 | 0 | 0 |
| *entS* | 0 | 0 | 0 | 0 | 0 | 0 | 0 | 0 | 0 | 0 | 0 | 0 |
| *fep*A, *fep*B, *fep*C,  *fep*D, *fep*E, *fep*G | 0 | 0 | 0 | 0 | 0 | 0 | 0 | 0 | 0 | 0 | 0 |  |
| *irp*1 and *irp*2 | 0 | 0 | 0 | 0 | 0 | 0 | 0 | 0 | 0 | 0 | 0 | 0 |
| *acdS* | 0 | 0 | 0 | 0 | 0 | 0 | 0 | 0 | 0 | 0 | 0 | 0 |
| IucA/Iucc family siderophore biosynthesis protein | 0 | 0 | 0 | 0 | 0 | 0 | 0 | 1 | 0 | 0 | 0 | 0 |
| Siderophore interacting protein | 1 | 1 | 1 | 1 | 1 | 1 | 1 | 1 | 1 | 1 | 1 | 1 |
| Iron-siderophore ABC transporter | 1 | 1 | 1 | 1 | 1 | 1 | 1 | 1 | 1 | 1 | 1 | 1 |
| *hemQ* | 0 | 0 | 1 | 0 | 0 | 0 | 0 | 0 | 0 | 1 | 0 | 0 |
| *hemE* | 0 | 1 | 1 | 1 | 1 | 1 | 1 | 1 | 1 | 1 | 1 | 1 |
| *ureC* | 1 | 1 | 1 | 1 | 1 | 1 | 1 | 1 | 1 | 1 | 1 | 1 |
| *ureE* | 1 | 1 | 1 | 1 | 1 | 1 | 1 | 1 | 1 | 1 | 1 | 1 |
| *ureG* | 1 | 1 | 1 | 1 | 1 | 1 | 1 | 1 | 1 | 1 | 1 | 1 |
| Aromatic degradation pathway related genes | | | | | | | | | | | | |
| *paaA* | 1 | 1 | 1 | 1 | 1 | 1 | 1 | 1 | 1 | 1 | 1 | 1 |
| *paaB* | 1 | 1 | 1 | 1 | 1 | 1 | 1 | 1 | 1 | 1 | 1 | 1 |
| *paaC* | 1 | 1 | 1 | 1 | 1 | 1 | 1 | 1 | 1 | 1 | 1 | 1 |
| *padD* | 1 | 1 | 1 | 1 | 1 | 1 | 1 | 1 | 1 | 1 | 1 | 1 |
| *paaE* | 1 | 1 | 1 | 1 | 1 | 1 | 1 | 1 | 1 | 1 | 1 | 1 |
| *paaZ* | 1 | 1 | 1 | 1 | 1 | 1 | 1 | 1 | 1 | 1 | 1 | 1 |
| *hpaE* | 1 | 1 | 1 | 1 | 1 | 1 | 1 | 1 | 1 | 1 | 1 | 1 |
| *hpaD* | 1 | 1 | 1 | 1 | 1 | 1 | 1 | 1 | 1 | 1 | 1 | 1 |

**Table S6** Phage genome prediction in the genomes of the strains, their completeness, GC% and functional genes

| **Source** | **Genome** | **Region length** | **Completeness** | **Score** | **Total Proteins** | **Most common phages** | **GC%** | **Specific Keyword** |
| --- | --- | --- | --- | --- | --- | --- | --- | --- |
| Vertebrates | NCCP 16831 | 12.3 Kb | intact | 130 | 14 | PHAGE_Paenib_Tripp_NC_028930(3) | 66.88 | Transposase |
|  | S1 | 6.9 Kb | incomplete | 60 | 9 | PHAGE_Paenib_Tripp_NC_028930(3) | 66.68 | Transposase |
|  |  | 7.2 Kb | incomplete | 50 | 9 | PHAGE_Paenib_Tripp_NC_028930(2) | 64.82 | head, transposase |
| Sea water | MT1691313 | 10.9Kb | incomplete | 60 | 12 | PHAGE_Klebsi_ST147_VIM1phi7.1_NC_049451(1) | 68.06 | transposase, lysin |
|  |  | 22.3 Kb | incomplete | 60 | 17 | PHAGE_Escher_520873_NC_049344(2) | 68.97 | tail, transposase |
|  | SB1254 | 8.8 Kb | intact | 110 | 12 | PHAGE_Paenib_Tripp_NC_028930(3) | 66.41 | transposase |
|  |  | 41 Kb | intact | 110 | 50 | PHAGE_Arthro_Maja_NC_048140(17) | 71.07 | tail, capsid |
| Soil | ML | 13.6 Kb | questionable | 90 | 19 | PHAGE_Stx2_II_NC_004914(2) | 66.08 | transposase, tail, lysin, head |
|  |  | 8.9 Kb | incomplete | 10 | 12 | PHAGE_Mycoba_Heldan_NC_042328(2) | 72.88 | NA |
|  |  | 29.4Kb | questionable | 70 | 12 | PHAGE_Paenib_Tripp_NC_028930(2) | 68.67 | transposase, integrase, head |
|  |  | 32 Kb | incomplete | 50 | 10 | PHAGE_Mycoba_Phayonce_NC_028796(1) | 66.96 | transposase, protease, portal |
|  | V017 | 11.6 Kb | incomplete | 60 | 14 | PHAGE_Entero_VT2phi_272_NC_028656(1) | 68.31 | lysin, transposase, tail |
|  |  | 20.5Kb | incomplete | 30 | 10 | PHAGE_Gordon_Fairfaxidum_NC_048185(2) | 72.12 | tail |
|  |  | 9.4Kb | incomplete | 40 | 13 | PHAGE_Bacill_G_NC_023719(2) | 70.06 | transposase, lysin |
|  |  | 9.3 Kb | incomplete | 10 | 14 | PHAGE_Sinorh_phiM7_NC_041929(1) | 72.94 | NA |
| Air | SGAir0127 | 8.4 Kb | questionable | 90 | 10 | PHAGE_Entero_fiAA91_ss_NC_022750(2) | 67.44 | transposase |
|  |  | 7.9 Kb | incomplete | 50 | 7 | PHAGE_Escher_520873_NC_049344(2) | 68.67 | tail, transposase |
|  | Cw.Ay | 7.5 Kb | incomplete | 50 | 6 | PHAGE_Gordon_Cucurbita_NC_031029(2) | 62.86 | transposase |
| Plant | TYU2 | 11 Kb | incomplete | 60 | 12 | PHAGE_Burkho_phiE125_NC_003309(1) | 67.98 | transposase, lysin |
|  |  | 34.9 Kb | intact | 100 | 23 | PHAGE_Paenib_Tripp_NC_028930(3) | 66.3 | transposase |
|  | RIT305 | 9.6 Kb | incomplete | 20 | 13 | PHAGE_Strept_phiSASD1_NC_014229(2) | 68.27 | capsid |
| Industry or Contaminated Water | AKS4c | 8.2 Kb | incomplete | 30 | 13 | PHAGE_Gordon_SoilAssassin_NC_031251(2) | 70.20 | tail |
|  | AS2 | 40.9 Kb | intact | 130 | 49 | PHAGE_Arthro_vB_ArS_ArV2_NC_022972(8) | 71.68 | integrase, tail, terminase, portal |
|  |  | 8.3 Kb | incomplete | 40 | 9 | PHAGE_Gordon_Ruthy_NC_048019(1) | 72.47 | tail, transposase |

**Table S8** CARD analyses of selected genomes and associated resistance genes, family, class, etc. (only strict hit is considered here)

| **Strain name** | **RGI**  **Criteria** | **ARO Term** | **Detection**  **Criteria** | **AMR**  **Gene Family** | **Drug**  **Class** | **Resistance**  **Mechanism** | **% Identity of Matching Region** | **% Length of Reference Sequence** |
| --- | --- | --- | --- | --- | --- | --- | --- | --- |
| Cw.Ay | Strict | *vanY* gene in *vanF* cluster | protein homolog model | *vanY*, glycopeptide resistance gene cluster | glycopeptide antibiotic | antibiotic target alteration | 34.46 | 84.3 |
| MT169131 | Strict | *vanY* gene in *vanM* cluster | protein homolog model | *vanY*, glycopeptide resistance gene cluster | glycopeptide antibiotic | antibiotic target alteration | 34.1 | 106.1 |
| SgAir027 | Strict | *vanY* gene in *vanM* cluster | protein homolog model | *vanY*, glycopeptide resistance gene cluster | glycopeptide antibiotic | antibiotic target alteration | 34.01 | 106.87 |
| SB1294 | Strict | *vanY* gene in *vanA*  cluster | protein homolog model | *vanY*, glycopeptide resistance gene cluster | glycopeptide antibiotic | antibiotic target alteration | 36.75 | 81.52 |
| ML | Strict | *vanY* gene in *vanM* cluster | protein homolog model | *vanY*, glycopeptide resistance gene cluster | glycopeptide antibiotic | antibiotic target alteration | 34.01 | 106.87 |
| V017 | Strict | *vanY* gene in *vanM* cluster | protein homolog model | *vanY*, glycopeptide resistance gene cluster | glycopeptide antibiotic | antibiotic target alteration | 33.33 | 106.01 |
| AS2 | Strict | *vanY* gene in *vanM* cluster | protein homolog model | *vanY*, glycopeptide resistance gene cluster | glycopeptide antibiotic | antibiotic target alteration | 30.61 | 108.58 |
|  | Strict | *sul1* | protein homolog model | sulfonamide resistant *sul* | sulfonamide antibiotic | antibiotic target replacement | 100 | 101.43 |
|  | Strict | *Mycobacterium tuberculosis* rpsL mutations conferring resistance to Streptomycin | protein variant model | antibiotic-resistant *rpsL* | aminoglycoside antibiotic | antibiotic target alteration | 83.74 | 100 |
| AKS4c | Strict | *vanY* gene in *vanA*  cluster | protein homolog model | *vanY*, glycopeptide resistance gene cluster | glycopeptide antibiotic | antibiotic target alteration | 36.75 | 81.52 |
| RIT305 | Strict | *vanY* gene in *vanA*  cluster | protein homolog model | *vanY*, glycopeptide resistance gene cluster | glycopeptide antibiotic | antibiotic target alteration | 36.75 | 81.52 |
| TYU2 | Strict | *vanY* gene in *vanM* cluster | protein homolog model | *vanY*, glycopeptide resistance gene cluster | glycopeptide antibiotic | antibiotic target alteration | 34.01 | 106.87 |
|  | Strict | Mycobacterium tuberculosis rpsL mutations conferring resistance to Streptomycin | protein variant model | antibiotic-resistant *rpsL* | aminoglycoside antibiotic | antibiotic target alteration | 82.93 | 100 |
| S1 | Strict | *vanY* gene in *vanM* cluster | protein homolog model | *vanY,* glycopeptide resistance gene cluster | glycopeptide antibiotic | antibiotic target alteration | 34.69 | 106.87 |
| NCCP16831 | Strict | *vanY* gene in *vanM* cluster | protein homolog model | *vanY*, glycopeptide resistance gene cluster | glycopeptide antibiotic | antibiotic target alteration | 33.33 | 106.01 |

**References**

Adedayo, A. A., &amp; Babalola, O. O. (2023). Genomic mechanisms of plant growth-promoting bacteria in the production of leguminous crops. *Frontiers in Genetics*, 14, 1276003.

Bruto, M., Prigent-Combaret, C., Muller, D., and Moënne-Loccoz, Y. (2014). Analysis of genes contributing to plant-beneficial functions in plant growth-promoting rhizobacteria and related Proteobacteria. *Scientific reports* 4, 6261.

Fernández, C., Ferrández, A., Minambres, B., Díaz, E., and García, J. L. (2006). Genetic characterization of the phenylacetyl-coenzyme A oxygenase from the aerobic phenylacetic acid degradation pathway of *Escherichia coli*. *Applied and environmental microbiology*, 72, 7422-7426.

Goswami, D., Patel, K., Parmar, S., Vaghela, H., Muley, N., Dhandhukia, P., & Thakker, J. N. (2015). Elucidating multifaceted urease producing marine Pseudomonas aeruginosa BG as a cogent PGPR and bio-control agent. *Plant growth regulation*, 75, 253-263.

Huang, X., Zeng, Z., Chen, Z., Tong, X., Jiang, J., He, C., & Xiang, T. (2022). Deciphering the potential of a plant growth promoting endophyte Rhizobium sp. WYJ-E13, and functional annotation of the genes involved in the metabolic pathway. *Frontiers in Microbiology*, 13, 1035167.

Kabiraj, A., Halder, U., Panja, A. S., Chitikineni, A., Varshney, R. K., & Bandopadhyay, R. (2023). Detailed genomic and biochemical characterization and plant growth promoting properties of an arsenic-tolerant isolate of *Bacillus pacificus* from contaminated groundwater of West Bengal, India. *Biocatalysis and Agricultural Biotechnology*, 52, 102825.

Rico‐Jiménez, M., Muñoz‐Mira, S., Lomas‐Martínez, C., Krell, T., & Matilla, M. A. (2023). Regulation of indole‐3‐acetic acid biosynthesis and consequences of auxin production deficiency in *Serratia plymuthica*. *Microbial Biotechnology*, 16, 1671-1689.

Singh, P., Chauhan, P. K., Upadhyay, S. K., Singh, R. K., Dwivedi, P., Wang, J., et al. (2022). Mechanistic insights and potential use of siderophores producing microbes in rhizosphere for mitigation of stress in plants grown in degraded land. *Frontiers in Microbiology*, 13, 898979.

Souza, R. D., Ambrosini, A., &amp; Passaglia, L. M. (2015). Plant growth-promoting bacteria as inoculants in agricultural soils. *Genetics and molecular biology*, 38, 401-419.

Takumi, K., & Nonaka, G. (2016). Bacterial cysteine-inducible cysteine resistance systems. *Journal of bacteriology*, 198, 1384-1392.

Timofeeva, A. M., Galyamova, M. R., &amp; Sedykh, S. E. (2023). Plant growth-promoting soil bacteria: Nitrogen fixation, phosphate solubilization, siderophore production, and other biological activities. *Plants*, 12, 4074.
